# A modular degron library for synthetic circuits in mammalian cells

**DOI:** 10.1038/s41467-019-09974-5

**Published:** 2019-05-01

**Authors:** Hélène Chassin, Marius Müller, Marcel Tigges, Leo Scheller, Moritz Lang, Martin Fussenegger

**Affiliations:** 1 Department of Biosystems Science and Engineering, ETH Zürich, Mattenstrasse 26, CH-4058 Basel, Switzerland; 2 Cilag AG, Hochstrasse 201, CH-8200 Schaffhausen, Switzerland; 3 Institute of Science and Technology Austria, A-3400 Klosterneuburg, Austria; 4 Faculty of Science, University of Basel, Mattenstrasse 26, CH-4058 Basel, Switzerland

**Keywords:** Genetic circuit engineering, Synthetic biology

## Abstract

Tight control over protein degradation is a fundamental requirement for cells to respond rapidly to various stimuli and adapt to a fluctuating environment. Here we develop a versatile, easy-to-handle library of destabilizing tags (degrons) for the precise regulation of protein expression profiles in mammalian cells by modulating target protein half-lives in a predictable manner. Using the well-established tetracycline gene-regulation system as a model, we show that the dynamics of protein expression can be tuned by fusing appropriate degron tags to gene regulators. Next, we apply this degron library to tune a synthetic pulse-generating circuit in mammalian cells. With this toolbox we establish a set of pulse generators with tailored pulse lengths and magnitudes of protein expression. This methodology will prove useful in the functional roles of essential proteins, fine-tuning of gene-expression systems, and enabling a higher complexity in the design of synthetic biological systems in mammalian cells.

## Abstract (web summary)

One method of controlling protein degradation is through the use of degrons. Here the authors present a toolbox of characterised degrons as a library to fine-tune biological gene-expression systems. Its application is demonstrated by a set of tunable synthetic pulse generators in mammalian cells.

## Introduction

Single and multicellular organisms must tightly control the levels of intracellular proteins in order to survive and adapt to a changing and potentially hostile environment^1^. The rapid removal of misfolded or faulty proteins through the cellular proteasome system is critical for the proper functioning of cells and organismal health^2^; however, in addition, the proteasome is also responsible for the rapid turnover of regulatory proteins^3^. Responses to external and internal stimuli must occur quickly to enable fast cellular reactions, ranging from the sensing of extracellular signals to the activation of cellular defense mechanisms^4^ and adaptive responses to a fluctuating environment. For example, the half-lives of transcription factors are short, in order to provide rapid and tightly controlled signal transmission^5,6^. Various other time-critical intracellular processes, such as progression through the cell cycle, are also regulated by protein degradation^7^.

A myriad of small modifications that target proteins for proteolysis have been described and reviewed previously^8–10^. These modifications, called degrons^8^, play key roles in regulating the degradation rate of proteins. One example is ubiquitin (Ub), a small, highly conserved 76-amino-acid polypeptide modification that is covalently attached to proteins by the cooperative action of three enzymes, Ub-activating enzyme (E1), Ub-conjugating enzyme (E2), and Ub ligase (E3), which are components of the Ub-proteasome system. By linking Ub to intracellular proteins for targeting to the proteasome, cells can directly modulate the intracellular protein concentration. Other examples of degrons include short intrinsic amino acid sequences, such as the D-element^11^, the PEST sequence^10^, unstructured initiation sites^12^, or short sequences rich in acceptor lysines^13^; they influence protein stability by promoting Ub-independent proteolysis. Degrons are widely used in fundamental research in gene-function analysis and to study the roles of essential proteins and the effects of their depletion^14,15^, in novel technologies such as the CRISPR/Cas9 system^16,17^, and for the design of scaffolds that either inhibit or activate HECT-family E3 ligases^18^. In addition, protein modifications are useful tools for studying immune or neurodegenerative disorders such as cancer^19^ or Alzheimer’s disease^20^, and for the development of new therapies for various conditions^21,22^.

In this work, we aim to develop a versatile, easy-to-use toolbox that allows researchers to precisely regulate protein expression patterns in mammalian cells by modulating target protein half-lives in a predictable manner, to provide a wide variety of protein degradation profiles. For this purpose, we first assemble a library of versatile degrons that have been tested in frequently used human and rodent cell lines. To validate this library, we fuse six well-characterized degron tags to the tetracycline (Tet)-dependent transactivator (tTA) transactivator of the established Tet gene-regulation system in order to modify and fine-tune the tTA expression dynamics. We confirm that the dynamic ranges of both Tet-ON and the Tet-OFF dose–response curves can be readily modulated without affecting the inherent properties of the Tet system, such as tight and reversible induction of gene expression. Next, we combine this library with a synthetic pulse-generator circuit designed for use in mammalian cells. By fusing the degrons to the pulse-generator elements, we obtain a series of pulse generators that afford precise control of pulse durations of downstream gene expression. Pulsatile dynamics are widely applied in synthetic biology to achieve coordinated behavior in cell populations^23^, for therapeutic purposes^24^, and in the design of artificial genetic networks such as bioelectronic interfaces^25^, computational molecular networks^26^, or synthetic devices exhibiting memory^27^.

## Results

### Selection and characterization of the degron library

**Fig. 1:**
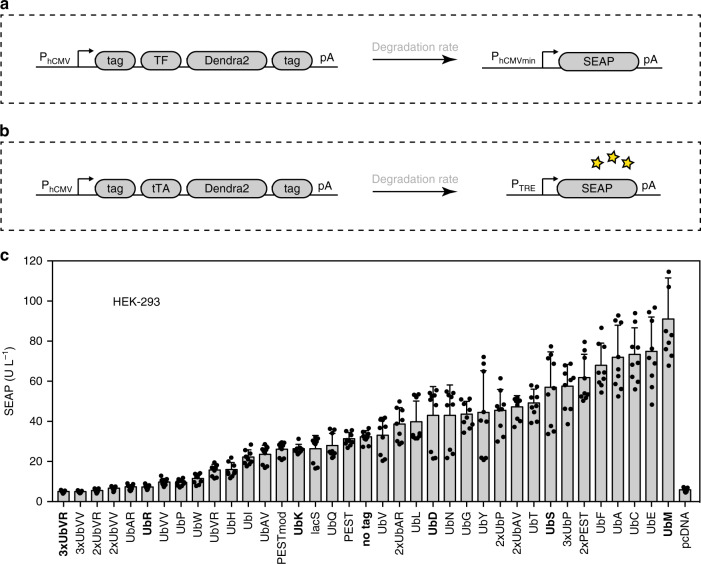
Design and set-up of the protein tag library. **a** Schematic of the constructs forming the library. The core of the constructs consists of a transcription factor (TF) fused to the photoconvertible protein Dendra2. The protein modifications (tag) affecting the degradation rate of the constructs are fused to TF-Dendra2 either at the N- or at the C- terminus. **b** To characterize the degron library, each degron was used to tag a tetracycline-dependent transactivator (tTA), which binds to its cognate Tet response element (TRE) located at the 5′- end of a minimal promoter placed in front of the human secreted embryonic alkaline phosphatase (SEAP) reporter gene (P_TRE_-*SEAP*-pA). **c** Complete library of 36 protein modifications. HEK-293 cells (3 × 10^4^) were transfected with the different constructs (P_hCMV_-tag-*tTA*-*Dendra2*-pA, containing the tetracycline-dependent transactivator tTA) and the reporter gene (P_TRE_-*SEAP*-pA). SEAP levels were profiled after 24 hours (h) in the culture supernatant. The degrons marked in bold are the six ubiquitin (Ub) fusion constructs bearing the tags 3xUbVR, UbR, UbK, UbD, UbS, and UbM, which were selected as providing representative degradation patterns. The data represent the mean ± SD (*n*  =3 independent experiments). Source data for this figure is available in the Source data file

First, we assembled a library of protein modifications that influence the in vivo half-life of proteins to which they are fused (Fig. 1a). This library contains a wide range of Ub tags, as described below, together with several Ub-independent degradation tags. The covalent attachment of Ub to cellular proteins has been shown to mediate the proteasomal degradation of both short- and long-lived proteins, as well as to participate in the removal of abnormal and denatured proteins^3^. Two major pathways target the proteins for degradation either via the N-end rule pathway or the Ub-fusion degradation (UFD) pathway (Supplementary Fig. 1a, b). The N-end rule implies that the N-terminal amino acid of a protein determines its metabolic in vivo half-life^8,28,29^. Our library contains a set of 20 such Ub tags (UbR, UbP, UbW, UbH, UbI, UbK, UbQ, UbV, UbL, UbD, UbN, UbG, UbY, UbT, UbS, UbF, UbA, UbC, UbE, UbM) with an intact C-terminal isopeptidase site (Supplementary Fig. 1a). Furthermore, the library contains another set of ten Ub tags (3xUbVR, 3xUbVV, 2xUbVR, 2xUbVV, UbAR, UbVV, UbVR, UbAV, 2xUbAR, 2xUbAV) targeting proteins to the UFD pathway^30^ (Supplementary Fig. 1b). In these cases, the Gly76 residue of Ub is mutated and the C-terminal isopeptidase site is no longer recognized by deubiquitinating enzymes. Finally, four more tags were included in the library: lacS, PEST, 2xPEST, and PESTmod. These degrons influence the half-lives of proteins either via the Ub-proteasome pathway, as described for the *Escherichia coli* Lac repressor-derived spacer^30^, or via a Ub-independent pathway^10,31^ (PEST, 2xPEST, and PESTmod) (Supplementary Fig. 1c).

To characterize the degron library, each degron was used to tag a tTA, which binds to its cognate Tet response element (TRE) located at the 5′-end of a minimal promoter placed in front of the human secreted embryonic alkaline phosphatase (SEAP) reporter gene (pMM130, P_TRE_-*SEAP*-pA) (Fig. 1b). Moreover, fusion of the photoactivable fluorescent protein Dendra2 to the tagged transcription factor tTA enabled us to measure the in vivo half-lives of the modified transcription factors and thus to determine the influence of the degrons thereon^32,33^. These constructs were expressed in human embryonic kidney cells (HEK-293) (Fig. 1c). The degradation pattern of the tTA-Dendra2 construct differed depending upon the fused degron, providing an 18-fold range of SEAP expression, ranging from 5 U/L SEAP (3xUbVR) to 91 U/L SEAP (UbM) after 24 h in culture. An untagged construct (pCHX17, P_hCMV_-*tTA-Dendra2*-pA) was inserted to benchmark the expression level of the fusion protein without any degron. The different amounts of SEAP reporter in the cell culture supernatant were not due to cytotoxic effects of the degrons, as their expression in HEK-293 cells did not affect the cell viability (Supplementary Fig. 2a). Six representative Ub-fusion tags (Fig. 1c, Supplementary Fig. 2a) were selected for further study: 3xUbVR, UbR, UbK, UbD, UbS, and UbM. The use of these six degrons fused to tTA and Dendra2 in two other widely used human cell lines (human mesenchymal stem cells (hMSCs) (Supplementary Fig. 2b) and human cervical carcinoma cells (HeLa) (Supplementary Fig. 2c)), and one rodent cell line (Chinese hamster ovary (CHO-K1) (Supplementary Fig. 2d)) showed that the degradation patterns of the constructs were reproducible, indicating a broad applicability of this methodology.

To rule out the possibility that the different levels of SEAP expression were due to differences in the DNA transfection efficiency, the six Ub degrons fused to tTA were linked to an mCherry fluorescent protein via a self-cleaving P2A peptide^34^ (Supplementary Fig. 3a). Fluorescence microscopy revealed an even distribution of mCherry fluorescence in the cell population, whereas the expression of Dendra2 was highly dependent on the nature of the fused degradation tag (Supplementary Fig. 3b). Thus, the variations in the Dendra2 fluorescence pattern enabled us to visualize the influence of the degrons on the expression of the photoactivable protein Dendra2 (Supplementary Fig. 3b). Moreover, SEAP expression levels were assessed in parallel (Supplementary Fig. 3c) and showed the same behavior as the Dendra2 fluorescence in these cell populations (Supplementary Fig. 3d).

The different degradation patterns were confirmed to be stable (Supplementary Fig. 4a). Furthermore, the six degrons fused to tTA and Dendra2 were cloned downstream of standard promoters to confirm their broad applicability in various biological contexts (Supplementary Fig. 4b). Besides the human cytomegalovirus immediate-early promoter (P_hCMV_) shown in Fig. 1c, we tested the simian virus 40 enhancer and early promoter (P_SV40_) (Supplementary Fig. 4c), the human elongation factor-1 alpha promoter (P_hEF1α_) (Supplementary Fig. 4d), and the murine phosphoglycerate kinase 1 promoter (P_PGK_) (Supplementary Fig. 4e). The induction levels of the different promoters were similar, supporting the transcriptional independence of the tags. A control experiment using proteasome inhibitor AdaAhx3L3VS^35^ was run with HEK-293 cells transfected with the six degrons fused to tTA and Dendra2, to investigate whether the inhibition of proteasome activity leads to an accumulation of the Ub-tagged Dendra2 constructs. Addition of AdaAhx3L3VS (50 μM) 10 h prior to fluorescence microscopy (Supplementary Fig. 5a) effectively prevented the degradation of the tagged constructs (Supplementary Fig. 5b). The incubation with AdaAhx3L3VS resulted in an 8-fold increase of the unstable 3xUbVR-tagged construct, whereas 1.8-fold induction was observed for the stable UbM-tTA-Dendra2 fusion construct (Supplementary Fig. 5b).

Finally, the six selected tags were fused to the widely used *Streptococcus pyogenes* dead Cas9 protein (dCas9) (Supplementary Fig. 6a, b) and to its synergistic activation MCP-VPR construct^36^ (Supplementary Fig. 6c, d), to confirm the universality of the degrons for protein expression control. The nuclease dCas9 is a large protein that targets specific regions of DNA in the presence of gRNA, whereas the RNA-binding coat protein (MCP) supplied with a VP64-p65-Rta (VPR) tripartite activator targets the gRNA itself, forming a protein complex with a transcription factor-like action. The addition of the stabilizing UbM tag to dCas9 resulted in a 65-fold increase of SEAP expression, compared with that observed when the unstable 3xUbVR-dCas9 construct was used (Supplementary Fig. 6b). Moreover, the induction of SEAP expression was 130-fold greater for the UbM-tagged MCP-VPR, compared with the 3xUbVR-tagged MCP-VPR construct (Supplementary Fig. 6d).

### Tuning dose–response dynamics of the Tet system by degrons

**Fig. 2:**
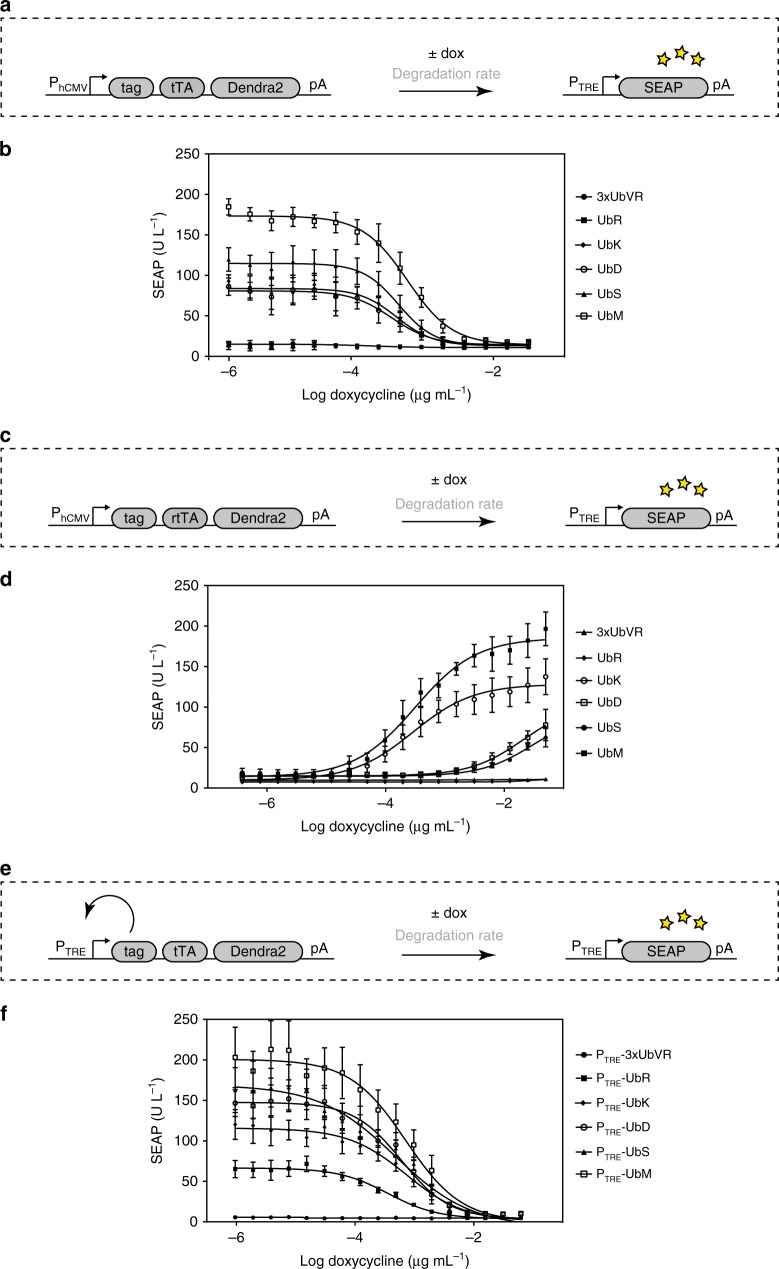
Tunability of the dose–response curves with six selected protein tags. **a** Schematic of the six selected tags (3xUbVR, UbR, UbK, UbD, UbS, UbM) fused to tTA-Dendra2 and exposed to increasing concentrations of doxycycline (dox). **b** HEK-293 cells (3 × 10^4^) were transfected with the different constructs (P_hCMV_-tag-*tTA*-*Dendra2*-pA) and the reporter gene (P_TRE_-*SEAP*-pA). SEAP levels in the culture supernatant were profiled after 24 h. **c** Schematic of the six selected tags (3xUbVR, UbR, UbK, UbD, UbS, UbM) fused to rtTA-Dendra2 and exposed to increasing concentrations of dox. **d** HEK-293 cells (3 × 10^4^) were transfected with the different constructs (P_hCMV_-tag-*rtTA*-*Dendra2*-pA) and the reporter gene (P_TRE_-*SEAP*-pA). SEAP levels in the culture supernatant were profiled after 24 h. **e** Schematic of the six selected tags (3xUbVR, UbR, UbK, UbD, UbS, UbM) fused to the autoactivating P_TRE_-tag-*tTA*-*Dendra2*-pA construct and exposed to increasing concentrations of dox. **f** HEK-293 cells (3 × 10^4^) were transfected with the different constructs (P_TRE_-tag-*tTA*-*Dendra2*-pA) and the reporter gene (P_TRE_-*SEAP*-pA). SEAP levels in the culture supernatant were profiled after 24 h. The data represent the mean ± SD (*n* = 3 independent experiments) measured in triplicate. Source data for this figure is available in the Source data file

To validate that our methodology allows to fine-tune the response of synthetic networks to environmental stimuli, we examined the effect of changing protein half-life on the dynamic range of the dose–response curves in the well-established Tet system to see whether the response to an input signal could be fine-tuned. With a fixed SEAP reporter (pMM130, P_TRE_-*SEAP*-pA), the concentration of the Tet derivative doxycycline was varied and the dose-dependent inductions of 3xUbVR, UbR, UbK, UbD, UbS, and UbM fused to tTA and Dendra2 were examined (Fig. 2a). Using the Tet-OFF system (Fig. 2a, b), we obtained a 15-fold change in the dose–response curves of the six tTA-Dendra2 fusion constructs, covering the range from 12 U/L (3xUbVR) to 184 U/L (UbM). Increase of the repressive doxycycline input resulted in steady declines (Fig. 2b) to minima ranging between 12 U/L (3xUbVR) and 17 U/L (UbM).

We further introduced a reverse tTA (rtTA) instead of tTA (Fig. 2c, d). Using rtTA, SEAP levels ranged from 9 U/L (3xUbVR) to 20 U/L (UbM) in the absence of doxycycline. As the inducer concentration was raised, SEAP production increased to the range of 10 U/L (3xUbVR) to 210 U/L (UbM). The six degrons showed significantly different dose–response curves, confirming that their characteristic effects are retained in the transactivator-based Tet system.

Next, we examined self-activating constructs in which the P_hCMV_ promoter was replaced with a TRE promoter (P_TRE_*-tag-Dendra2*-pA) (Fig. 2e). Using the SEAP reporter (pMM130, P_TRE_-*SEAP*-pA) in parallel, the autoactivated dose–response curves could be assessed in the same manner as used for the original P_hCMV_ promoter constructs. As in the Tet-OFF system, the curves started from clearly distinct maximal rates ranging from 5 U/L (3xUbVR) to 202 U/L (UbM) and fell to 3 U/L (3xUbVR) to 7 U/L (UbM), reflecting the different degradation patterns of the proteins (Fig. 2f). The different induction behaviors were not a result of different expression strengths (e.g., due to different promoters), but were rather caused by the altered dose–response curves. Together, our results support the idea that our modular degron library can modulate the protein expression profile and therefore the behavior of complex biologic systems.

### Measuring the half-lives of the degron-tagged tTA

**Fig. 3:**
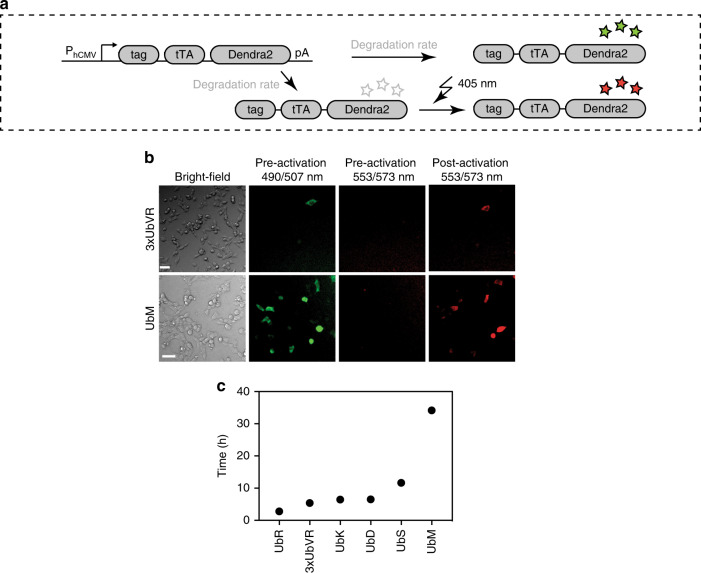
Determination of the protein half-lives of the six selected constructs. **a** Before photoconversion, the chromophore of Dendra2 exists in an equilibrium between neutral (nonfluorescent) and anionic (green fluorescent) states. Excitation of the neutral chromophore with light at 405 nm results in photoconversion to a red fluorescent state. **b** Fluorescence microscopy images of cells transfected with P_hCMV_-3x*UbVR*-*tTA*-*Dendra2*-pA and P_hCMV_-*UbM*-*tTA*-*Dendra2*-pA before and after photoconversion with 405 nm light. The image intensities were all stretched by the same factor, while maintaining the same intensity ratio for better visibility. White scale bars correspond to 50 µm. **c** HEK-293 cells (3 × 10^4^) were transfected with the six selected tags (3xUbVR, UbR, UbK, UbD, UbS, UbM) and the half-lives of the constructs were determined by time-lapse microscopy. Photoconversion was conducted after 24 h with blue light and fluorescence images were recorded every 10 min. The data represent the mean (*n* = 3 independent experiments) measured in triplicate. Source data for this figure is available in the Source data file

Knowledge of protein half-lives is an important prerequisite for the synthetic construction and functional analysis of dynamic biological systems^37^. Here, in order to assess the half-lives of the tagged tTA transactivators, we fused them to Dendra2 (Fig. 3a), a green-to-red photoconvertible protein used for the photolabeling and tracking of proteins^38^. The photoconversion conditions for HEK-293 cells were optimized, and to exclude the influence of cytotoxicity of the photoconverting laser light, cell viability was assessed after 24 h (Supplementary Fig. 7a).

Half-lives were determined by transfecting the six selected constructs (3xUbVR, UbR, UbK, UbD, UbS, and UbM) into HEK-293 cells and photoconverting the proteins 24 h after transfection. Fluorescence microscopic images pre- and post-irradiation at 405 nm of HEK-293 cells transfected with the 3xUbVR- and the UbM-tagged constructs are shown in Fig. 3b. Time-lapse fluorescence microscopy was used to follow protein degradation in the cells by measuring the fluorescence intensity decrease of photoconverted Dendra2 (Fig. 3c). Mean fluorescence intensity was measured every 10 min for at least 16 h, to obtain high time resolution. The half-lives of the degron-tagged tTA transactivators were in the order 2.8 h < 5.4 h < 6.4 h < 6.5 h < 11.7 h < 34.1 h for the UbR, 3x UbVR, UbK, UbD, UbS, and UbM constructs, respectively (Fig. 3c and Supplementary Fig. 4b).

Finally, to establish a detailed understanding of how the degrons impact the expression levels, we constructed a mathematical model that incorporates all important molecular processes of the system. The mathematical model predicted that the dependency of the SEAP concentrations, 24 h after induction, on the (degron dependent) half-lives of the proteins should approximately follow a Hill curve with a Hill coefficient of two (Supplementary Notes). When we plotted the experimentally measured SEAP concentrations as a function of the half-lives (Supplementary Fig. 8), we indeed found a close fit of our experimental data to this mathematical prediction, which demonstrates that our two measures of protein stability result in consistent experimental insights on the dynamic impact of the different degrons.

### Engineering a tunable pulse-generator circuit

In order to guarantee maximum versatility of our methodology for the control of protein expression in the context of complex synthetic networks, we integrated our degron library with a synthetic pulse generator. Pulse generators are adaptive synthetic modules that generate transient cellular responses (or pulses) to sustained external or internal triggers (e.g., small molecules), which can activate certain cellular key elements for a defined period (pulse length), which may range from milliseconds^39^ to several hours^40^. Examples of naturally evolved adaptive networks that realize pulse generators include chemotaxis^41,42^ and stress response^43^ in bacteria, and several processes involved in embryonal development^44^, cell cycle regulation^45^, and responses to extracellular inputs^46–49^ in mammalian cells. Also in the context of synthetic biology, the design of many artificial networks requires defined pulsatile dynamics, e.g., in the set-up of oscillators^50^, circadian clocks^51^, toggle switches^52^ or sensor–effector devices^53^. Therefore, the creation of precisely defined pulse-generating circuits is indispensable for engineering synthetic and dynamic gene networks.

**Fig. 4:**
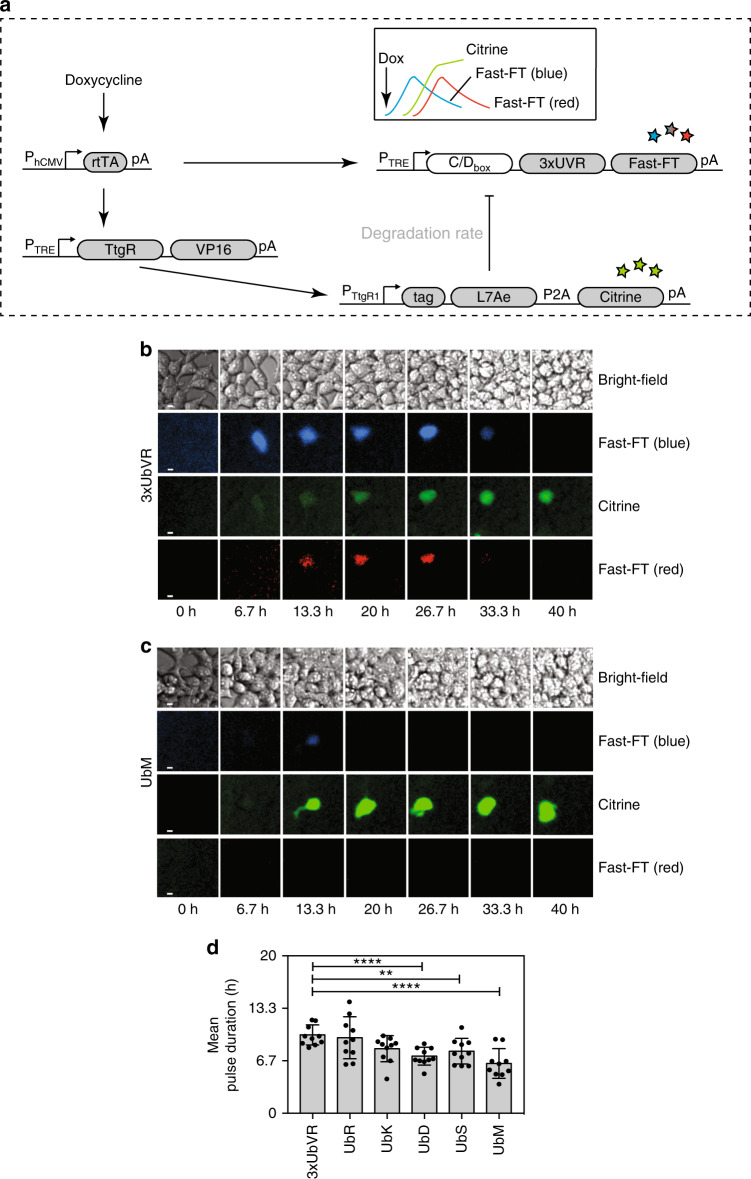
Pulse generator. **a** Schematic of the pulse-generator components. Upon addition of the trigger (doxycycline (dox)), the reverse tetracycline-dependent transactivator trigger-sensing module (rtTA, sensing doxycycline) drives the expression of the fast fluorescent timer (Fast-FT) reporter protein and the time-delay TtgR-VP16 element. TtgR-VP16 binds in turn to its cognate operator sequence to drive the expression of the negative-feedback element L7Ae (tagged with the six selected degrons 3xUbVR, UbR, UbK, UbD, UbS, UbM). To monitor the induction of the L7Ae repressor element, a fluorescent protein (Citrine) is attached via a self-cleaving P2A peptide. L7Ae binds to the C/D_box_ of a 3xUbVR-tagged Fast-FT mRNA and represses its translation to a fluorescent protein. The in vivo half-life of L7Ae is altered by the degrons and influences the repression strength of L7Ae. **b** Time-lapse bright-field and fluorescence microscopy images of HEK-293 cells transfected with the reporter gene and the different negative-feedback elements fused to the 3xUbVR tag. The images were recorded every 20 min for 40 h and show the same cells at the same time points imaged with different wavelengths. The image intensities were all stretched by the same factor while maintaining the same intensity ratio for better visibility. White scale bars correspond to 10 µm. **c** Time-lapse bright-field and fluorescence microscopy images of HEK-293 cells transfected with the reporter gene and the different negative-feedback elements fused to the UbM tag. The images were recorded every 20 min for 40 h and show the same cells at the same time points imaged with different wavelengths. The image intensities were all stretched by the same factor while maintaining the same intensity ratio for better visibility. White scale bars correspond to 10 µm. **d** Mean pulse durations of the different pulse generators. Data represent the mean ± SD (*n* = 5) measured in duplicate. **P < 0.005, ****P < 0.0001, Student’s *t-*test. Source data for this figure is available in the Source data file

The design of our pulse generator (Fig. 4a) includes (i) a trigger-sensing module (rtTA, sensing doxycycline), (ii) a time-delay element (TtgR, phloretin-dependent transactivator), (iii) a negative-feedback loop (the RNA-binding protein L7Ae), and a dynamic fast fluorescent timer (Fast-FT) reporter (3xUbVR-tagged Fast-FT)^54^. Upon the addition of doxycycline, rtTA binds to its cognate operator site and enables expression of the reporter gene Fast-FT (Fig. 4a) and of TtgR in a time-delayed fashion. TtgR itself binds to its operator site and drives the expression of 3xUbVR, UbR, UbK, UbD, UbS, or UbM-tagged L7Ae, which in turn binds to the C/D_box_ of the reporter gene mRNA, thereby repressing its translation to a fluorescent protein.

Our precise characterization of the six degrons described above (3xUbVR, UbR, UbK, UbD, UbS, and UbM) enabled us the design a set of synthetic pulse generators with variable pulse lengths. The pulse-generator elements were cloned onto two plasmids (pCHX301, P_TRE_-*TtgR*-*VP16*-pA:P_hCMV_-*rtTA*-pA and pCHX300, P_TtgR1_-3x*UbVR*-*L7Ae*-P2A-*Citrine*-pA:P_TRE_-*C/D*_*box*_-3x*UbVR*-*Fast-FT*-pA, or pCHX308, P_TtgR1_-*UbM*-*L7Ae*-P2A-*Citrine*-pA:P_TRE_-*C/D*_*box*_-3x*UbVR*-*Fast-FT*-pA, pCHX309, P_TtgR1_-*UbS*-*L7Ae*-P2A-*Citrine*-pA:P_TRE_-*C/D*_*box*_-3x*UbVR*-*Fast-FT*-pA, pCHX310, P_TtgR1_-*UbK*-*L7Ae*-P2A-*Citrine*-pA:P_TRE_-*C/D*_*box*_-3x*UbVR*-*Fast-FT*-pA, pCHX311, P_TtgR1_-*UbR*-*L7Ae*-P2A-*Citrine*-pA:P_TRE_-*C/D*_*box*_-3x*UbVR*-*Fast-FT*-pA, pCHX312, P_TtgR1_-*UbD*-*L7Ae*-P2A-*Citrine*-pA:P_TRE_-*C/D*_*box*_-3x*UbVR*-*Fast-FT*-pA) to guarantee the co-localization of all the elements necessary for the generation of a fluorescent pulse within the same cell. The pulse-generator elements were transfected into HEK-293 cells and the resulting pulse patterns were observed by light microscopy. Citrine was attached to L7Ae via a self-cleaving peptide P2A, to monitor the induction of the repressing L7Ae element (Fig. 4b, Supplementary Fig. 9a, b, Supplementary Movies 1 and 2). By applying the half-life-modulating tags to the negative-feedback element P_TtgR1_-tag-*L7Ae*-P2A-*Citrine*-pA of the pulse generator, we could establish a set of expression pulses with mean durations of 10 h (3xUbVR), 9.6 h (UbR), 8.2 h (UbK), 7.3 h (UbD), 7.9 h (UbS), and 6.3 h (UbM) (Fig. 4d). Modulation of the protein stability of L7Ae and hence the negative-feedback element of the pulse generator could provide shorter pulse lengths for more stable protein elements such as the UbM-tagged L7Ae protein or longer pulse lengths for the unstable 3xUbVR-tagged L7Ae element.

As a control, the trigger module rtTA alone failed to generate pulsatile dynamic behavior, confirming the suitability of both the trigger module and the reporter Fast-FT (Supplementary Fig. 10a, b, Supplementary Movie 3). To show that every element is necessary for the correct functioning of the pulsing circuit, we removed individual elements of the pulse generator (such as P_TRE_-*TtgR*-*VP16*-pA) (Supplementary Fig. 11a, b, Supplementary Movie 4) and demonstrated that the negative-feedback module (P_TtgR1_-tag*-L7Ae*-pA) alone does not repress the translation of the Fast-FT reporter mRNA to a fluorescent protein, while the constitutive expression of the negative-feedback module (pCHX255, P_hCMV_-3x*UbVR*-*L7Ae*-pA) fully represses the expression of the Fast-FT reporter protein (Supplementary Fig. 12a, b). Supplementary Fig. 13a, b shows that the time-delay element TtgR is necessary for the maturation and accumulation of the Fast-FT reporter protein within the cells. Without this element, L7Ae represses the translation of the reporter protein and the cells fail to produce detectable amounts of Fast-FT.

## Discussion

Libraries of functional elements play an important role in fundamental research in various fields of biology, ranging from gene therapy and biotechnology to systems and synthetic biology. For example, libraries of synthetic promoters affording strong activation of transcription^55^, exhibiting highly predictable activities^56^, or designed for specific types of mammalian cells^57^ have been previously reported and are frequently employed. Likewise, large prokaryotic riboregulators^58^, RNA-mediated transcriptional repressors^59^, and allosteric signaling switch^60^ libraries have been established. In this study, we aimed to develop a versatile, easy-to-handle toolbox that would enable us to precisely regulate protein expression profiles in mammalian cells; such methodology would have many applications, including studies on the functional roles of essential proteins, fine-tuning gene-expression systems, and adding complexity in the design of biological systems in the field of synthetic biology. For this purpose, we first assembled a versatile degron library and assessed its effects on protein stability by fusing the degrons to transcription factors that induce the expression of a synthetic reporter gene (SEAP). Our results confirm that the degrons do not affect cellular viability and allow for the precise tuning of protein expression without the need to exchange promoters or vary the amount of transfected DNA. The differences in the SEAP expression vary from cell line to cell line without changing the rank order of SEAP expression levels (Supplementary Fig. 2b, c, d). Such differences are commonly observed^61,62^; they are due to the following: (i) differences in gene and protein expression levels dependent on the species (e.g., human, hamster) and tissue (e.g., liver, ovary, and kidney); (ii) transfection efficacies that vary from cell line to cell line; and (iii) species variations in the degradation machinery (e.g., in E3 ligases).

We further confirmed that these degrons could modulate the well-established Tet transcriptional activation system, which is a tightly controlled, reversible gene-expression system with low background and high induction rates. The transactivator could be fused to the degrons without affecting its binding properties to its cognate operator site, the activation or repression by doxycycline was not impaired, and gene induction showed no leakiness, while the induction rates remained high. The ability of these degrons to modulate the dynamic range of the tTA dose–responses without altering the inherent gene-regulation properties of the system should greatly increase the applicability of Tet-based transcriptional activation systems, which are already widely used in common mouse models^63^, for large-scale production of therapeutics^64^, and in synthetic biology^52,65^.

In order to guarantee maximum flexibility in the control of protein expression profiles, we extensively tested our library by linking the individual degrons to the genetic components constituting a synthetic pulse-generating circuit. Robust genetic circuits such as pulse-generators are developed using standardized and modularized biological parts^66^, and are fundamental for the establishment of sophisticated systems-level circuitry. Such circuits support the engineering of novel complex and multicellular systems that can sense and communicate^67^, and are essential for the development of personalized medicine and biomedical science. The pulsatile behavior of our circuit is an important requirement for numerous synthetic networks, including Boolean processing logic devices and closed-loop control capacity devices^25,26,67^, complex sensor–effector devices (so-called prosthetic gene networks)^68,69^ for personalized medicine, and biosensors for reporting the presence of target analytes^62^. Furthermore, pulse-generating circuits have been proposed to achieve coordinated behavior in *E. coli*^23^. However, the complexity of mammalian cells and the noise that originates from cell cycle progression, apoptosis, internal and external cellular signaling, and environmental fluctuations are major challenges to the construction of robust and predictable systems^70^. The design of our circuit is based on two orthogonal elements (Tet and TtgR) and a powerful translation repressor (L7Ae). The orthogonality of the Tet and TtgR transactivators decreases the risk of cellular crosstalk^71^, whereas the small number of genetic elements constituting the circuit favors robustness. By linking degrons to our pulse-generating circuit components, we could tune the pulse duration of protein expression, opening up exciting opportunities for the design and optimization of new genetic circuits. For example, single-gene networks have been proposed to buffer protein synthesis rates in mammalian cells against perturbations; they provide stable steady-state expression levels, but also exhibit a pulse-like behavior by showing initial and transient expression peaks^72^. We found that we could achieve near-perfect adaptation of our pulse generator by using the most destabilizing degron tag (3xUbVR) in our library: this degron decreased the dependency of the steady-state expression levels on the level of the environmental activators to minimal levels. Thus, the short half-life of the reporter protein enables us to obtain a very well-defined pulse of fluorescent protein expression that could not be realized with the untagged reporter protein. The pulse length spread observed in Fig. 4d is the result of the inherent biological variation, which is due to a variation of genetic components and different cellular regulatory elements^73^.

Our circuit is modular and might be easily adapted to function via the signaling of a receptor. Notably, the fine-tuning of the pulsatile dynamics is simple, predictable, and does not affect the functionality of the circuit elements. We believe the ability of our modular degron library/synthetic pulse-generating circuit to precisely regulate protein expression patterns in mammalian cells will open up many new possibilities in the fields of biology and medicine.

## Methods

### Components of the protein tag library

Comprehensive design and construction details for all expression vectors are provided in Supplementary Table 1. The degrons and the proteins used in this study are inserted via standardized restriction sites, which rely on compatible ends (*Spe*I and *Nhe*I) and enable the repeated fusion of multiple degrons. The resulting amino-acid linker sequence upon fusion is Ala-Ser-Ala. The six characterized constructs are 3xUbVR (pCHX50, P_hCMV_-3x*UbVR-tTA-Dendra2*-pA; Genbank MK448012 [https://www.ncbi.nlm.nih.gov/nuccore/MK448012]), UbR (pCHX91, P_hCMV_-*UbR*-*tTA*-*Dendra2*-pA; Genbank MK453494 [https://www.ncbi.nlm.nih.gov/nuccore/MK453494]), UbK (pCHX185, P_hCMV_-*UbK*-*tTA*-*Dendra2*-pA; Genbank MK453498 [https://www.ncbi.nlm.nih.gov/nuccore/MK453498]), UbD (pCHX178, P_hCMV_-*UbD*-*tTA*-*Dendra2*-pA; Genbank MK453496 [https://www.ncbi.nlm.nih.gov/nuccore/MK453496]), UbS (pCHX130, P_hCMV_-*UbS*-*tTA*-*Dendra2*-pA; Genbank MK453495 [https://www.ncbi.nlm.nih.gov/nuccore/MK453495]), and UbM (pCHX181, P_hCMV_-*UbM*-*tTA*-*Dendra2*-pA; Genbank MK453497 [https://www.ncbi.nlm.nih.gov/nuccore/MK453497]).

### Cell culture and transfection

Human embryonic kidney cells (HEK-293, ATCC: CRL-11268), human cervical carcinoma cells (HeLa, ATCC: CCL-2), and immortalized human mesenchymal stem cells (hMSCtert; hMSC-TERT^74^) were cultured in Dulbecco’s modified Eagle’s medium (DMEM; catalog number 52100-039; Life Technologies, Carlsbad, CA, USA) supplemented with 10% fetal calf serum (FCS; catalog number 2-01F10-I; lot number PE01026P; BioConcept, Allschwil, Switzerland) and a 1% penicillin/streptomycin solution (catalog number P4333; Sigma-Aldrich, Munich, Germany). Chinese hamster ovary cells (CHO-K1, ATCC: CCL-61) were cultivated in ChoMaster HTS (catalog number CHTS-8; Cell Culture Technologies, Gravesano, Switzerland) supplemented with 5% FCS and a 1% penicillin/streptomycin solution. All the cell types were cultured at 37 °C in a humidified atmosphere containing 5% CO_2_. The cell numbers and viability were quantified using an electric field multichannel cell-counting device (CASY Cell Counter and Analyzer Model TT; Roche Diagnostics GmbH, Rotkreuz, Switzerland). The cells were transfected using an optimized PEI transfection protocol. The cells were seeded 16 h before transfection at 3 × 10^4^ per well of a 48-well plate. The transfection was performed by incubating 0.2 µg plasmid DNA and 1 µL PEI (1 mg/mL stock solution, catalog number 24765-2; Polyethylenimine Max, Polysciences, Inc., Warrington, PA, USA) per well in FCS-free DMEM for 15 min at 22 °C and then adding 100 µL of the transfection mix dropwise to the cells. For SEAP output, 0.02 µg of pMM130 (P_TRE_-*SEAP*-pA) and 0.001 µg of tagged construct (P_hCMV_-tag-*tTA*-*Dendra2*-tag-pA) were used. For the autoactivated constructs with SEAP output, 0.02 µg of pMM130 (P_TRE_-*SEAP*-pA) and 0.02 µg of tagged construct (P_TRE_-tag-*tTA*-*Dendra2*-pA) were used. For the fluorescence microscopic imaging, 0.3 µg of the tagged constructs (P_hCMV_-tag-*tTA*-*Dendra2*-pA) was used. For the pulse generator, 0.0025 µg of pCHX301 (P_TRE_-*TtgR*-*VP16*-pA:P_hCMV_-*rtTA*-pA), 0.0175 µg of pMM591 (P_hCMV_-*rtTA*-pA), and 0.02 µg of the tagged L7Ae and reporter gene containing plasmid (P_TtgR1_-tag-*L7Ae*-P2A-*Citrine*-pA:P_TRE_-C/D_box_-3x*UbVR*-*Fast-FT*-pA) were used. For the dCas9 and MCP experiments, 0.002 µg of the tagged constructs ((P_hCMV_-tag-*dCas9*-*Dendra2*-pA) or (P_hCMV_-tag-MCP-VPR-*Dendra2*-pA)) and 0.2 µg of dCas9 (P_hCMV_-*dCas9*-*Dendra2*-pA) or MCP (P_hCMV_-MCP-VPR-*Dendra2*-pA), gRNA (P_hU6_-sgRNA_hINS_), and SEAP reporter gene (P_Ins_-*SEAP*-pA) were used. pUC57 was used to adjust the DNA amount to 1.6 µg in total. HEK-293 cells transfected with pulse-generator elements were programmed with doxycycline (1.6 ng/mL, stock solution: 1.5 mg/mL in ethanol; catalog number D9891, Sigma-Aldrich, Munich, Germany). After 6 h, the medium was replaced with standard cultivation medium. Medium supplemented with the indicated amount of doxycycline was added 24 h after transfection. Unless stated otherwise, the transgene expression was profiled or fluorescence microscopy imaging was conducted after 24 h.

### Analytical assays

*Resazurin cell viability assay*: Metabolic activity and proliferation of living cells were quantified directly in the cell culture wells by addition of resazurin (40 μg/mL resazurin sodium salt stock solution: 1 mg/mL in phosphate-buffered saline, catalog number R7017, Sigma-Aldrich, Munich, Germany) to the cell culture plate. Fluorescence measurements (excitation at 570 nm, emission at 590 nm) were performed after a 3 h incubation period at 37 °C in a humidified atmosphere of 5% CO_2_ in air, using a GeniosPro multi-well reader (Tecan, Maennedorf, Switzerland). *SEAP levels*: Human placental secreted alkaline phosphatase in the cell culture supernatant^80^ was quantified. In brief, 100 µL of the cell culture supernatant to a 96-well multiplate and heat-inactivating for 30 min at 65 °C. Subsequently, 80 µL of the supernatant is transferred per well of a 96-well plate containing 100 µL 2× SEAP assay buffer (20 mM homoarginine, 1 mM MgCl_2_, 21% (v/v) diethanolamine, pH 9.8). After the addition of 20 µL of 120 mM *para*-nitrophenyl phosphate (pNPP disodium salt, hexahydrate, catalog number AC128860100; Acros Organics BVBA, Geel, Belgium) diluted in 1× SEAP assay buffer, the time-dependent increase in light absorbance is measured at 405 nm for 30 min using a GeniosPro multi-well reader (Tecan, Maennedorf, Switzerland).

### Fluorescence imaging

Fluorescence microscopy was performed with an inverted fluorescence microscope (Nikon Ti-E; Nikon) equipped with an incubation chamber, an Orca Flash-4 digital camera (Hamamatsu), a pE-100-LED (CoolLED) as the transmission light source, a Spectra X (Lumencor) as the fluorescent light source, and a 10× objective (Plan Apo λ; numerical aperture, 0.45; DIC N1; working distance, 4). We collected bright-field images (40% intensity; 10 ms exposure), Dendra2 fluorescence images (before activation) (excitation, 490 nm; intensity, 10%; exposure, 200 ms; GFP ET filter, dichroic 495 nm; emission, 525/50 nm); Dendra2 (after activation) and mCherry fluorescence images (excitation, 553 nm; intensity, 40%; exposure, 500 ms; CY3 HC, dichroic 562 nm; emission, 593/40 nm); Citrine fluorescence images (excitation, 470 nm; intensity, 15%; exposure, 200 ms; YFP ET filter, dichroic 520 nm; emission, 543/22 nm); Fast-FT (blue variant) fluorescence images (excitation, 402 nm; intensity, 15%; exposure, 200 ms; DAPI HC, dichroic 416 nm; emission, 460/50 nm), and Fast-FT (red variant) fluorescence images (excitation, 553 nm; intensity, 40%; exposure, 500 ms, CY5 HC, dichroic 660 nm; emission, 700/75). A binning of 2 × 2 was used. Image analysis was performed with Imaris (Bitplane).

### Reporting summary

Further information on research design is available in the Nature Research Reporting Summary linked to this article.

## Supplementary information

Movie 1

Movie 2

Movie 3

Movie 4

Description of Additional Supplementary Files

Supplementary information

Reporting Summary

## Data Availability

The six previously characterized constructs are available from Genbank under the following accession codes: MK448012, MK453494, MK453498, MK453496, MK453495, and MK453497. Source data for Figs. 1, 2, 3, 4 and Supplementary Figs. 2, 3, 4, 5, 6, 7, 8, 9 are available in the Source Data file. All other data and materials are available upon request.

## Source data

Source Data
