# Author Correction: A modular degron library for synthetic circuits in mammalian cells

**DOI:** 10.1038/s41467-023-36111-0

**Published:** 2023-01-24

**Authors:** Hélène Chassin, Marius Müller, Marcel Tigges, Leo Scheller, Moritz Lang, Martin Fussenegger

**Affiliations:** 1 Department of Biosystems Science and Engineering, ETH Zürich, Mattenstrasse 26, CH-4058 Basel, Switzerland; 2 Cilag AG, Hochstrasse 201, CH-8200 Schaffhausen, Switzerland; 3 Institute of Science and Technology Austria, A-3400 Klosterneuburg, Austria; 4 Faculty of Science, University of Basel, Mattenstrasse 26, CH-4058 Basel, Switzerland

Correction to: *Nature Communications* 10.1038/s41467-019-09974-5, published online 01 May 2019

The original version of the Supplementary Information associated with this Article was missing Supplementary Table 1. The HTML has been updated to include a corrected version of the Supplementary Information.

