# Supplementary material for: A modular degron library for synthetic circuits in mammalian cells
Source: Nat Commun. 2019 May 1;10:2013. doi: 10.1038/s41467-019-09974-5 (PMC6494899; doi:10.1038/s41467-019-09974-5)
Supplement: Supplementary file 5 — Description of Additional Supplementary Files [file 41467_2019_9974_MOESM5_ESM.docx]

**Movies**

**Supplementary Movie 1**

Representative movie of time-lapse bright field and fluorescence microscopy images of 3 x 10^4^ HEK-293 cells transfected with pCHX301 (P_TRE_-TtgR-VP16-pA:P_hCMV_-rtTA-pA) and the 3xUbVR-tagged L7Ae containing plasmid pCHX300 (P_TtgR1_-UbVR-UbVR-UbVR-L7Ae-P2A-Citrine-pA:P_TRE_-C/D_box_-UbVR-UbVR-UbVR-Fast-FT-pA) upon addition of doxycycline. The images were recorded every 20 min during 40 h, 20x magnification.

**Supplementary Movie 2**

Representative movie of time-lapse bright field and fluorescence microscopy images of 3 x 10^4^ HEK-293 cells transfected with pCHX301 (P_TRE_-TtgR-VP16-pA:P_hCMV_-rtTA-pA) and the UbM-tagged L7Ae containing plasmid pCHX308 (P_TtgR1_-UbM-L7Ae-P2A-Citrine-pA:P_TRE_-C/D_box_-UbVR-UbVR-UbVR-Fast-FT-pA) upon addition of doxycycline. The images were recorded every 20 min during 40 h, 20x magnification.

**Supplementary Movie 3**

Representative movie of time-lapse bright field and fluorescence microscopy images of 3 x 10^4^ HEK-293 cells transfected with rtTA (pMM591, P_hCMV_-rtTA-pA) and the 3xUbVR-tagged Fast-FT reporter (pCHX273, P_TRE_-C/D_box_-UbVR-UbVR-UbVR-Fast-FT-pA) upon addition of doxycycline. The images were recorded every 20 min during 40 h, 20x magnification.

**Supplementary Movie 4**

Representative movie of time-lapse bright field and fluorescence microscopy images of 3 x 10^4^ HEK-293 cells transfected with rtTA (pMM591, P_hCMV_-rtTA-pA), the 3xUbVR-tagged Fast-FT reporter (pCHX273, P_TRE_-C/D_box_-UbVR-UbVR-UbVR-Fast-FT-pA), and the L7Ae repressor element (pCHX293, P_TtgR1_-UbVR-UbVR-UbVR-L7Ae-pA) upon addition of doxycycline. The images were recorded every 20 min during 40 h, 20x magnification.
