# Supplementary material for: A modular degron library for synthetic circuits in mammalian cells
Source: Nat Commun. 2019 May 1;10:2013. doi: 10.1038/s41467-019-09974-5 (PMC6494899; doi:10.1038/s41467-019-09974-5)
Supplement: Supplementary file 6 — Supplementary information [file 41467_2019_9974_MOESM6_ESM.pdf]

Supplementary Information

A modular degron library for synthetic circuits in mammalian cells

Chassin et al.

Supplementary Information for

A modular degron library for synthetic circuits in mammalian cells

Hélène Chassin[1], Marius Müller[2], Marcel Tigges[2], Leo Scheller[1], Moritz Lang[3], and Martin Fussenegger[1,4,]*

[1]Department of Biosystems Science and Engineering, ETH Zürich, Mattenstrasse 26, CH-4058 Basel, Switzerland
[2]Cilag AG, Hochstrasse 201, CH-8200 Schaffhausen, Switzerland
[3]Institute of Science and Technology Austria, A-3400 Klosterneuburg, Austria
[4]Faculty of Science, University of Basel, Mattenstrasse 26, CH-4058 Basel, Switzerland.
Corresponding author: Martin Fussenegger, PhD, Department of Biosystems Science and Engineering, ETH Zürich, Mattenstrasse 26, CH-4058 Basel, Switzerland. Tel.: +41 61 387 31 60; Fax: +41 61 387 39 88; E-mail: fussenegger@bsse.ethz.ch

This PDF file includes:

Supplementary Notes: Description of the mathematical model
Supplementary Figure 1: Degron degradation mechanisms.
Supplementary Figure 2: Characterization of the protein tag library.
Supplementary Figure 3: Validation of the six selected ubiquitin fusion constructs tagged with the degrons 3xUbVR, UbR, UbK, UbD, UbS, and UbM.
Supplementary Figure 4: Characterization of the six selected constructs 3xUbVR, UbR, UbK, UbD, UbS, and UbM over time and under the control of different promoters.
Supplementary Figure 5: Inhibition of proteasome activity leads to accumulation of Ub-tagged Dendra2 constructs.
Supplementary Figure 6: The degradation tags as universal tools to regulate CRISPR/dCas9.
Supplementary Figure 7: Resazurin assay with HEK-293 cells exposed to photoconverting light (405 nm) and mean photoconverted Dendra2 fluorescence of the 3xUbVR, UbR, UbK, UbD, UbS, and UbM-tagged ubiquitin fusion constructs.

Supplementary Figure 8: Experimentally measured SEAP concentrations as a function of the half lives.
Supplementary Figure 9: Characterization of the pulse generator elements.
Supplementary Figure 10: Validation of the pulse generator circuit (1).
Supplementary Figure 11: Validation of the pulse generator circuit (2).
Supplementary Figure 12: Validation of the pulse generator circuit (3).
Supplementary Figure 13: Validation of the pulse generator circuit (4).
Supplementary Table 1: Plasmids and oligonucleotides used in this study.

Supplementary Notes | Description of the mathematical model.

To quantitatively understand the molecular dynamics of our Tet gene expression system and the relationships between the two experimentally determined quantities (protein half-lives and SEAP levels) for the different degrons, we constructed a mathematical model describing in detail all involved biomolecular interactions. In this model, the different constructs (P_{hCMV}-Tag-tTA-Dendra2-pA) consisting of the tetracycline-dependent transactivator tTA fused to Dendra2 and a degradation tag are expressed constitutively with rate v_{tTA}. We assume that, directly after expression, Dendra2 in this construct is in an unmaturated form, and we denote by $\mathrm{tTA}_{\mathrm{pre}}(t)$ the concentration of this unmaturated form at time t. We assume that the chromophore of $\mathrm{tTA}_{\mathrm{pre}}$ maturates with rate constant k_{mat}, either to the neutral (nonfluorescent) or anionic (green fluorescent) form and denote by $\mathrm{tTA}_{\mathrm{neut}}$ the construct with the neutral chromophore, and by $\mathrm{tTA}_{\mathrm{green}}$ the construct with the anionic chromophore. We describe the decision between the two forms with the parameter α; the value of α corresponds to the probability of $\mathrm{tTA}_{\mathrm{green}}$ to maturate to $\mathrm{tTA}_{\mathrm{neut}}$, while $1-\alpha$ corresponds to the probability to maturate to $\mathrm{tTA}_{\mathrm{green}}$. Light with a wavelength of 405 nm photoconverts the neutral form $\mathrm{tTA}_{\mathrm{neut}}$ to the red fluorescent form, which we denote by $\mathrm{tTA}_{\mathrm{red}}$. At any given time, the rate of this photoconversion is governed by the light intensity $u(t)$ in a small band around 405 nm and the parameter ξ describing the efficiency of photoconversion. We assume that all four forms of the construct ($\mathrm{tTA}_{\mathrm{pre}}$, $\mathrm{tTA}_{\mathrm{green}}$, $\mathrm{tTA}_{\mathrm{neut}}$, and $\mathrm{tTA}_{\mathrm{red}}$) are degraded and diluted (due to cell growth and division) with approximately the same rate constant $k_{\mathrm{D,tTA}}(\mathrm{tag})$, which is a function of the degradation tag fused to the respective construct. We assume that the differences in the activation of downstream gene transcription of SEAP by the four forms is negligible, such that the rate of SEAP transcription only depends on the total concentration $\mathrm{tTA}_{\mathrm{tot}}(t) = \mathrm{tTA}_{\mathrm{pre}} + \mathrm{tTA}_{\mathrm{green}}(\mathrm{t}) + \mathrm{tTA}_{\mathrm{neut}}(\mathrm{t}) + \mathrm{tTA}_{\mathrm{red}}(t)$ of the construct. We assume that SEAP can freely diffuse through the cell membrane such that the SEAP concentration is approximately the same in each cell and in the extracellular medium. In agreement with previous work[1] we furthermore assume that the activation of SEAP by $\mathrm{tTA}_{\mathrm{tot}}$ is described by a Hill function with Hill coefficient two, dissociation constant K_{tTA} and maximal expression rate v_{SEAP}. Finally, $k_{\mathrm{D,SEAP}}$ is the degradation rate constant of SEAP. Given these assumptions, our model is described by the following set of ordinary differential equations.

(1) $\frac{d}{dt}\mathrm{tTA}_{\mathrm{pre}}(t) = v_{\mathrm{tTA}} - k_{\mathrm{mat}}\,\mathrm{tTA}_{\mathrm{pre}}(t) - k_{\mathrm{D,tTA}}(\mathrm{tag})\,\mathrm{tTA}_{\mathrm{pre}}(t)$

(2) $\frac{d}{dt}\mathrm{tTA}_{\mathrm{green}}(t) = (1-\alpha)\,k_{\mathrm{mat}}\,\mathrm{tTA}_{\mathrm{pre}}(t) - k_{\mathrm{D,tTA}}(\mathrm{tag})\,\mathrm{tTA}_{\mathrm{green}}(t)$

(3) $\frac{d}{dt}\mathrm{tTA}_{\mathrm{neut}}(t) = \alpha\,k_{\mathrm{mat}}\,\mathrm{tTA}_{\mathrm{pre}}(t) - u(t)\,\xi\,\mathrm{tTA}_{\mathrm{neut}}(t) - k_{\mathrm{D,tTA}}(\mathrm{tag})\,\mathrm{tTA}_{\mathrm{neut}}(t)$

(4) $\frac{d}{dt}\mathrm{tTA}_{\mathrm{red}}(t) = u(t)\,\xi\,\mathrm{tTA}_{\mathrm{neut}}(t) - k_{\mathrm{D,tTA}}(\mathrm{tag})\,\mathrm{tTA}_{\mathrm{red}}(t)$

(5) $\frac{d}{dt}\mathrm{SEAP}(t) = v_{\mathrm{SEAP}}\frac{\mathrm{tTA}_{\mathrm{tot}}^2(t)}{K_{t\mathrm{TA}}^2+\mathrm{tTA}_{\mathrm{tot}}^2(t)} - k_{\mathrm{D,SEAP}}\mathrm{SEAP}$

Given our measurements, most parameters of this model are practically non-identifiable. However, under a few reasonable assumptions it is still possible to derive an expected, approximate relationship between our measurements of half-life times of the constructs, and of the SEAP concentrations which we can then compare to the experimental data. Specifically, we show below that, while we cannot derive expected values for the SEAP concentration from the half-lives for individual degradation tags, when plotting the average SEAP concentration as a function of the average half-life times for all tags, the resulting curve should approximately follow a Hill curve with coefficient two, i.e.

(6) $\langle\mathrm{SEAP}\rangle(\mathrm{tag}) \approx p_a\frac{\langle T_{1/2}\rangle^2(\mathrm{tag})}{p_b^2+\langle T_{1/2}\rangle^2(\mathrm{tag})},$

with $\langle\mathrm{SEAP}\rangle(\mathrm{tag})$ the average measured SEAP concentration for a given tag, $\langle T_{1/2}\rangle(\mathrm{tag}) \approx \frac{\log 2}{k_{\mathrm{D,tTA}}(\mathrm{tag})}$ the corresponding average half-life time, and p_a and p_b two independent fitting parameters.

To derive this formula, we first note that, under the assumption of equal half-life times of the different forms of tTA, the total concentration $\mathrm{tTA}_{\mathrm{tot}}$ follows the differential equation

(7) $$\frac{d}{dt}\mathrm{tTA}_{\mathrm{tot}}(t) = \frac{d}{dt}\mathrm{tTA}_{\mathrm{pre}}(t) + \frac{d}{dt}\mathrm{tTA}_{\mathrm{green}}(t) + \frac{d}{dt}\mathrm{tTA}_{\mathrm{neut}}(t) + \frac{d}{dt}\mathrm{tTA}_{\mathrm{red}}(t)$$
$$= v_{tTA} - k_{\mathrm{D,tTA}}(\mathrm{tag})\mathrm{tTA}_{\mathrm{tot}}(t).$$

For strong degradation tags leading to a short half-life of the construct, we can assume that, at the beginning of the experiment, $\mathrm{tTA}_{\mathrm{tot}}$ has reached its steady-state concentration. In contrast, this might not necessarily be the case for constructs leading to a long half-life time $T_{1/2}(\mathrm{tag}) = \frac{\log 2}{k_{\mathrm{D,tTA}}(\mathrm{tag})}$. If we nevertheless approximate the $\mathrm{tTA}_{\mathrm{tot}}$ by its steady-state concentration for all constructs,

(8) $\mathrm{tTA}_{\mathrm{tot}}^{\mathrm{SS}}(T_{1/2}) = \frac{v_{\mathrm{tTA}}}{k_{\mathrm{D,tTA}}} = \log(2)\;v_{\mathrm{tTA}}\,T_{1/2},$

we thus overestimate the tTA_{tot} concentration by the relative error

(9) $e(T_{1/2},t) = \frac{\mathrm{tTA}_{\mathrm{tot}}^{\mathrm{SS}}(T_{1/2})-\mathrm{tTA}_{\mathrm{tot}}(t)}{\mathrm{tTA}_{\mathrm{tot}}^{\mathrm{SS}}(T_{1/2})}$

which depends on the half-life time $T_{1/2}$ (via the dependency of the half-life time $T_{1/2}(\text{tag})$ on the tag), as well as on the time t. The relative error can take values between zero and one, $0 \leq e(T_{1/2}, t) \leq 1$, where zero corresponds to tTA_{tot} having reached its steady-state, and one to tTA_{tot} having zero concentration. Our assumption that constructs with a small half-life time have already approximately reached their steady-state value at the beginning of the experiment implies that the relative error is small, $e(T_{1/2}, t) \ll 1$, whenever $T_{1/2}$ is small. The relative error however monotonically increases with the half life time of the construct, $\frac{d}{dT_{1/2}} e(T_{1/2}, t) > 1$, but decreases with the experimental time, $\frac{d}{dt} e(T_{1/2}, t) < 1$, since constructs with a long half-life time also eventually approach their steady-state concentration.

Since we placed the cells in fresh medium at the start of the experiment, we can assume that the initial SEAP concentration was approximately zero. Since SEAP concentrations were measured at $T = 24\,\text{h}$ and SEAP is rather stable with an half-life estimated to be as long as 21 days[2], degradation of SEAP can be neglected, corresponding to setting $k_{D,\text{SEAP}} = 0$ in our model. SEAP concentrations at the time of measurement are thus expected to closely depend on the half-life time of the respective construct as described by the following integral equation

$$
\begin{aligned}
(10)\quad \text{SEAP}_{\text{T}}(T_{1/2}) &\approx \int_0^T v_{\text{SEAP}} \frac{\text{tTA}_{\text{tot}}^2(t)}{K_{t\text{TA}}^2 + \text{tTA}_{\text{tot}}^2(t)} dt \\
&= \int_0^T v_{\text{SEAP}} \frac{\left(\left(1 - e(T_{1/2}, t)\right) \text{tTA}_{\text{tot}}^{\text{SS}}(T_{1/2})\right)^2}{K_{\text{tTA}}^2 + \left(\left(1 - e(T_{1/2}, t)\right) \text{tTA}_{\text{tot}}^{\text{SS}}(T_{1/2})\right)^2} dt \\
&= \int_0^T v_{\text{SEAP}} \frac{\left(1 - e(T_{1/2}, t)\right)^2 T_{1/2}^2}{\left(\frac{K_{\text{tTA}}}{\log(2)\, v_{\text{tTA}}}\right)^2 + \left(1 - e(T_{1/2}, t)\right)^2 T_{1/2}^2} dt.
\end{aligned}
$$

For constructs with short half-life times, $e(T_{1/2}, t) \approx 0$, such that the integral is approximately given by

$$
(11)\qquad \text{SEAP}_{\text{T}}(T_{1/2}) \approx p_a \frac{T_{1/2}^2}{p_b^2 + T_{1/2}^2},
$$

with $p_a = v_{\text{SEAP}}\, T$ and $p_b = \frac{K_{\text{tTA}}}{\log(2)\, v_{\text{tTA}}}$. For constructs with a long half-life time, in contrast, $e(T_{1/2}, t)$ might not be close to zero. However, since we used a comparatively strong constitutive promoter for tTA (corresponding to v_{tTA} being high), we might hypothesize that exactly those constructs with a long half-life time should correspond to experimental

conditions for which the promoter of SEAP is saturated or close to saturation for most of the experimental duration, corresponding to $\text{SEAP}_\text{T}(T_{1/2} \gg 1) \approx p_a$. If this hypothesis is correct, this would imply that we could use the same formula predicting the functional form of the relationship between the measured SEAP concentrations and the half-life times of the respective constructs for all constructs and not only for constructs with short half-life times, for which we originally derived it.

While it is impossible to prove that this last hypothesis is correct given our experimental data, it is however possible to test it a posteriori. Specifically, we can first assume that the hypothesis is correct and the SEAP promoter is already saturated for constructs with a long half-life time. This would then imply that the dependency of the experimentally measured SEAP concentrations on the half-life times should follow a Hill curve with Hill coefficient two, which can be checked by fitting a corresponding Hill curve to the experimental data. Given such a fit, our initial assumption that the hypothesis was true then requires that the fitted Hill curve must already be close to its maximum for the constructs with the longest half-life times. If this is not the case, the contradiction would imply that our initial assumption, that the SEAP promoter is already saturated for constructs with a long half-life time, is false. On the other hand, if the curve is close to its maximum for constructs with long half-life times, this would strongly support our initial assumption that the hypothesis was true, even though it would not represent proof. However, since nearly all mathematical models describing the dynamics of biomolecular networks have to be based on several assumptions due to limited availability of experimental data, we believe that this approach to justify this model assumption a posteriori is justified. Given that the relationship of the experimentally measured SEAP concentrations on the protein half-lives indeed follows approximately a Hill curve with Hill coefficient two, as predicted by the model, and since this curve is approximately saturated for the constructs showing the longest half-live times, we conclude that both experimental measures provide consistent information on the dynamics of the synthetic network (Supplementary Figure 8).

Supplementary Figures

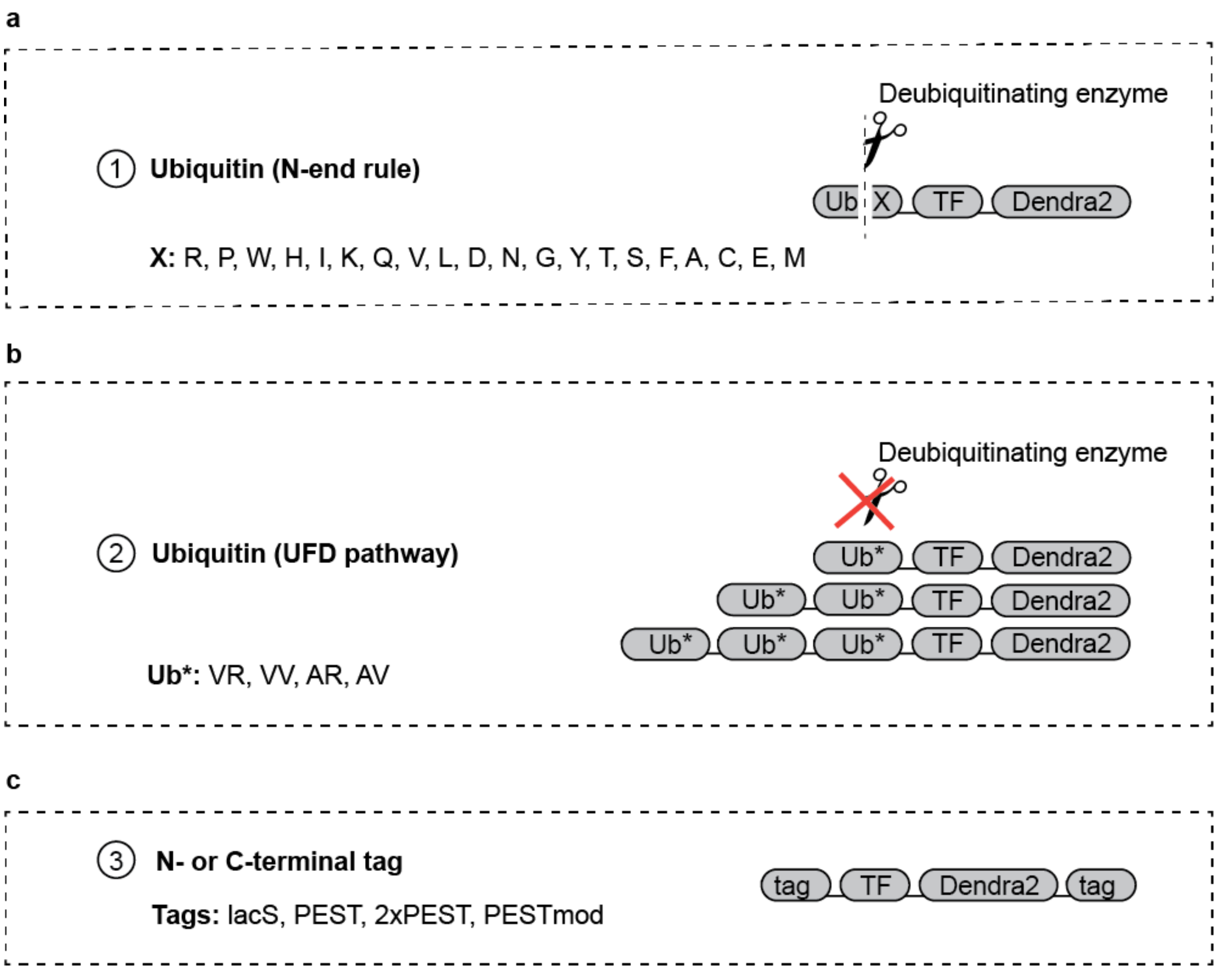

Supplementary Figure 1 | Degron degradation mechanisms.

(**a**) Ubiquitin (N-end rule). Work done in yeast showed that Ub contains a C-terminal isopeptidase site that is recognized by deubiquitinating enzymes, such that Ub is cleaved from the fusion partner after translation to uncover a destabilizing or stabilizing amino acid at the N terminus of the target protein (Fig. 1b)[3, 4]. (**b**) Ubiquitin (UFD pathway). The cleavage rate of the Ub moiety is considerably reduced, leaving polyubiquitinated proteins targeted for proteasomal degradation. (**c**) N- or C-terminal tag. The lacS sequence contains abundant lysine residues[5], and is known to increase the degradation rate of proteins to which it is fused. In the UPP pathway, sequences rich in lysines facilitate the covalent conjugation of Ub, which takes place between the ε-amino group of a lysine residue of the substrate protein and the C-terminal Gly76 of Ub. Ubiquitination of a substrate often yields a substrate-linked multi-Ub chain, in which the C-terminal glycine of one Ub moiety is conjugated to an internal lysine of an adjacent Ub moiety, resulting in a chain of Ub-Ub conjugates[5]. While the lacS degron is

based on the UPP pathway, the PEST sequence is a C-terminal degron rich in proline (P), glutamic acid (E), serine (S) and threonine (T), and serves as a proteolytic signal. However, the mechanism through which the PEST sequence influences protein degradation is still unclear.

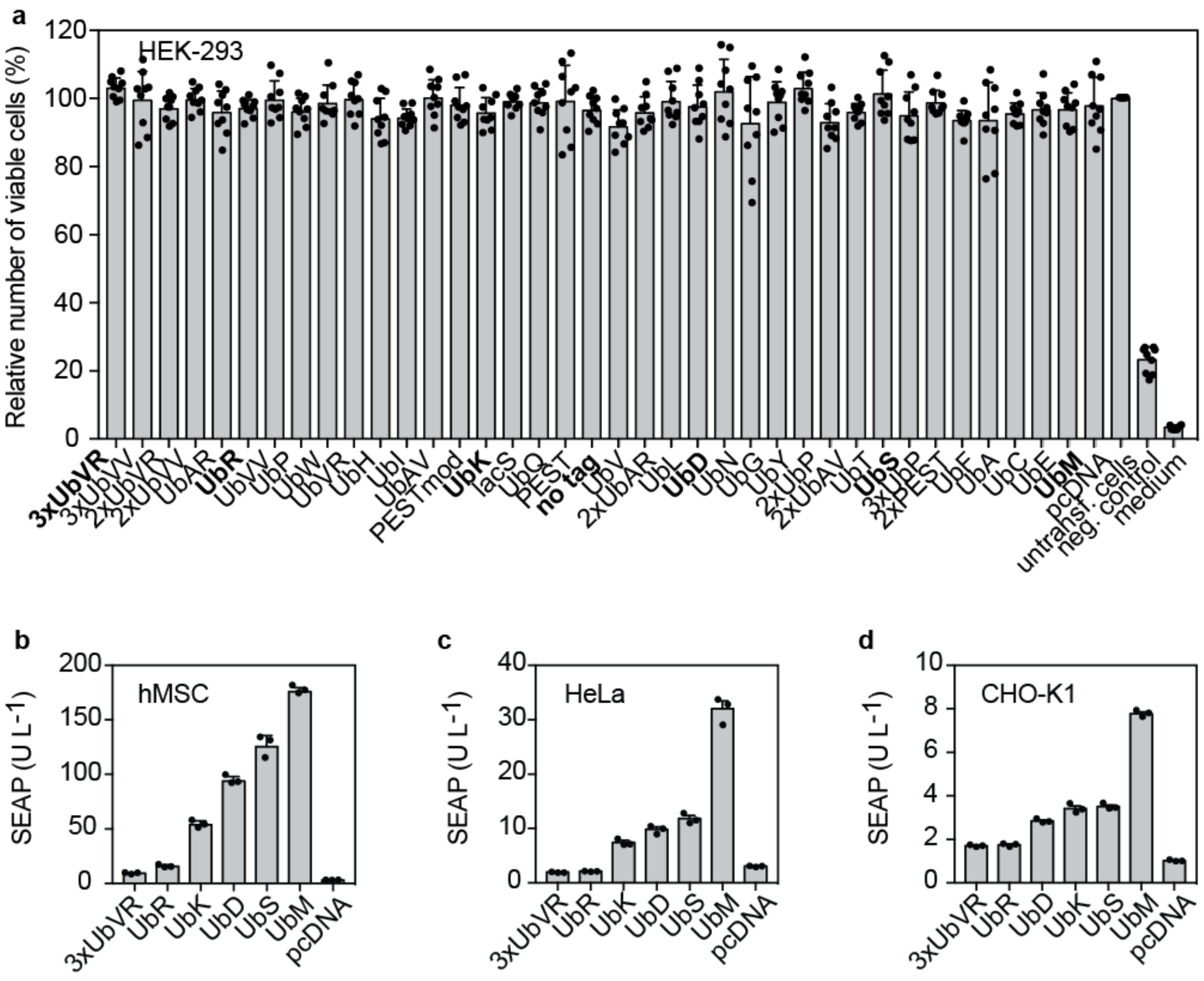

Supplementary Figure 2 | Characterization of the protein tag library.

(**a**) HEK-293 cells were transfected with thirty-six ubiquitin fusion constructs bearing different protein modifications. 3 x 10^4 HEK-293 cells were transfected with the different ubiquitin fusion constructs and resazurin cell viability assay was performed after 24 h. The six ubiquitin fusion constructs selected for further characterization in this work are indicated in bold type. (**b**) 3 x 10^4 hMSC (**c**) 3 x 10^4 HeLa and (**d**) 3 x 10^4 CHO-K1 cells were co-transfected with expression vectors encoding the ubiquitin fusion constructs with the tags 3xUbVR (pCHX50, P_{hCMV}-3x*UbVR-tTA-Dendra2*-pA), UbR (pCHX91, P_{hCMV}-*UbR-tTA-Dendra2*-pA), UbK (pCHX185, P_{hCMV}-*UbK-tTA-Dendra2*-pA), UbD (pCHX178, P_{hCMV}-*UbD-tTA-Dendra2*-pA), UbS (pCHX130, P_{hCMV}-*UbS-tTA-Dendra2*-pA), and UbM (pCHX181, P_{hCMV}-*UbM-tTA-Dendra2*-pA), and the SEAP reporter gene (pMM130, P_{TRE}-*SEAP*-pA). SEAP was profiled in the culture supernatant after 24 h. The data represent mean values ± s.d. (n=3 independent experiments) measured in triplicates.

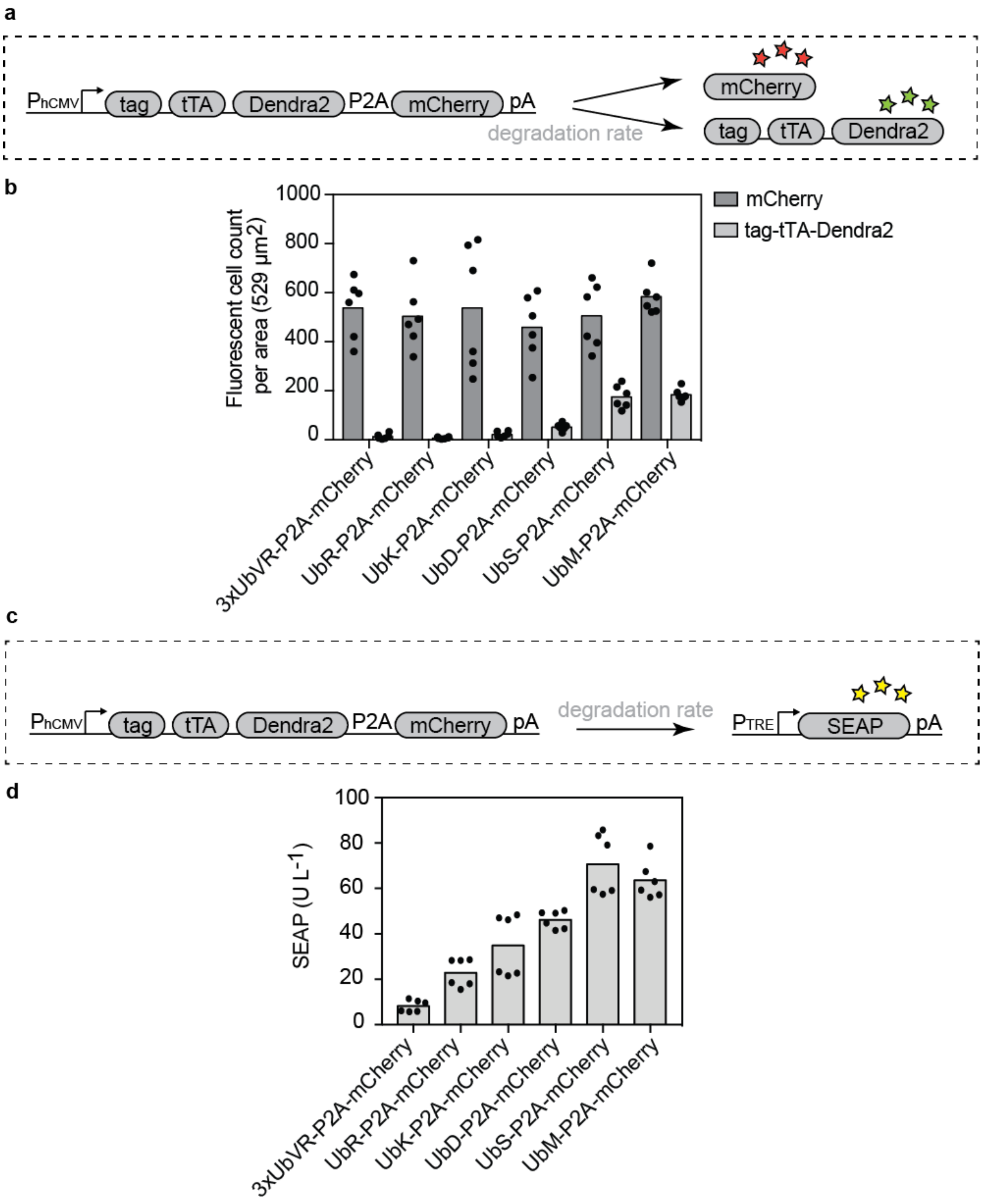

Supplementary Figure 3 | Validation of the six selected ubiquitin fusion constructs tagged with the degrons 3xUbVR, UbR, UbK, UbD, UbS, and UbM.

The diverse SEAP expression shown in Fig. 1c can be attributed to the different degradation rates of the proteins. (**a**) Schematics of the ubiquitin fusion control constructs 3xUbVR, UbR, UbK, UbD, UbS, and UbM equipped with an mCherry fluorescent protein attached via a self-

cleaving P2A peptide. (**b**) 3 x 10^4 HEK-293 cells were transfected with the ubiquitin fusion control constructs 3xUbVR (pCHX246, P_{hCMV}-3x*UbVR-tTA-Dendra2*-P2A-*mCherry*-pA), UbR (pCHX247, P_{hCMV}-*UbR-tTA-Dendra2*-P2A-*mCherry*-pA), UbS (pCHX248, P_{hCMV}-*UbS-tTA-Dendra2*-P2A-*mCherry*-pA), UbK (pCHX249, P_{hCMV}-*UbK-tTA-Dendra2*-P2A-*mCherry*-pA), UbD (pCHX250, P_{hCMV}-*UbD-tTA-Dendra2*-P2A-*mCherry*-pA), and UbM (pCHX251, P_{hCMV}-*UbM-tTA-Dendra2*-P2A-*mCherry*-pA) and analyzed by fluorescence microscopy after 24 h. (**c**) Schematics of the same ubiquitin fusion constructs inducing expression of the SEAP reporter gene (pMM130, P_{TRE}-*SEAP*-pA). (**d**) 3 x 10^4 HEK-293 cells were co-transfected with the ubiquitin fusion control constructs 3xUbVR (pCHX246, P_{hCMV}-3x*UbVR-tTA-Dendra2*-P2A-*mCherry*-pA), UbR (pCHX247, P_{hCMV}-*UbR-tTA-Dendra2*-P2A-*mCherry*-pA), UbS (pCHX248, P_{hCMV}-*UbS-tTA-Dendra2*-P2A-*mCherry*-pA), UbK (pCHX249, P_{hCMV}-*UbK-tTA-Dendra2*-P2A-*mCherry*-pA), UbD (pCHX250, P_{hCMV}-*UbD-tTA-Dendra2*-P2A-*mCherry*-pA), and UbM (pCHX251, P_{hCMV}-*UbM-tTA-Dendra2*-P2A-*mCherry*-pA) and the SEAP reporter gene (pMM130, P_{TRE}-*SEAP*-pA). SEAP in the culture supernatant was profiled after 24 h. The bars represent mean values (n=2 independent experiments) measured in triplicates.

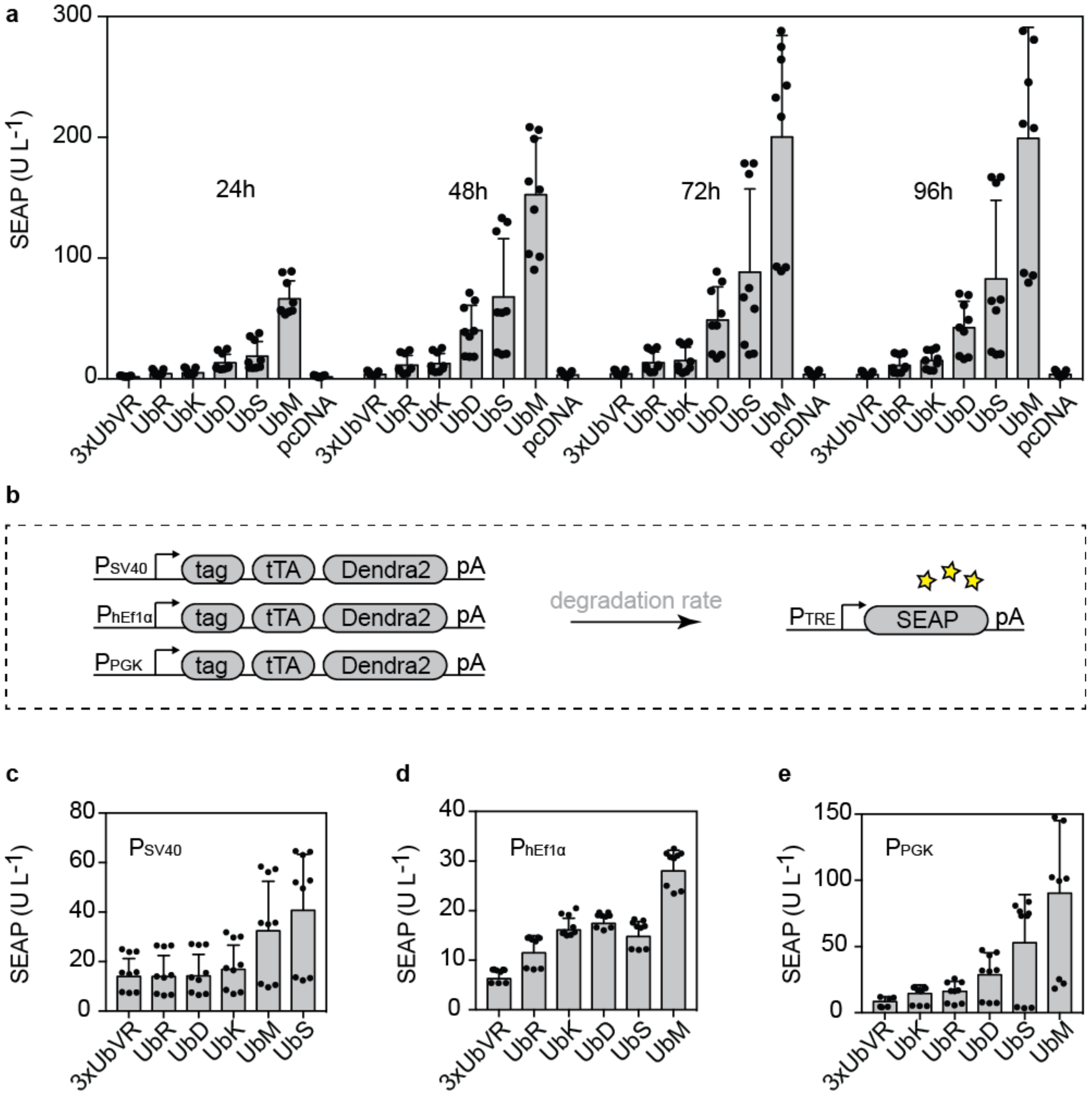

Supplementary Figure 4 | Characterization of the six selected constructs 3xUbVR, UbR, UbK, UbD, UbS, and UbM over time and under the control of different promoters.

(**a**) 3 x 10^4 HEK-293 cells were co-transfected with the ubiquitin fusion constructs 3xUbVR (pCHX50, P_{hCMV}-3x*UbVR-tTA-Dendra2*-pA), UbR (pCHX91, P_{hCMV}-*UbR-tTA-Dendra2*-pA), UbK (pCHX185, P_{hCMV}-*UbK-tTA-Dendra2*-pA), UbD (pCHX178, P_{hCMV}-*UbD-tTA-Dendra2*-pA), UbS (pCHX130, P_{hCMV}-*UbS-tTA-Dendra2*-pA), and UbM (pCHX181, P_{hCMV}-*UbM-tTA-Dendra2*-pA), and the SEAP reporter gene (pMM130, P_{TRE}-*SEAP*-pA). SEAP in the culture supernatant was profiled after 24 h, 48 h, 72 h, and 96 h. (**b**) Schematics of the ubiquitin fusion constructs with three different promoters P_{SV40}, $P_{hEF1\alpha}$, and P_{PGK}. (**c**) 3 x 10^4 HEK-293 cells were co-transfected with the ubiquitin fusion constructs 3xUbVR (pCHX206, P_{SV40}-

3x*UbVR-tTA-Dendra2*-pA), UbR (pCHX147, P_{SV40}-*UbR-tTA-Dendra2*-pA), UbK (pCHX231, P_{SV40}-*UbK-tTA-Dendra2*-pA), UbD (pCHX221, P_{SV40}-*UbD-tTA-Dendra2*-pA), UbS (pCHX149, P_{SV40}-*UbS-tTA-Dendra2*-pA), and UbM (pCHX222, P_{SV40}-*UbM-tTA-Dendra2*-pA), and the SEAP reporter gene (pMM130, P_{TRE}-*SEAP*-pA). SEAP in the culture supernatant was profiled after 24 h. (**d**) 3 x 10^4 HEK-293 cells were co-transfected with the ubiquitin fusion constructs 3xUbVR (pCHX205, $P_{hEF1\alpha}$-3x*UbVR-tTA-Dendra2*-pA), UbR (pCHX188, $P_{hEF1\alpha}$-*UbR-tTA-Dendra2*-pA), UbK (pCHX230, $P_{hEF1\alpha}$-*UbK-tTA-Dendra2*-pA), UbD (pCHX223, $P_{hEF1\alpha}$-*UbD-tTA-Dendra2*-pA), UbS (pCHX190, $P_{hEF1\alpha}$-*UbS-tTA-Dendra2*-pA), and UbM (pCHX224, $P_{hEF1\alpha}$-*UbM-tTA-Dendra2*-pA), and the SEAP reporter gene (pMM130, P_{TRE}-*SEAP*-pA). Due to very strong intrinsic activity of the hEf1α promoter, the plasmids were diluted to 1:16 of the concentration stated in the experimental procedures section. SEAP in the culture supernatant was profiled after 24 h. (**e**) 3 x 10^4 HEK-293 cells were co-transfected with the ubiquitin fusion constructs 3xUbVR (pCHX235, P_{PGK}-3x*UbVR-tTA-Dendra2*-pA), UbR (pCHX163, P_{PGK}-*UbR-tTA-Dendra2*-pA), UbK (pCHX229, P_{PGK}-*UbK-tTA-Dendra2*-pA), UbD (pCHX225, P_{PGK}-*UbD-tTA-Dendra2*-pA), UbS (pCHX165, P_{PGK}-*UbS-tTA-Dendra2*-pA), and UbM (pCHX226, P_{PGK}-*UbM-tTA-Dendra2*-pA), and the SEAP reporter gene (pMM130, P_{TRE}-*SEAP*-pA). SEAP in the culture supernatant was profiled after 24 h. The data represent mean values ± s.d. (n=3 independent experiments) measured in triplicates.

a

b

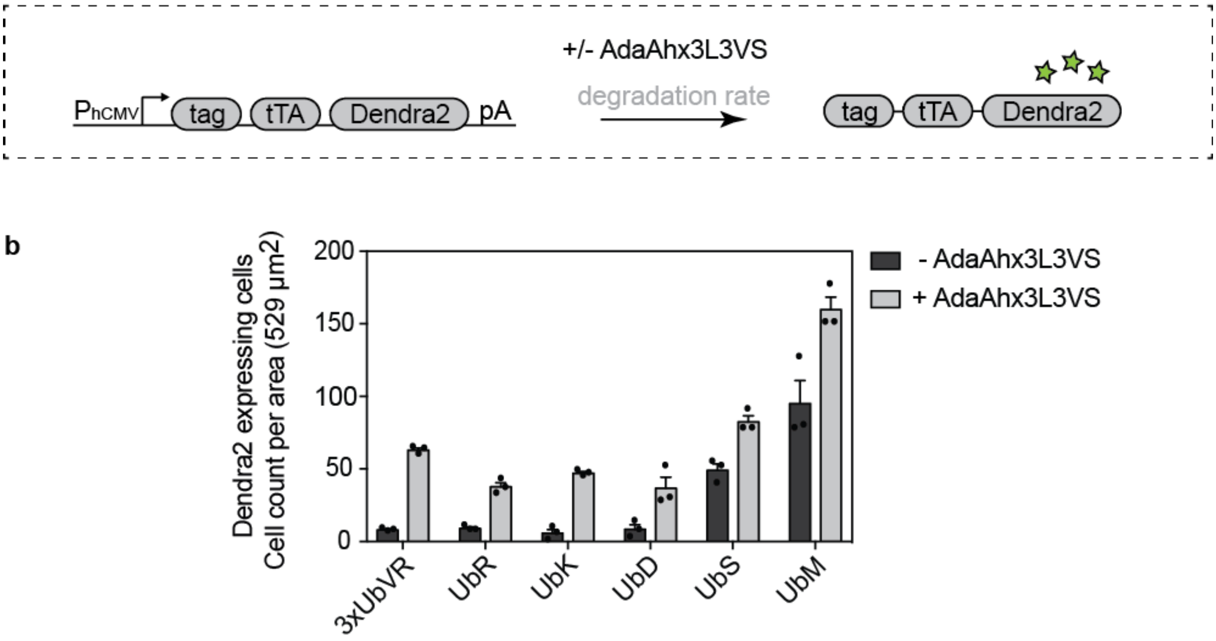

Supplementary Figure 5 | Inhibition of proteasome activity leads to accumulation of Ub-tagged Dendra2 constructs.

(**a**) Schematics of the ubiquitin fusion constructs with and without the addition of the proteasome inhibitor AdaAhx3L3VS. (**b**) Cells expressing the Dendra2 ubiquitin fusion constructs were counted within an area of 529 μm^2 in fluorescence microscopy images of 3 x 10^4 HEK-293 cells transfected with the ubiquitin fusion constructs 3xUbVR (pCHX50, P_{hCMV}-3x*UbVR-tTA-Dendra2*-pA), UbR (pCHX91, P_{hCMV}-*UbR-tTA-Dendra2*-pA), UbK (pCHX185, P_{hCMV}-*UbK-tTA-Dendra2*-pA), UbD (pCHX178, P_{hCMV}-*UbD-tTA-Dendra2*-pA), UbS (pCHX130, P_{hCMV}-*UbS-tTA-Dendra2*-pA), and UbM (pCHX181, P_{hCMV}-*UbM-tTA-Dendra2*-pA). The data represent mean values ± s.d measured in triplicates.

.

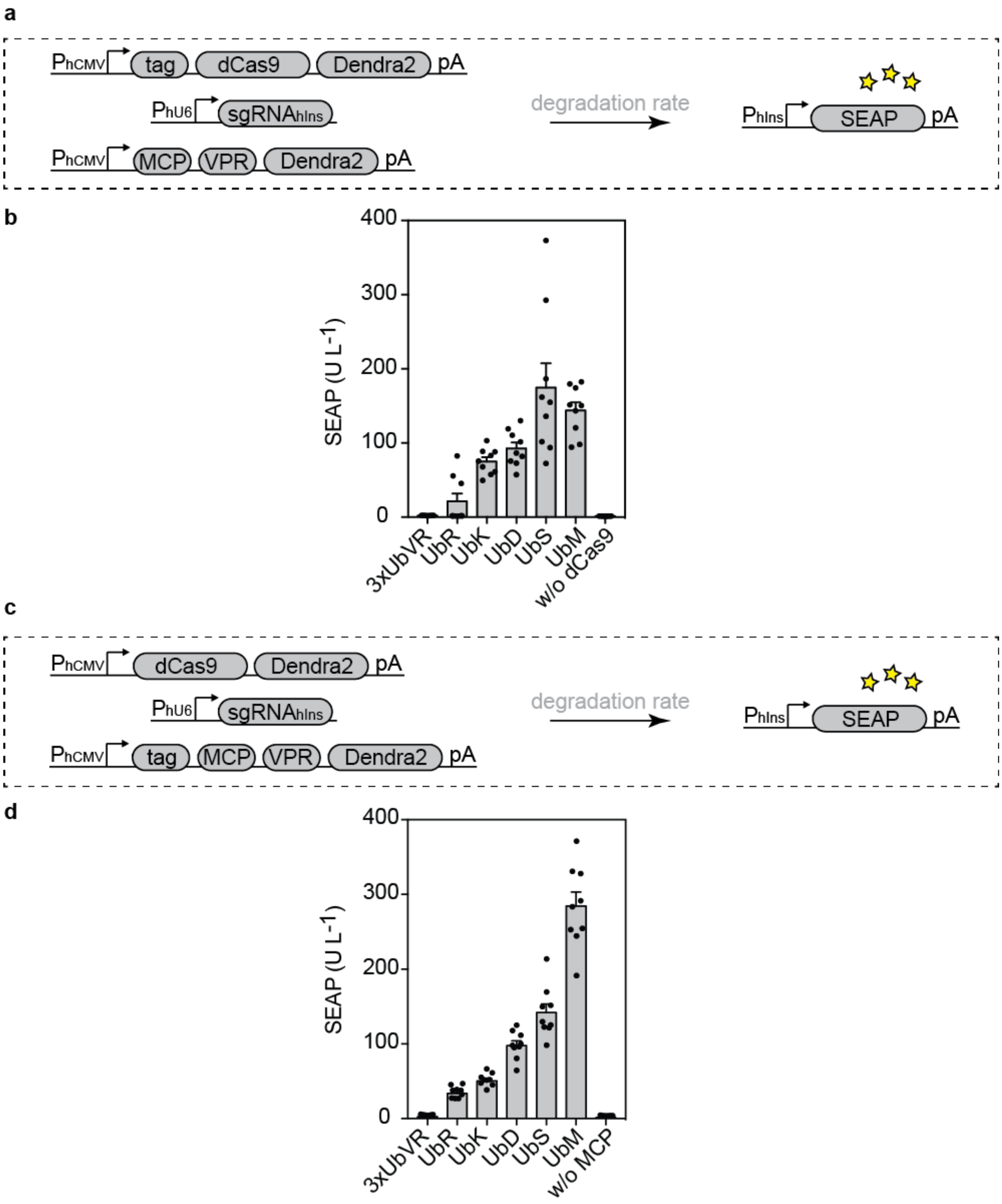

Supplementary Figure 6 | The degradation tags as universal tools to regulate CRISPR/dCas9.

(**a**) Schematics of the dCas9 ubiquitin fusion constructs with and without the addition of RNA-binding MS2 bacteriophage coat protein (MCP). (**b**) 3 x 10^4 HEK-293 cells were co-transfected with the dCas9 ubiquitin fusion constructs 3xUbVR (pLeox5, P_{hCMV}-3x*UbVR-dCas9-Dendra2*-pA), UbR (pLeox7, P_{hCMV}-*UbR-dCas9-Dendra2*-pA), UbK (pLeox17,

P_{hCMV}-*UbK-dCas9-Dendra2*-pA), UbD (pLeox11, P_{hCMV}-*UbD-dCas9-Dendra2*-pA), UbS (pLeox20, P_{hCMV}-*UbS-dCas9-Dendra2*-pA), and UbM (pLeox14, P_{hCMV}-*UbM-dCas9-Dendra2*-pA), the MCP construct pLeox3 (P_{hCMV}-*MCP-VPR-Dendra2*-pA), the gRNA (pGM70, P_{hU6}-sgRNA$_{hINS}$), and the SEAP reporter gene (pSP20, P_{Ins}-*SEAP*-pA). SEAP in the culture supernatant was profiled after 24 h. (**c**) Schematics of the MCP ubiquitin fusion constructs with and without the addition of dCas9. (**d**) 3 x 10^4 HEK-293 cells were co-transfected with the MCP ubiquitin fusion constructs 3xUbVR (pLeox6, P_{hCMV}-3x*UbVR-MCP-VPR-Dendra2*-pA), UbR (pLeox9, P_{hCMV}-*UbR-MCP-VPR-Dendra2*-pA), UbK (pLeox18, P_{hCMV}-*UbK-MCP-VPR-Dendra2*-pA), UbD (pLeox12, P_{hCMV}-*UbD-MCP-VPR-Dendra2*-pA), UbS (pLeox21, P_{hCMV}-*UbS-MCP-VPR-Dendra2*-pA), and UbM (pLeox15, P_{hCMV}-*UbM-MCP-VPR-Dendra2*-pA), the dCas9 construct pLeox2 (P_{hCMV}-*dCas9-Dendra2*-pA), the gRNA (pGM70, P_{hU6}-sgRNA$_{hINS}$), and the SEAP reporter gene (pSP20, P_{Ins}-*SEAP*-pA). SEAP concentration in the culture supernatant was measured after 24 h. The data represent mean values ± s.d. (n=3 independent experiments) measured in triplicates.

a

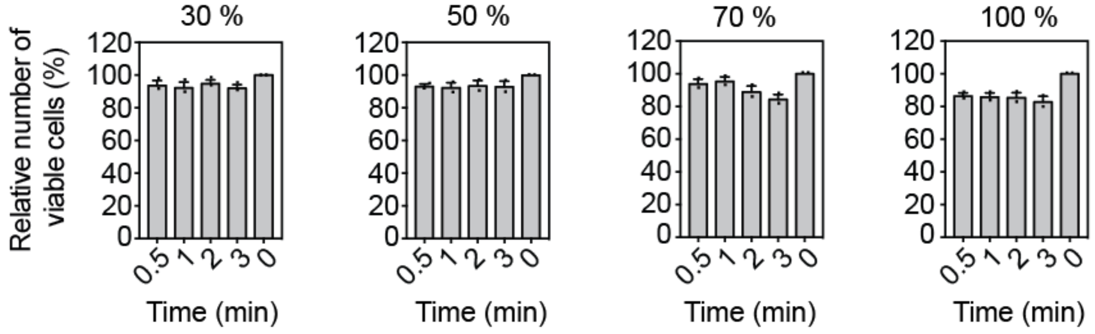
30 %
50 %
70 %
100 %
Relative number of viable cells (%)
Time (min)

b

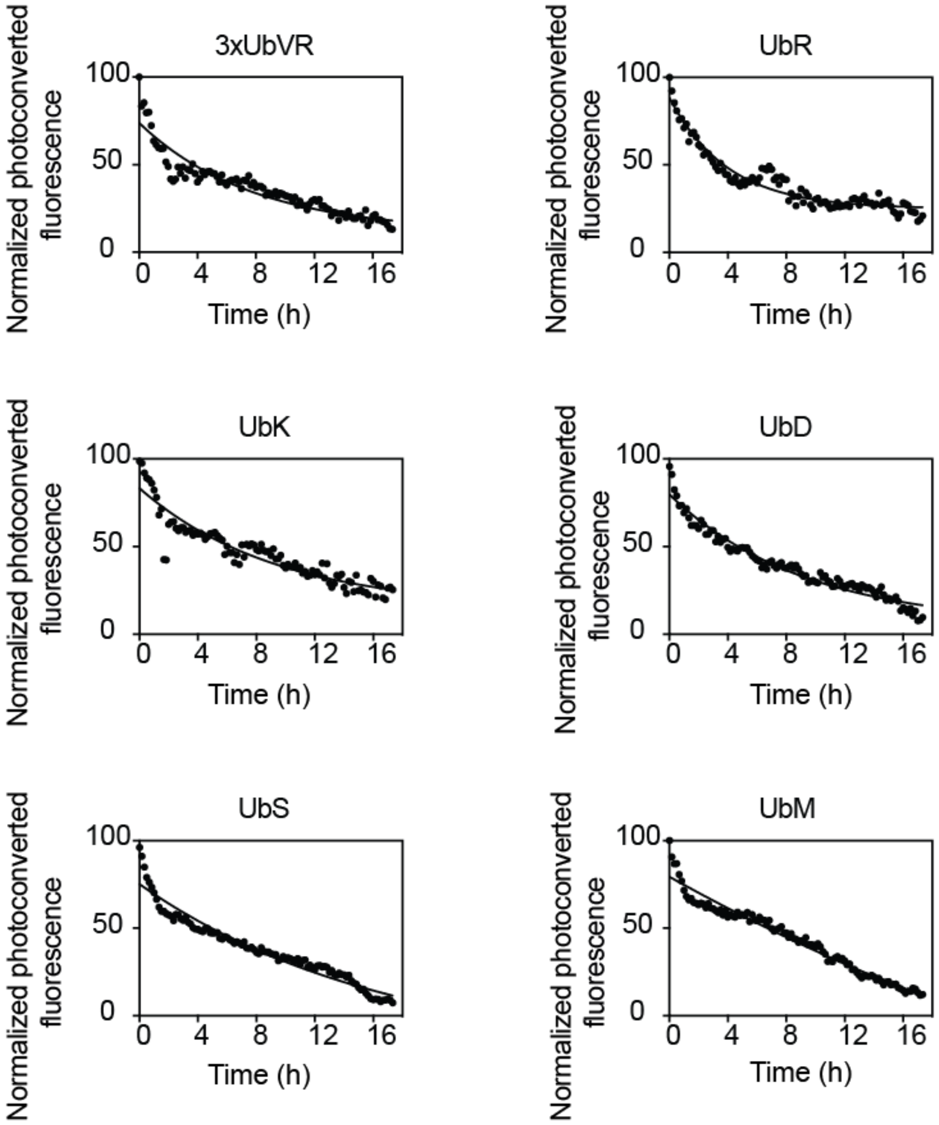
3xUbVR
UbR
UbK
UbD
UbS
UbM
Normalized photoconverted fluorescence
Time (h)

Supplementary Figure 7 | Resazurin assay with HEK-293 cells exposed to photoconverting light (405 nm) and mean photoconverted Dendra2 fluorescence of the 3xUbVR, UbR, UbK, UbD, UbS, and UbM-tagged ubiquitin fusion constructs.

(**a**) 3 x 10^4 HEK-293 cells were exposed for increasing times to 405 nm laser light at 30% of maximum power, 50% of maximum power, 70% of maximum power or 100% of maximum power, as indicated above each plot. Resazurin cell viability assay was performed after 24 h. The data represent mean values ± s.d. (n=3 independent experiments). (**b**) 3 x 10^4 HEK-293 cells were transfected with 3xUbVR (pCHX50, P_{hCMV}-3x*UbVR-tTA-Dendra2*-pA), UbR (pCHX91, P_{hCMV}-*UbR-tTA-Dendra2*-pA), UbK (pCHX185, P_{hCMV}-*UbK-tTA-Dendra2*-pA), UbD (pCHX178, P_{hCMV}-*UbD-tTA-Dendra2*-pA), UbS (pCHX130, P_{hCMV}-*UbS-tTA-Dendra2*-pA), and UbM (pCHX181, P_{hCMV}-*UbM-tTA-Dendra2*-pA). Photoconversion was conducted after 24 h with blue light and fluorescence intensity was evaluated every 10 min. The data represent mean values (n=3 independent experiments) measured in triplicates.

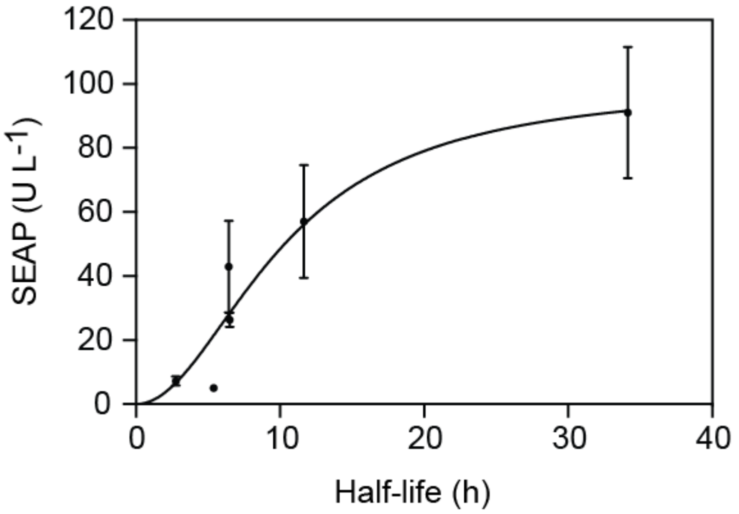

Supplementary Figure 8 | Experimentally measured SEAP concentrations as a function of the half lives.

Data points represent experimentally measured SEAP levels of the six degron tags fused to tTA-Dendra2 (3xUbVR (pCHX50, P_{hCMV}-3x*UbVR-tTA-Dendra2*-pA), UbR (pCHX91, P_{hCMV}-*UbR-tTA-Dendra2*-pA), UbK (pCHX185, P_{hCMV}-*UbK-tTA-Dendra2*-pA), UbD (pCHX178, P_{hCMV}-*UbD-tTA-Dendra2*-pA), UbS (pCHX130, P_{hCMV}-*UbS-tTA-Dendra2*-pA), and UbM (pCHX181, P_{hCMV}-*UbM-tTA-Dendra2*-pA)) and co-transfected with the SEAP reporter gene (pMM130, P_{TRE}-*SEAP*-pA). Expression levels were profiled after 24 h. The connecting line corresponds to the model function. The data represent mean values ± s.d. (n=3 independent experiments) measured in triplicates.

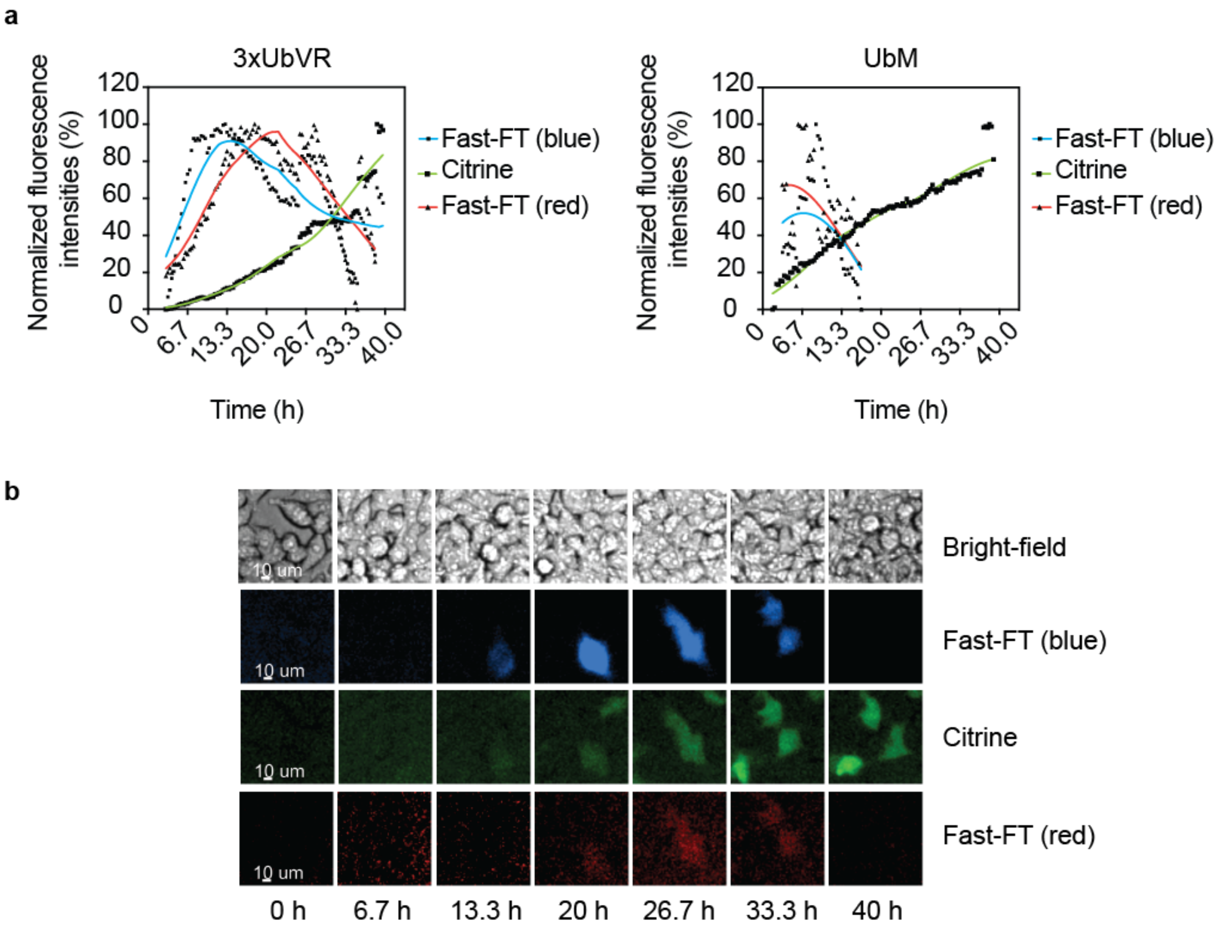

Supplementary Figure 9 | Characterization of the pulse generator elements.

(**a**) Normalized fluorescence intensities of cells transfected either with pCHX301 (P_{TRE}-*TtgR-VP16*-pA:P_{hCMV}-*rtTA*-pA), and pCHX300 (P_{TtgR1}-3x*UbVR-L7Ae*-P2A-*Citrine*-pA:P_{TRE}-*C/D*$_{box}$-*3xUbVR-Fast-FT*-pA) or pCHX308 (P_{TtgR1}-*UbM-L7Ae*-P2A-*Citrine*-pA:P_{TRE}-*C/D*$_{box}$-3x*UbVR-Fast-FT*-pA) upon addition of doxycycline. The fluorescence intensity was evaluated every 20 min for 40 h. The data represent mean values (n=5 independent experiments). (**b**) Time-lapse bright field and fluorescence microscopy images of 3 x 10^4 HEK-293 cells transfected with pCHX301 (P_{TRE}-*TtgR-VP16*-pA:P_{hCMV}-*rtTA*-pA) and pCHX300 (P_{TtgR1}-3x*UbVR-L7Ae*-P2A-*Citrine*-pA:P_{TRE}-C/D_{box}-3x*UbVR-Fast-FT*-pA) upon addition of doxycycline. The images were recorded every 20 min for 40 h. Images at selected time points are shown. The images intensities were all stretched by the same factor (except for the red channel) while maintaining the same intensity ratio for better visibility. White bars correspond to 10 µm.

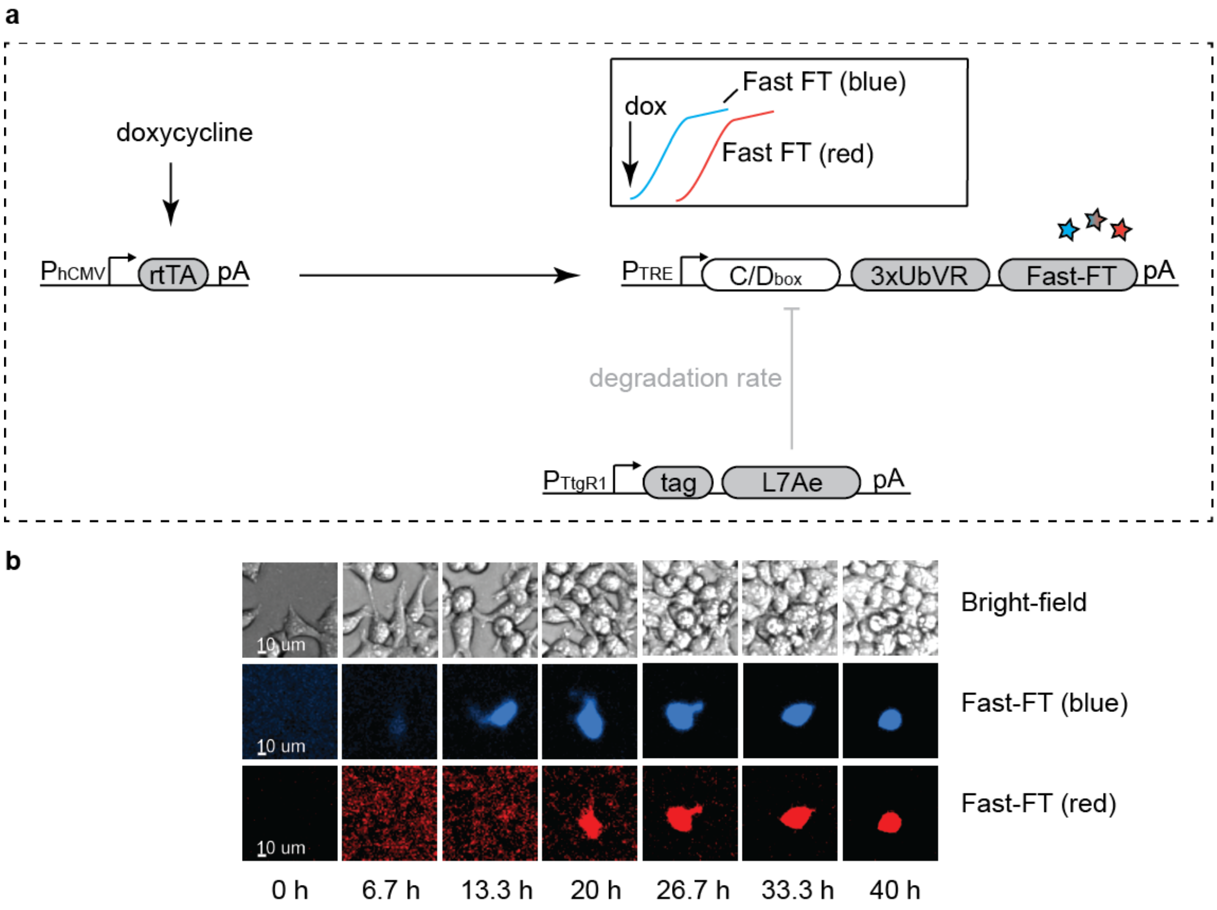

Supplementary Figure 10 | Validation of the pulse generator circuit (1).

(**a**) Schematics of the constitutively expressed rtTA and the 3xUbVR-tagged Fast-FT reporter. (**b**) Time-lapse bright field and fluorescence microscopy images of 3 x 10^4 HEK-293 cells transfected with rtTA (pMM591, P_{hCMV}-*rtTA*-pA) and the 3xUbVR-tagged Fast-FT reporter (pCHX273, P_{TRE}-C/D_{box}-3x*UbVR-Fast-FT*-pA) upon addition of doxycycline. The images were recorded every 20 min for 40 h. Images at selected time points are shown. The images intensities were all stretched by the same factor while maintaining the same intensity ratio for better visibility. White bars correspond to 10 µm.

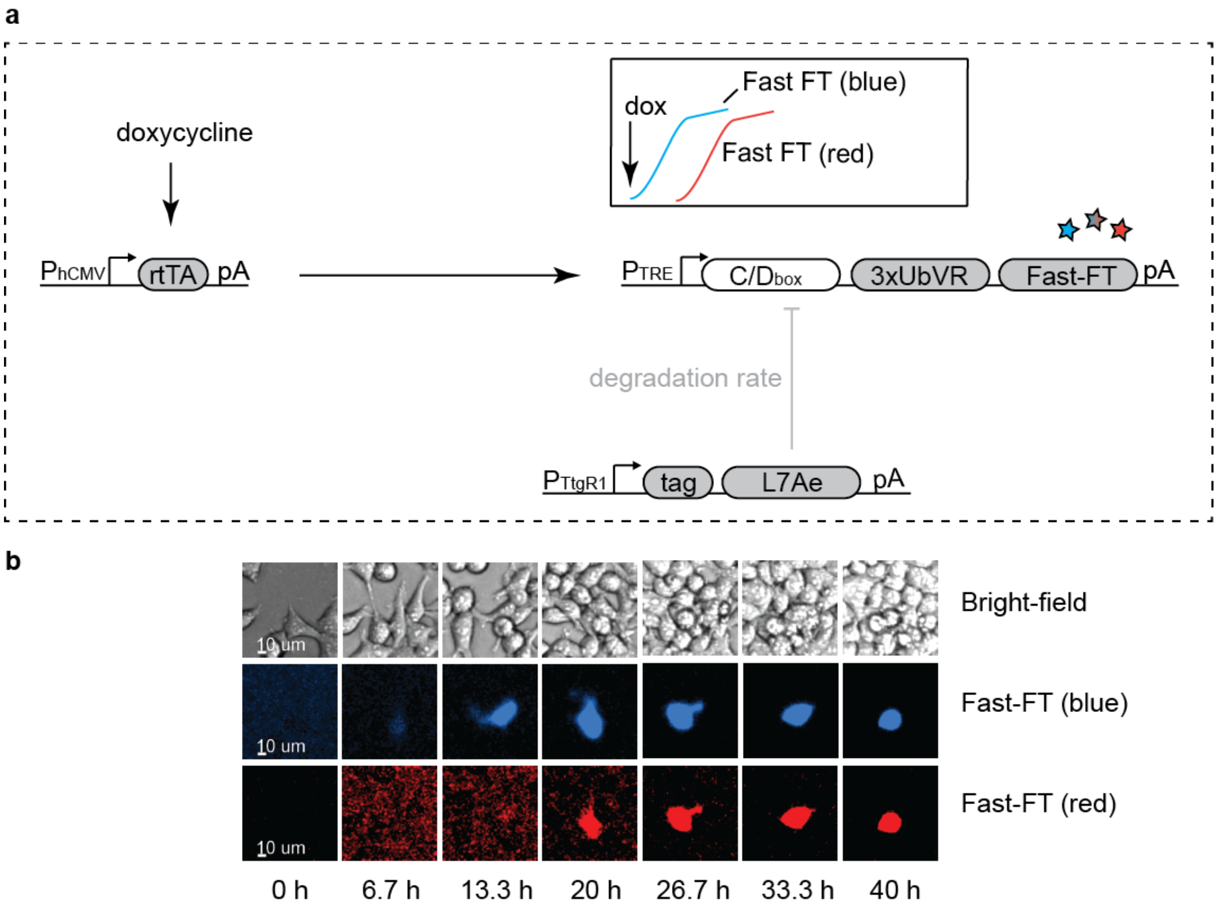

Supplementary Figure 11 | Validation of the pulse generator circuit (2).

(**a**) Schematics of the constitutively expressed rtTA, the 3xUbVR-tagged Fast-FT reporter, and the L7Ae repressor element. (**b**) Time-lapse bright field and fluorescence microscopy images of 3 x 10^4 HEK-293 cells transfected with rtTA (pMM591, P_{hCMV}-*rtTA*-pA), the 3xUbVR-tagged Fast-FT reporter (pCHX273, P_{TRE}-C/D_{box}-3x*UbVR-Fast-FT*-pA), and the L7Ae repressor element (pCHX293, P_{TtgR1}-3x*UbVR-L7Ae*-pA) upon addition of doxycycline. The images were recorded every 20 min for 40 h. Images at selected time points are shown. The images intensities were all stretched by the same factor while maintaining the same intensity ratio for better visibility. White bars correspond to 10 µm.

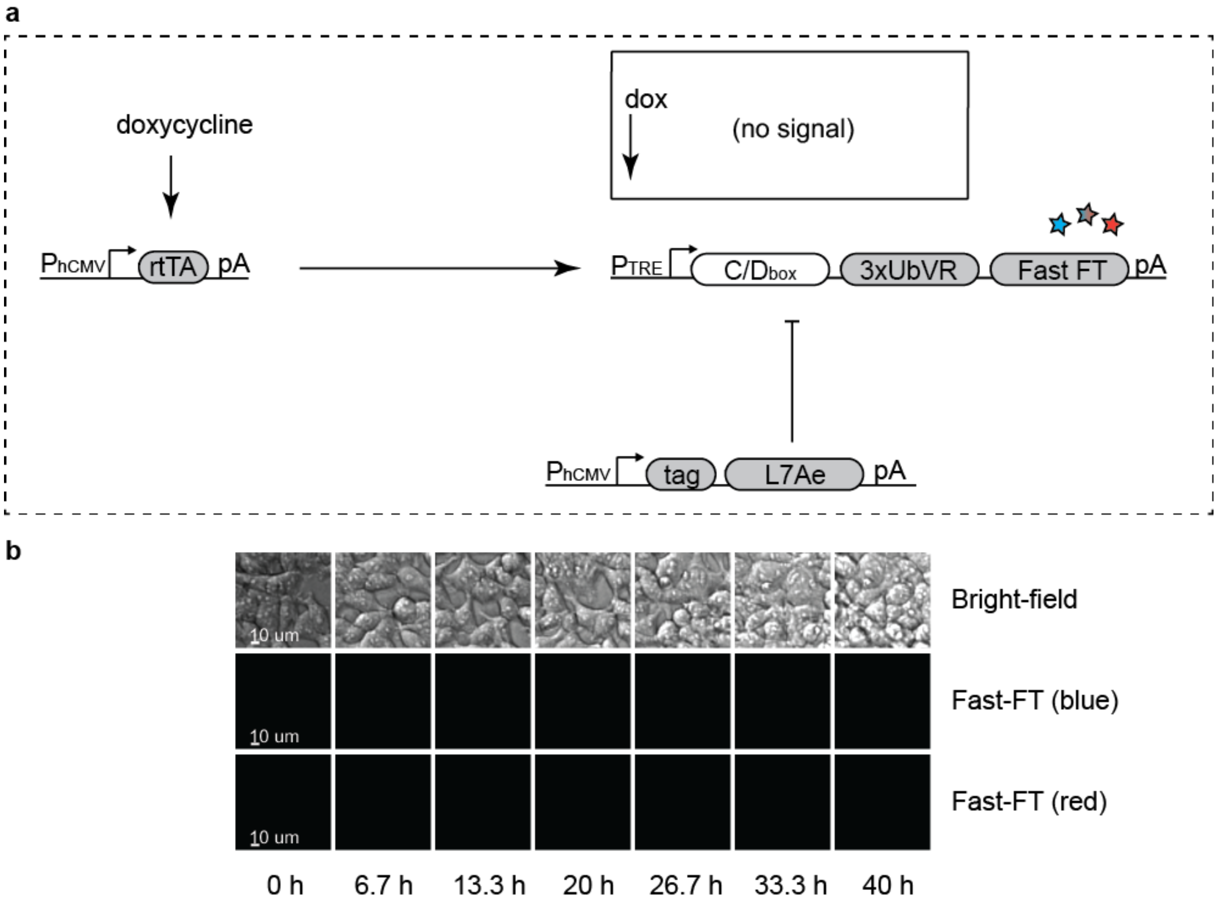

Supplementary Figure 12 | Validation of the pulse generator circuit (3).

(**a**) Schematics of the constitutively expressed rtTA, the 3xUbVR-tagged Fast-FT reporter, and the constitutively expressed L7Ae repressor element. (**b**) Time-lapse bright field and fluorescence microscopy images of 3 x 10^4 HEK-293 cells transfected with rtTA (pMM591, P_{hCMV}-*rtTA*-pA), the 3xUbVR-tagged Fast-FT reporter (pCHX273, P_{TRE}-C/D_{box}-3x*UbVR-Fast-FT*-pA), and the constitutively expressed L7Ae repressor element (pCHX255, P_{hCMV}-3x*UbVR-L7Ae*-pA) upon addition of doxycycline. The images were recorded every 20 min for 40 h. Images at selected time points are shown. The images intensities were all stretched by the same factor while maintaining the same intensity ratio for better visibility. White bars correspond to 10 µm.

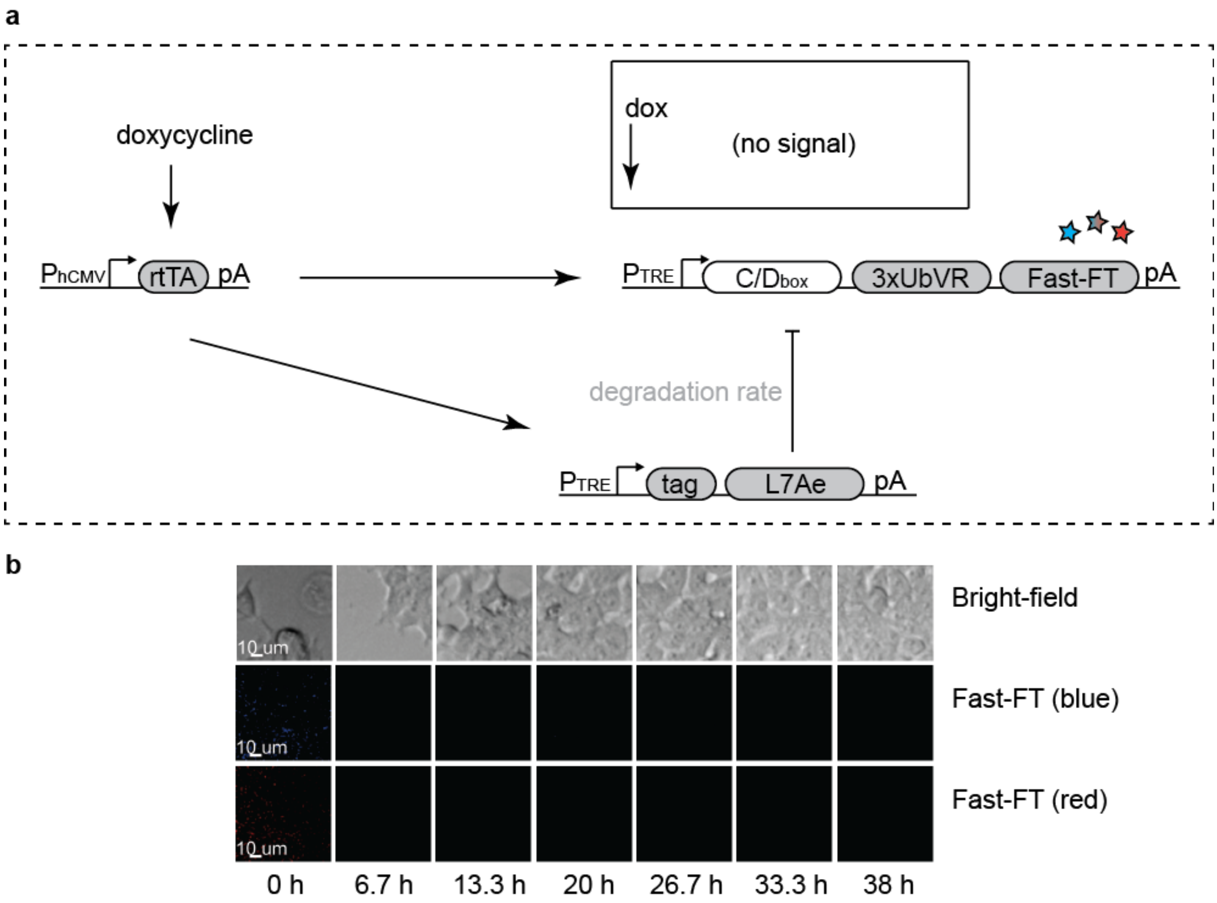

Supplementary Figure 13 | Validation of the pulse generator circuit (4).

(**a**) Schematics of the constitutively expressed rtTA, the 3xUbVR-tagged Fast-FT reporter, and the P_{TRE}-driven L7Ae repressor element. (**b**) Time-lapse bright field and fluorescence microscopy images of 3 x 10^4 HEK-293 cells transfected with rtTA (pMM591, P_{hCMV}-*rtTA*-pA), the 3xUbVR-tagged Fast-FT reporter (pCHX273, P_{TRE}-C/D_{box}-3x*UbVR-Fast-FT*-pA), and the P_{TRE}-driven L7Ae repressor element (pCHX287, P_{TRE}-3x*UbVR-L7Ae*-pA) upon addition of doxycycline. The images were recorded every 20 min for 40 h. Images at selected time points are shown. The images intensities were all stretched by the same factor while maintaining the same intensity ratio for better visibility. White bars correspond to 10 µm.

Supplementary Table 1 | Plasmids used and designed in this study

Plasmid	Description and Cloning Strategy	Reference or Source
p55-H2B-Dendra2	P_{CAG}-driven H2B-Dendra2 expression vector. Addgene plasmid # 80609.	Mohr et al., 2016[1]
pAAV-hEF1a-GCaMP6s-WPRE-pGHpA	$P_{hEF1\alpha}$-driven GCaMP6s expression vector. Addgene plasmid # 67526.	Wertz et al., 2015[2]
pcDNA3.1(+)	P_{hCMV}-driven mammalian expression vector (P_{hCMV}-MCS-pA).	Life Technologies, Zug, CH
pFS29	P_{SV40}-driven mCherry expression vector (P_{SV40}-mCherry-pA).	Auslaender et al., 2014[3]
pMG10	P_{TtgR1}-driven SEAP expression vector (P_{TtgR1}-SEAP-pA).	Gitzinger et al., 2009[4]
pMG11	P_{SV40}-driven TtgR-VP16 expression vector (P_{SV40}-TtgR-VP16-pA).	Gitzinger et al., 2009[4]
pMM1	P_{hCMV}-driven mammalian expression vector (P_{hCMV}-MCS-pA).	Müller et al., 2017[5]
pMM506	P_{hCMV}-driven tTA expression vector (P_{hCMV}-tTA-pA).	Müller et al., 2017[5]
pMM543	P_{hCMV}-driven B3-PEST expression vector (P_{hCMV}-B3-PEST-pA).	Müller et al., 2017[5]
pMM545	P_{hCMV}-driven Citrine expression vector (P_{hCMV}-Citrine-pA).	Müller et al., 2017[5]
pMT100	$P_{hCMV*-1}$-driven Ub-GFP expression vector ($P_{hCMV*-1}$-Ub-GFP-pA).	Tigges et al., 2009[6]
pSA91	P_{ETR2}-driven L7Ae expression vector (P_{ETR2}-L7Ae-pA).	Auslaender et al., 2012[7]
pSUPER retro puro GFP shRNA	P_{H1}-driven GFP shRNA expression vector (P_{H1}-GFP shRNA). Addgene plasmid # 30519.	Pasque et al., 2011[8]
pDB114	P_{PGK}-containing plasmid (P_{H1}-miR124).	Bojar et al., unpublished
pGM70	P_{hU6}-driven sgRNA complementary to a sequence in the human insulin promoter (P_{hU6}-sgRNA$_{hINS}$). OGM99 (5'-CACCGCGGCAGATGGCTGGGGGCTG-3') and OGM100 (5'-AAACCAGCCCCCAGCCATCTGCCGC-3') were annealed together and cloned into BbsI-digested psgRNA(MS2).	Melchner von Dydiowa et al., unpublished
pSEAP2-basic	SEAP-encoding vector (MCS-SEAP-pA).	Clontech, Mountain View, USA
pSEAP2-control	Constitutive mammalian SEAP expression vector (P_{SV40}-SEAP-pA).	Clontech, Mountain View, USA
psgRNA(MS2)	P_{hU6}-driven sgRNA cloning backbone with MS2 loops (P_{hU6}-sgRNA). Addgene plasmid # 61424.	Konermann et al., 2014[9]
pSP20	P_{hINS}-driven SEAP expression vector (P_{hINS}-SEAP-pA).	Saxena et al., unpublished
pTetON-3G	Constitutive rtTA expression vector (P_{hCMV}-rtTA-pA).	Clontech, Mountain View, USA
pTRE-Fast-FT	P_{TRE}-driven Fast Fluorescent Timer expression vector (P_{TRE}-Fast-FT-pA). Addgene plasmid # 31913.	Subach et al., 2009[10]
pUC57	pUC19-derived bacterial expression vector.	GenScript, Piscataway, USA
pVH21	$P_{hEF1\alpha}$-driven expression vector ($P_{hEF1\alpha}$-MCS-pA).	Haellman et al., unpublished
pVH323	P_{hCMV}-driven NLS$_{SV40}$-MCP-VPR expression vector (P_{hCMV}-NLS$_{SV40}$-MCP-VPR-pA).	Haellman et al., unpublished
pVH333	P_{hCMV}-driven dCas9 expression vector (P_{hCMV}-dCas9-pA).	Haellman et al., unpublished

pWB22	P_{TRT}-SEAP expression vector (P_{TRT}-SEAP-pA).	Bacchus et al., 2012[11]
pWB70	P_{hCMV}-driven lacS expression vector (P_{hCMV}-lacS-pA).	Bacchus et al., unpublished
pMM130	P_{TRE}-driven SEAP expression vector (P_{TRE}-SEAP-pA). P_{TRE} was restricted from P_{TRE}-Fast-FT with *Xho*I/*Eco*RI and ligated into the corresponding sites of pSEAP2-basic (*Xho*I/*Eco*RI).	This work
pMM162	P_{TRT}-L7Ae expression vector (P_{TRT}-L7Ae-pA). L7Ae was restricted from pSA91 with *Eco*RI/*Xba*I and cloned into the corresponding sites (*Eco*RI/*Xba*I) of pWB22.	This work
pMM317	P_{hCMV}-driven Lacs-tTA-Dendra2 expression vector (P_{hCMV}-lacS-tTA-Dendra2-pA). LacS was restricted from pWB70 with *Spe*I/*Hin*dIII and cloned into the corresponding sites (*Nhe*I/*Hin*dIII) of pMM600. Dendra2 was excised from pCHX150 digested with *Spe*I and *Hin*dIII and ligated into the corresponding sites of pMM600 (*Nhe*I/*Hin*dIII).	This work
pMM325	P_{PGK}-driven mammalian expression vector (P_{PGK}-MCS-pA). P_{PGK} was PCR-amplified from pDB114 using oligonucleotides OMM284 (5'-ggagatctccacgcgtggtaccctcgagCTACCGGGTAGGGGAGGCGC -3', *Xho*I underlined), and OMM285 (5'-cgcGAATTCGGTCTCCCTATACCGAGCTCGGGCTGGAGGTCGAA AGGCCCGG-3', *Eco*RI underlined), restricted with *Xho*I/*Eco*RI and cloned into the corresponding sites (*Xho*I/*EcoR*I) of pMM1.	This work
pMM334	P_{TRE}-driven TtgR-VP16 expression vector (P_{TRE}-TtgR-VP16-pA). TtgR-VP16 was restricted from pMG11 with *Eco*RI/*Xba*I and cloned into the corresponding sites (*Eco*RI/*Xba*I) of pMM130.	This work
pMM345	P_{TRE}-C/D_{box}-driven SEAP expression vector (P_{TRE}-C/D_{box}-SEAP-pA). SEAP was PCR-amplified from pMM130 using oligonucleotides OMM318 (5'-ctcttacgcgtggtagcccggg-3', *Mlu*I underlined), and OMM321 (5'-gctctagacggatccgctagcCCTGACACCTCTCAGGCGAAG-3', *Eco*RI underlined), and ligated into the corresponding sites of pMM130.	This work
pMM503	P_{hCMV}-driven UbVR expression vector (P_{hCMV}-UbVR-pA). UbVR was PCR-amplified from pMT100 using oligonucleotides OMM67 (5'-cggaattcaccatgactagtCAGATTTTCGTGAAGACCCTG-3', *Eco*RI underlined), and OMM68 (5'-gctctagacggatccgctagcCCTGACACCTCTCAGGCGAAG-3', *Xba*I underlined), restricted with *Eco*RI/*Xba*I and cloned into the corresponding sites (*Eco*RI/*Xba*I) of pMM1.	This work
pMM504	P_{hCMV}-driven UbVV expression vector (P_{hCMV}-UbVV-pA). UbVV was PCR-amplified from pMT100 using oligonucleotides OMM67 (5'-cggaattcaccatgactagtCAGATTTTCGTGAAGACCCTG-3', *Eco*RI underlined), and OMM75 (5'-gctctagacggatccgctagcgacgaCACCTCTCAGGCGAAG-3', *Xba*I underlined), restricted with *Eco*RI/*Xba*I and cloned into the corresponding sites (*Eco*RI/*Xba*I) of pMM1.	This work
pMM505	P_{hCMV}-driven UbP expression vector (P_{hCMV}-UbP-pA). UbP was PCR-amplified from pMT100 using oligonucleotides OMM67 (5'-cggaattcaccatgactagtCAGATTTTCGTGAAGACCCTG-3', *Eco*RI underlined), and OMM76 (5'-gctctagacggatccgctagcaggGCCACCTCTCAGGCGAAG-3', *Xba*I underlined), restricted with *Eco*RI/*Xba*I and cloned into the corresponding sites (*Eco*RI/*Xba*I) of pMM1.	This work
pMM519	P_{hCMV}-driven PEST expression vector (P_{hCMV}-PEST-pA). PEST was PCR-amplified from pMM543 using oligonucleotides OMM109 (5'-gcgaattcaccatgactagtAGCCATGGCTTCCCGCCGG-3', *Eco*RI underlined), and OMM110 (5'-aagcttttctagacaccggtggatccgctagcCACATTGATCCTAGCAGAAGC-3',	This work

	*Xba*I underlined), restricted with *Eco*RI/*Xba*I and cloned into the corresponding sites (*Eco*RI/XbaI) of pMM1.	
pMM520	P_{hCMV}-driven PESTmod expression vector (P_{hCMV}-PESTmod-pA). PCR was performed on pMM519 using oligonucleotides OMM111 (5'-cgtgccagcagcctgctcctccac-3'), and OMM112 (5'-ctgcccatgtcttgtgccc-3'), and self-ligated.	This work
pMM531	P_{hCMV}-driven Fast-FT expression vector (P_{hCMV}-Fast-FT-pA). Fast-FT was PCR-amplified from pTRE-Fast-FT using OMM119 (5'-gcgaattcaccatgactagtGTGAGCAAGGGCGAGGAGG-3', *Eco*RI underlined), and OMM120 (5'-aagcttttctagaCaccggtggatccgctagcCTTGTACAGCTCGTCCATG-3', *Xba*I underlined), restricted with *Eco*RI/*Xba*I and cloned into the corresponding sites (*Eco*RI/*Xba*I) of pMM1.	This work
pMM546	P_{hCMV}-driven L7Ae expression vector (P_{hCMV}-L7Ae-pA). L7Ae was PCR-amplified from pMM162 using oligonucleotides OMM141 (5'-gcgaattcaccatgactagtTACGTGCGCTTCGAGGTGCCCG-3', *Eco*RI underlined), and OMM142 (5'-aagcttttctagacaccggtggatccgctagcCTTCTGCAGGCCCTTGATC-3', *Xba*I underlined), restricted with *Eco*RI/*Xba*I and cloned into the corresponding sites (*Eco*RI/*Xba*I) of pMM1.	This work
pMM591	P_{hCMV}-driven rtTA expression vector (P_{hCMV}-rtTA-pA). rtTA was PCR-amplified from pTetON-3G using oligonucleotides OMM251 (5'-gcggaattcaccatgactagtGGATCAAGACTGGACAAGAG-3', *Eco*RI underlined), and OMM249 (5'-aagctttctagacaccggtggatccgctagcCCCGGGGAGCATGTCAAG-3', *Xba*I underlined), restricted with *Eco*RI/*Xba*I and cloned into the corresponding sites (*Eco*RI/*Xba*I) of pMM1.	This work
pMM600	P_{hCMV}-driven lac spacer expression vector (P_{hCMV}-lacS-pA). LacS was PCR-amplified from pWB70 using oligonucleotides OMM268 (5'-gcggaattcaccatgactagtcACGGCTCCGGAGCTTGGCTG-3', *Eco*RI underlined), and OMM269 (5'-aagctttctagacaccggtggatccgctagcTCGGGAAACCTGTCGTGCCAG-3', *Xba*I underlined), restricted with *Eco*RI/*Xba*I and cloned into the corresponding sites (*Eco*RI/*Xba*I) of pMM1.	This work
pCHX7	P_{hCMV}-driven 2xUbVR expression vector (P_{hCMV}-2xUbVR-pA). UbVR was restricted from pMM503 using *Spe*I/*Bam*HI, and cloned into the corresponding sites (*Nhe*I/*Bam*HI) of pMM503.	This work
pCHX8	P_{hCMV}-driven 2xUbVV expression vector (P_{hCMV}-2xUbVV-pA). UbVV was restricted from pMM504 using *Spe*I/*Bam*HI, and cloned into the corresponding sites (*Nhe*I/*Bam*HI) of pMM504.	This work
pCHX9	P_{hCMV}-driven 2xUbP expression vector (P_{hCMV}-2xUbP-pA). UbP was restricted from pMM505 using *Spe*I/*Bam*HI, and cloned into the corresponding sites (*Nhe*I/*Bam*HI) of pMM505.	This work
pCHX17	P_{hCMV}-driven tTA-Dendra2 expression vector (P_{hCMV}-tTA-Dendra2-pA). Dendra2 was restricted from pCHX150 with *Spe*I/*Bam*HI and cloned into the corresponding sites (*Nhe*I/*Bam*HI) of pMM506.	This work
pCHX19	P_{hCMV}-driven 2xUbVR-tTA-Dendra2 expression vector (P_{hCMV}-2xUbVR-tTA-Dendra2-pA). tTA-Dendra2 was restricted from pCHX17 using *Spe*I/*Bam*HI, and cloned into the corresponding sites (*Nhe*I/*Bam*HI) of pCHX7.	This work
pCHX20	P_{hCMV}-driven 2xUbVV-tTA-Dendra2 expression vector (P_{hCMV}-2xUbVV-tTA-Dendra2-pA). tTA-Dendra2 was restricted from pCHX17 using *Spe*I/*Bam*HI, and cloned into the corresponding sites (*Nhe*I/*Bam*HI) of pCHX8.	This work

pCHX21	P_{hCMV}-driven 2xUbP-tTA-Dendra2 expression vector (P_{hCMV}-2xUbP-tTA-Dendra2-pA). tTA-Dendra2 was restricted from pCHX17 using *Spe*I/*Bam*HI, and cloned into the corresponding sites (*Nhe*I/*Bam*HI) of pCHX9.	This work
pCHX22	P_{hCMV}-driven UbVR-tTA-Dendra2 expression vector (P_{hCMV}-UbVR-tTA-Dendra2-pA). tTA-Dendra2 was restricted from pCHX17 using *Spe*I/*Bam*HI, and cloned into the corresponding sites (*Nhe*I/*Bam*HI) of pCHX503.	This work
pCHX23	P_{hCMV}-driven UbVV-tTA-Dendra2 expression vector (P_{hCMV}-UbVV-tTA-Dendra2-pA). tTA-Dendra2 was restricted from pCHX17 using *Spe*I/*Bam*HI, and cloned into the corresponding sites (*Nhe*I/*Bam*HI) of pCHX504.	This work
pCHX24	P_{hCMV}-driven UbP-tTA-Dendra2 expression vector (P_{hCMV}-UbP-tTA-Dendra2-pA). tTA-Dendra2 was restricted from pCHX17 using *Spe*I/*Bam*HI, and cloned into the corresponding sites (*Nhe*I/*Bam*HI) of pCHX505.	This work
pCHX31	P_{hCMV}-driven tTA-Dendra2-PESTmod expression vector (P_{hCMV}-tTA-Dendra2-PESTmod-pA). PESTmod was restricted from pMM520 using *Spe*I/*Bam*HI, and cloned into the corresponding sites (*Nhe*I/*Bam*HI) of pCHX17.	This work
pCHX33	P_{hCMV}-driven tTA-Dendra2-PEST expression vector (P_{hCMV}-tTA-Dendra2-PEST-pA). PEST was restricted from pMM519 using *Spe*I/*Bam*HI, and cloned into the corresponding sites (*Nhe*I/*Bam*HI) of pCHX17.	This work
pCHX35	P_{hCMV}-driven 3xUbVR expression vector (P_{hCMV}-3xUbVR-pA). UbVR was restricted from pMM503 using *Spe*I/*Bam*HI, and cloned into the corresponding sites (*Nhe*I/*Bam*HI) of pCHX7.	This work
pCHX36	P_{hCMV}-driven 3xUbVV expression vector (P_{hCMV}-3xUbVV-pA). UbVV was restricted from pMM504 using *Spe*I/*Bam*HI, and cloned into the corresponding sites (*Nhe*I/*Bam*HI) of pCHX8.	This work
pCHX37	P_{hCMV}-driven 3xUbP expression vector (P_{hCMV}-3xUbP-pA). UbP was restricted from pMM505 using *Spe*I/*Bam*HI, and cloned into the corresponding sites (*Nhe*I/*Bam*HI) of pCHX9.	This work
pCHX38	P_{TRE}-driven tTA-Dendra2 expression vector (P_{TRE}-tTA-Dendra2-pA). tTA-Dendra2 was restricted from pCHX17 with *Eco*RI/*Xba*I and cloned into the corresponding sites (*Eco*RI/*Xba*I) of pMM130.	This work
pCHX50	P_{hCMV}-driven 3xUbVR-tTA-Dendra2 expression vector (P_{hCMV}-3xUbVR-tTA-Dendra2-pA). tTA-Dendra2 was restricted from pCHX17 using *Spe*I/*Bam*HI, and cloned into the corresponding sites (*Nhe*I/*Bam*HI) of pCHX35.	This work
pCHX51	P_{hCMV}-driven 3xUbVV-tTA-Dendra2 expression vector (P_{hCMV}-3xUbVV-tTA-Dendra2-pA). tTA-Dendra2 was restricted from pCHX17 using *Spe*I/*Bam*HI, and cloned into the corresponding sites (*Nhe*I/*Bam*HI) of pCHX36.	This work
pCHX52	P_{hCMV}-driven 3xUbP-tTA-Dendra2 expression vector (P_{hCMV}-3xUbP-tTA-Dendra2-pA). tTA-Dendra2 was restricted from pCHX17 using *Spe*I/*Bam*HI, and cloned into the corresponding sites (*Nhe*I/*Bam*HI) of pCHX36.	This work
pCHX61	P_{hCMV}-driven rtTA-Dendra2 expression vector (P_{hCMV}-rtTA-Dendra2-pA). Dendra2 was restricted from pCHX150 using *Spe*I/*Bam*HI, and cloned into the corresponding sites (*Nhe*I/*Bam*HI) of pMM591.	This work

pCHX75	P_{hCMV}-driven UbR expression vector (P_{hCMV}-UbR-pA). UbR was PCR-amplified from pMM505 using oligonucleotides OMM67 (5'-cggaattcaccatgactagtCAGATTTTCGTGAAGACCCTG-3', *Eco*RI underlined), and OMM281 (5'-gctctagacggatccgctagcccgGCCACCTCTCAGGCGAAG-3', *Xba*I underlined), restricted with *Eco*RI/*Xba*I and cloned into the corresponding sites (*Eco*RI/*Xba*I) of pMM1.	This work
pCHX78	P_{hCMV}-driven UbAR expression vector (P_{hCMV}-UbAR-pA). UbAR was PCR-amplified from pMM505 using oligonucleotides OMM67 (5'-cggaattcaccatgactagtCAGATTTTCGTGAAGACCCTG-3', *Eco*RI underlined), and OMM282 (5'-gctctagacggatccgctagcccgGGCACCTCTCAGGCGAAG-3', *Xba*I underlined), restricted with *Eco*RI/*Xba*I and cloned into the corresponding sites (*Eco*RI/*Xba*I) of pMM1.	This work
pCHX79	P_{hCMV}-driven UbAV expression vector (P_{hCMV}-UbAV-pA). UbAV was PCR-amplified from pMM505 using oligonucleotides OMM67 (5'-cggaattcaccatgactagtCAGATTTTCGTGAAGACCCTG-3', *E*coRI underlined), and OMM283 (5'-gctctagacggatccgctagccACGGCACCTCTCAGGCGAAG-3', *Xba*I underlined), restricted with *Eco*RI/*Xba*I and cloned into the corresponding sites (*Eco*RI/*Xba*I) of pMM1.	This work
pCHX82	P_{SV40}-driven tTA-Dendra2 expression vector (P_{SV40}-tTA-Dendra2-pA). tTA-Dendra2 was restricted from pCHX17 using *Eco*RI/*Xba*I and cloned into the corresponding sites (*Eco*RI/*Xba*I) of pSEAP2-Control.	This work
pCHX91	P_{hCMV}-driven UbR-tTA-Dendra2 expression vector (P_{hCMV}-UbR-tTA-Dendra2-pA). tTA-Dendra2 was restricted from pCHX17 using *Spe*I/*Bam*HI, and cloned into the corresponding sites (*Nhe*I/*Bam*HI) of pCHX75.	This work
pCHX92	P_{hCMV}-driven UbAR-tTA-Dendra2 expression vector (P_{hCMV}-UbAR-tTA-Dendra2-pA). tTA-Dendra2 was restricted from pCHX17 using *Spe*I/*Bam*HI, and cloned into the corresponding sites (*Nhe*I/*Bam*HI) of pCHX78.	This work
pCHX93	P_{hCMV}-driven UbAV-tTA-Dendra2 expression vector (P_{hCMV}-UbAV-tTA-Dendra2-pA). tTA-Dendra2 was restricted from pCHX17 using *Spe*I/*Bam*HI, and cloned into the corresponding sites (*Nhe*I/*Bam*HI) of pCHX79.	This work
pCHX103	P_{TRE}-driven UbR-tTA-Dendra2 expression vector (P_{TRE}-UbR-tTA-Dendra2-pA). UbR-tTA-Dendra2 was restricted from pCHX91 using *Eco*RI/*Xba*I and cloned into the corresponding sites (*Eco*RI/*Xba*I) of pMM130.	This work
pCHX108	P_{hCMV}-driven UbV expression vector (P_{hCMV}-UbV-pA). UbV was PCR-amplified from pMM505 using oligonucleotides OMM67 (5'-cggaattcaccatgactagtCAGATTTTCGTGAAGACCCTG-3', EcoRI underlined), and OCH122 (5'-gctctagacggatccgctagccacGCCACCTCTCAGGCGAAG-3', *Xba*I underlined), restricted with *Eco*RI/*Xba*I and cloned into the corresponding sites (*Eco*RI/*Xba*I) of pMM1.	This work
pCHX109	P_{hCMV}-driven UbG expression vector (P_{hCMV}-UbG-pA). UbG was PCR-amplified from pMM505 using oligonucleotides OMM67 (5'-cggaattcaccatgactagtCAGATTTTCGTGAAGACCCTG-3', *Eco*RI underlined), and OCH115 (5'-gctctagacggatccgctagcaccGCCACCTCTCAGGCGAAG-3', *Xba*I underlined), restricted with *Eco*RI/*Xba*I and cloned into the corresponding sites (*Eco*RI/*XbaI*) of pMM1.	This work
pCHX110	P_{hCMV}-driven UbI expression vector (P_{hCMV}-UbI-pA). UbI was PCR-amplified from pMM505 using oligonucleotides OMM67 (5'-cggaattcaccatgactagtCAGATTTTCGTGAAGACCCTG-3', *Eco*RI	This work

	underlined), and OCH116 (5'-gctctagacggatccgctagcaatGCCACCTCTCAGGCGAAG-3', *Xba*I underlined), restricted with *Eco*RI/*Xba*I and cloned into the corresponding sites (*Eco*RI/*XbaI*) of pMM1.	
pCHX111	P_{hCMV}-driven UbT expression vector (P_{hCMV}-UbT-pA). UbT was PCR-amplified from pMM505 using oligonucleotides OMM67 (5'-cggaattcaccatgactagtCAGATTTTCGTGAAGACCCTG-3', *EcoR*I underlined), and OCH117 (5'-gctctagacggatccgctagcagtGCCACCTCTCAGGCGAAG-3', *Xba*I underlined), restricted with *Eco*RI/*Xba*I and cloned into the corresponding sites (*Eco*RI/*Xba*I) of pMM1.	This work
pCHX112	P_{hCMV}-driven UbH expression vector (P_{hCMV}-UbH-pA). UbH was PCR-amplified from pMM505 using oligonucleotides OMM67 (5'-cggaattcaccatgactagtCAGATTTTCGTGAAGACCCTG-3', EcoRI underlined), and OCH118 (5'-gctctagacggatccgctagcatgGCCACCTCTCAGGCGAAG-3', *Xba*I underlined), restricted with EcoRI/*Xba*I and cloned into the corresponding sites (*Eco*RI/*Xba*I) of pMM1.	This work
pCHX113	P_{hCMV}-driven UbS expression vector (P_{hCMV}-UbS-pA). UbS was PCR-amplified from pMM505 using oligonucleotides OMM67 (5'-cggaattcaccatgactagtCAGATTTTCGTGAAGACCCTG-3', *Eco*RI underlined), and OCH119 (5'-gctctagacggatccgctagcactGCCACCTCTCAGGCGAAG-3', *Xba*I underlined), restricted with *Eco*RI/*Xba*I and cloned into the corresponding sites (*Eco*RI/*Xba*I) of pMM1.	This work
pCHX114	P_{hCMV}-driven UbQ expression vector (P_{hCMV}-UbQ-pA). UbQ was PCR-amplified from pMM505 using oligonucleotides OMM67 (5'-cggaattcaccatgactagtCAGATTTTCGTGAAGACCCTG-3', *Eco*RI underlined), and OCH120 (5'-gctctagacggatccgctagcctgGCCACCTCTCAGGCGAAG-3', *Xba*I underlined), restricted with *Eco*RI/*Xba*I and cloned into the corresponding sites (*Eco*RI/*Xba*I) of pMM1.	This work
pCHX115	P_{hCMV}-driven UbL expression vector (P_{hCMV}-UbL-pA). UbL was PCR-amplified from pMM505 using oligonucleotides OMM67 (5'-cggaattcaccatgactagtCAGATTTTCGTGAAGACCCTG-3', *Eco*RI underlined), and OCH121 (5'-gctctagacggatccgctagccagGCCACCTCTCAGGCGAAG-3', *Xba*I underlined), restricted with *Eco*RI/*Xba*I and cloned into the corresponding sites (*Eco*RI/*Xba*I) of pMM1.	This work
pCHX116	P_{hCMV}-driven 2xUbAV-tTA-Dendra2 expression vector (P_{hCMV}-2xUbAV-tTA-Dendra2-pA). UbAV-tTA-Dendra2 was restricted from pCHX93 using *Spe*I/*Bam*HI, and cloned into the corresponding sites (*Nhe*I/*Bam*HI) of pCHX79.	This work
pCHX117	P_{hCMV}-driven 2xUbAR-tTA-Dendra2 expression vector (P_{hCMV}-2xUbAR-tTA-Dendra2-pA). UbAR-tTA-Dendra2 was restricted from pCHX92 using *Spe*I/*Bam*HI, and cloned into the corresponding sites (*Nhe*I/*Bam*HI) of pCHX78.	This work
pCHX125	P_{hCMV}-driven UbV-tTA-Dendra2 expression vector (P_{hCMV}-UbV-tTA-Dendra2-pA). tTA-Dendra2 was restricted from pCHX17 using *Spe*I/*Bam*HI, and cloned into the corresponding sites (*Nhe*I/*Bam*HI) of pCHX108.	This work
pCHX126	P_{hCMV}-driven UbG-tTA-Dendra2 expression vector (P_{hCMV}-UbG-tTA-Dendra2-pA). tTA-Dendra2 was restricted from pCHX17 using *Spe*I/*Bam*HI, and cloned into the corresponding sites (*Nhe*I/*Bam*HI) of pCHX109.	This work
pCHX127	P_{hCMV}-driven UbI-tTA-Dendra2 expression vector (P_{hCMV}-UbI-tTA-Dendra2-pA). tTA-Dendra2 was restricted from pCHX17 using *Spe*I/*Bam*HI, and cloned into the corresponding sites (*Nhe*I/*Bam*HI) of pCHX110.	This work

pCHX128	P_{hCMV}-driven UbT-tTA-Dendra2 expression vector (P_{hCMV}-UbT-tTA-Dendra2-pA). tTA-Dendra2 was restricted from pCHX17 using *Spe*I/*Bam*HI, and cloned into the corresponding sites (*Nhe*I/*Bam*HI) of pCHX111.	This work
pCHX129	P_{hCMV}-driven UbH-tTA-Dendra2 expression vector (P_{hCMV}-UbH-tTA-Dendra2-pA). tTA-Dendra2 was restricted from pCHX17 using *Spe*I/*Bam*HI, and cloned into the corresponding sites (*Nhe*I/*Bam*HI) of pCHX112.	This work
pCHX130	P_{hCMV}-driven UbS-tTA-Dendra2 expression vector (P_{hCMV}-UbS-tTA-Dendra2-pA). tTA-Dendra2 was restricted from pCHX17 using *Spe*I/*Bam*HI, and cloned into the corresponding sites (*Nhe*I/*Bam*HI) of pCHX113.	This work
pCHX131	P_{hCMV}-driven UbQ-tTA-Dendra2 expression vector (P_{hCMV}-UbQ-tTA-Dendra2-pA). tTA-Dendra2 was restricted from pCHX17 using *Spe*I/*Bam*HI, and cloned into the corresponding sites (*Nhe*I/*Bam*HI) of pCHX114.	This work
pCHX132	P_{hCMV}-driven UbL-tTA-Dendra2 expression vector (P_{hCMV}-UbL-tTA-Dendra2-pA). tTA-Dendra2 was restricted from pCHX17 using *Spe*I/*Bam*HI, and cloned into the corresponding sites (*Nhe*I/*Bam*HI) of pCHX115.	This work
pCHX138	P_{TRE}-driven UbS-tTA-Dendra2 expression vector (P_{TRE}-UbS-tTA-Dendra2-pA). UbS-tTA-Dendra2 was restricted from pCHX130 with *Eco*RI/*Xba*I and cloned into the corresponding sites (*Eco*RI/*Xba*I) of pMM130.	This work
pCHX142	P_{hCMV}-driven UbR-rtTA-Dendra2 expression vector (P_{hCMV}-UbR-rtTA-Dendra2-pA). rtTA-Dendra2 was restricted from pCHX61 using *Spe*I/*Bam*HI, and cloned into the corresponding sites (*Nhe*I/*Bam*HI) of pCHX75.	This work
pCHX144	P_{hCMV}-driven UbS-rtTA-Dendra2 expression vector (P_{hCMV}-UbS-rtTA-Dendra2-pA). rtTA-Dendra2 was restricted from pCHX61 using *Spe*I/*Bam*HI, and cloned into the corresponding sites (*Nhe*I/*Bam*HI) of pCHX113.	This work
pCHX147	P_{SV40}-driven UbR-tTA-Dendra2 expression vector (P_{SV40}-UbR-tTA-Dendra2-pA). UbR-tTA-Dendra2 was restricted from pCHX91 using *Eco*RI/*Xba*I and cloned into the corresponding sites (*Eco*RI/*Xba*I) of pSEAP2-Control.	This work
pCHX149	P_{SV40}-driven UbS-tTA-Dendra2 expression vector (P_{SV40}-UbS-tTA-Dendra2-pA). UbS-tTA-Dendra2 was restricted from pCHX130 using *Eco*RI/*Xba*I and cloned into the corresponding sites (*Eco*RI/*Xba*I) of pSEAP2-Control.	This work
pCHX150	P_{hCMV}-driven Dendra2 expression vector (P_{hCMV}-Dendra2-pA). Dendra2 was PCR-amplified from p55-H2B-Dendra2 using oligonucleotides OMM266 (5'-gcggaattcaccatgactaGTAACACCCCGGGAATTAACCTG-3', *Eco*RI underlined), and OMM267 (5'-aagctttctagacaccggtggatccgcTAGCCTTGTACACGCCGCTGTCGCC-3', *Xba*I underlined), restricted with *Eco*RI/*Xba*I and cloned into the corresponding sites (*Eco*RI/*Xba*I) of pMM1.	This work

pCHX155	P_{hCMV}-driven tTA-Dendra2-2xPEST expression vector (P_{hCMV}-tTA-Dendra2-2xPEST-pA). PEST was restricted from pMM519 using *Spe*I/*Bam*HI, and cloned into the corresponding sites (*Nhe*I/*Bam*HI) of pCHX33.	This work
pCHX161	P_{PGK}-driven tTA-Dendra2 expression vector (P_{PGK}-tTA-Dendra2-pA). tTA-Dendra2 was restricted from pCHX17 using *Spe*I/*Bam*HI, and cloned into the corresponding sites (*Nhe*I/*Bam*HI) of pMM325.	This work
pCHX163	P_{PGK}-driven UbR-tTA-Dendra2 expression vector. (P_{PGK}-UbR-tTA-Dendra2-pA). UbR-tTA-Dendra2 was restricted from pCHX91 using *Spe*I/*Bam*HI, and cloned into the corresponding sites (*Nhe*I/*Bam*HI) of pMM325.	This work
pCHX165	P_{PGK}-driven UbS-tTA-Dendra2 expression vector (P_{PGK}-UbS-tTA-Dendra2-pA). UbS-tTA-Dendra2 was restricted from pCHX130 using *Spe*I/*Bam*HI, and cloned into the corresponding sites (*Nhe*I/*Bam*HI) of pMM325.	This work
pCHX166	P_{hCMV}-driven UbA expression vector (P_{hCMV}-UbA-pA). UbA was PCR-amplified from pMM505 using oligonucleotides OMM67 (5'-cggaattcaccatgactagtCAGATTTTCGTGAAGACCCTG-3', *Eco*RI underlined), and OMM291 (5'-gctctagacggatccgctagcggcGCCACCTCTCAGGCGAAG-3', *Xba*I underlined), restricted with *Eco*RI/*Xba*I and cloned into the corresponding sites (*Eco*RI/*Xba*I) of pMM1.	This work
pCHX167	P_{hCMV}-driven UbC expression vector (P_{hCMV}-UbC-pA). UbC was PCR-amplified from pMM505 using oligonucleotides OMM67 (5'-cggaattcaccatgactagtCAGATTTTCGTGAAGACCCTG-3', *Eco*RI underlined), and OMM292 (5'-gctctagacggatccgctagcacaGCCACCTCTCAGGCGAAG-3', *Xba*I underlined), restricted with *Eco*RI/*Xba*I and cloned into the corresponding sites (*Eco*RI/*Xba*I) of pMM1.	This work
pCHX168	P_{hCMV}-driven UbD expression vector (P_{hCMV}-UbD-pA). UbD was PCR-amplified from pMM505 using oligonucleotides OMM67 (5'-cggaattcaccatgactagtCAGATTTTCGTGAAGACCCTG-3', *Eco*RI underlined), and OMM295 (5'-gctctagacggatccgctagcgtcGCCACCTCTCAGGCGAAG-3', *Xba*I underlined), restricted with *Eco*RI/*Xba*I and cloned into the corresponding sites (*Eco*RI/*Xba*I) of pMM1.	This work
pCHX169	P_{hCMV}-driven UbE expression vector (P_{hCMV}-UbE-pA). UbE was PCR-amplified from pMM505 using oligonucleotides OMM67 (5'-cggaattcaccatgactagtCAGATTTTCGTGAAGACCCTG-3', *Eco*RI underlined), and OMM293 (5'-gctctagacggatccgctagcttcGCCACCTCTCAGGCGAAG-3', *Xba*I underlined), restricted with *Eco*RI/*Xba*I and cloned into the corresponding sites (*Eco*RI/*Xba*I) of pMM1.	This work
pCHX170	P_{hCMV}-driven UbW expression vector (P_{hCMV}-UbW-pA). UbW was PCR-amplified from pMM505 using oligonucleotides OMM67 (5'-cggaattcaccatgactagtCAGATTTTCGTGAAGACCCTG-3', *Eco*RI underlined), and OCH149 (5'-gctctagacggatccgctagcccaGCCACCTCTCAGGCGAAG-3', *Xba*I underlined), restricted with *Eco*RI/*Xba*I and cloned into the corresponding sites (*Eco*RI/*Xba*I) of pMM1.	This work
pCHX171	P_{hCMV}-driven UbM expression vector (P_{hCMV}-UbM-pA). UbM was PCR-amplified from pMM505 using oligonucleotides OMM67 (5'-cggaattcaccatgactagtCAGATTTTCGTGAAGACCCTG-3', *Eco*RI underlined), and OMM290 (5'-gctctagacggatccgctagccatGCCACCTCTCAGGCGAAG-3', *Xba*I underlined), restricted with *Eco*RI/*Xba*I and cloned into the corresponding sites (*Eco*RI/*Xba*I) of pMM1.	This work
pCHX172	P_{hCMV}-driven UbY expression vector (P_{hCMV}-UbY-pA). UbY was PCR-amplified from pMM505 using oligonucleotides OMM67 (5'-cggaattcaccatgactagtCAGATTTTCGTGAAGACCCTG-3', *Eco*RI	This work

	underlined), and OMM294 (5’-gctctagacggatccgctagcataGCCACCTCTCAGGCGAAG-3’, *Xba*I underlined), restricted with *Eco*RI/*Xba*I and cloned into the corresponding sites (*Eco*RI/*Xba*I) of pMM1.	
pCHX173	P_{hCMV}-driven UbN expression vector (P_{hCMV}-UbN-pA). UbN was PCR-amplified from pMM505 using oligonucleotides OMM67 (5’-cggaattcaccatgactagtCAGATTTTCGTGAAGACCCTG-3’, *Eco*RI underlined), and OMM296 (5’-gctctagacggatccgctagcgttGCCACCTCTCAGGCGAAG-3’, *Xba*I underlined), restricted with *Eco*RI/*Xba*I and cloned into the corresponding sites (*Eco*RI/*Xba*I) of pMM1.	This work
pCHX174	P_{hCMV}-driven UbF expression vector (P_{hCMV}-UbF-pA). UbF was PCR-amplified from pMM505 using oligonucleotides OMM67 (5’-cggaattcaccatgactagtCAGATTTTCGTGAAGACCCTG-3’, *Eco*RI underlined), and OMM297 (5’-gctctagacggatccgctagcaaaGCCACCTCTCAGGCGAAG-3’, *Xba*I underlined), restricted with *Eco*RI/*Xba*I and cloned into the corresponding sites (*Eco*RI/*Xba*I) of pMM1.	This work
pCHX175	P_{hCMV}-driven UbK expression vector (P_{hCMV}-UbK-pA). UbK was PCR-amplified from pMM505 using oligonucleotides OMM67 (5’-cggaattcaccatgactagtCAGATTTTCGTGAAGACCCTG-3’, *Eco*RI underlined), and OMM299 (5’-gctctagacggatccgctagccttGCCACCTCTCAGGCGAAG-3’, *Xba*I underlined), restricted with *Eco*RI/*Xba*I and cloned into the corresponding sites (*Eco*RI/*Xba*I) of pMM1.	This work
pCHX176	P_{hCMV}-driven UbA-tTA-Dendra2 expression vector (P_{hCMV}-UbA-tTA-Dendra2-pA). tTA-Dendra2 was restricted from pCHX17 using *Spe*I/*Bam*HI, and cloned into the corresponding sites (*Nhe*I/*Bam*HI) of pCHX166.	This work
pCHX177	P_{hCMV}-driven UbC-tTA-Dendra2 expression vector (P_{hCMV}-UbC-tTA-Dendra2-pA). tTA-Dendra2 was restricted from pCHX17 using *Spe*I/*Bam*HI, and cloned into the corresponding sites (*Nhe*I/*Bam*HI) of pCHX167.	This work
pCHX178	P_{hCMV}-driven UbD-tTA-Dendra2 expression vector (P_{hCMV}-UbD-tTA-Dendra2-pA). tTA-Dendra2 was restricted from pCHX17 using *Spe*I/*Bam*HI, and cloned into the corresponding sites (*Nhe*I/*Bam*HI) of pCHX168.	This work
pCHX179	P_{hCMV}-driven UbE-tTA-Dendra2 expression vector (P_{hCMV}-UbE-tTA-Dendra2-pA). tTA-Dendra2 was restricted from pCHX17 using *Spe*I/*Bam*HI, and cloned into the corresponding sites (*Nhe*I/*Bam*HI) of pCHX169.	This work
pCHX180	P_{hCMV}-driven UbW-tTA-Dendra2 expression vector (P_{hCMV}-UbW-tTA-Dendra2-pA). tTA-Dendra2 was restricted from pCHX17 using *Spe*I/*Bam*HI, and cloned into the corresponding sites (*Nhe*I/*Bam*HI) of pCHX170.	This work
pCHX181	P_{hCMV}-driven UbM-tTA-Dendra2 expression vector (P_{hCMV}-UbM-tTA-Dendra2-pA). tTA-Dendra2 was restricted from pCHX17 using *Spe*I/*Bam*HI, and cloned into the corresponding sites (*Nhe*I/*Bam*HI) of pCHX171.	This work
pCHX182	P_{hCMV}-driven UbY-tTA-Dendra2 expression vector (P_{hCMV}-UbY-tTA-Dendra2-pA). tTA-Dendra2 was restricted from pCHX17 using *Spe*I/*Bam*HI, and cloned into the corresponding sites (*Nhe*I/*Bam*HI) of pCHX172.	This work

pCHX183	P_{hCMV}-driven UbN-tTA-Dendra2 expression vector (P_{hCMV}-UbN-tTA-Dendra2-pA). tTA-Dendra2 was restricted from pCHX17 using *Spe*I/*Bam*HI, and cloned into the corresponding sites (*Nhe*I/*Bam*HI) of pCHX173.	This work
pCHX184	P_{hCMV}-driven UbF-tTA-Dendra2 expression vector (P_{hCMV}-UbF-tTA-Dendra2-pA). tTA-Dendra2 was restricted from pCHX17 using *Spe*I/*Bam*HI, and cloned into the corresponding sites (*Nhe*I/*Bam*HI) of pCHX174.	This work
pCHX185	P_{hCMV}-driven UbK-tTA-Dendra2 expression vector (P_{hCMV}-UbK-tTA-Dendra2-pA). tTA-Dendra2 was restricted from pCHX17 using *Spe*I/*Bam*HI, and cloned into the corresponding sites (*Nhe*I/*Bam*HI) of pCHX175.	This work
pCHX186	$P_{hEF1\alpha}$-driven tTA-Dendra2 expression vector ($P_{hEF1\alpha}$-tTA-Dendra2-pA). tTA-Dendra2 was restricted from pCHX17 using *Spe*I/*Bam*HI, and cloned into the corresponding sites (*Nhe*I/*Bam*HI) of pVH21.	This work
pCHX188	$P_{hEF1\alpha}$-driven UbR-tTA-Dendra2 expression vector ($P_{hEF1\alpha}$-UbR-tTA-Dendra2-pA). UbR-tTA-Dendra2 was restricted from pCHX91 using *Spe*I/*Bam*HI, and cloned into the corresponding sites (*Nhe*I/*Bam*HI) of pVH21.	This work
pCHX190	$P_{hEF1\alpha}$-driven UbS-tTA-Dendra2 expression vector ($P_{hEF1\alpha}$-UbS-tTA-Dendra2-pA). UbS-tTA-Dendra2 was restricted from pCHX130 using *Spe*I/*Bam*HI, and cloned into the corresponding sites (*Nhe*I/*Bam*HI) of pVH21.	This work
pCHX200	P_{hCMV}-driven 3xUbVR-rtTA-Dendra2 expression vector (P_{hCMV}-3xUbVR-rtTA-Dendra2-pA). rtTA-Dendra2 was restricted from pCHX61 using *Spe*I/*Bam*HI, and cloned into the corresponding sites (*Nhe*I/*Bam*HI) of pCHX35.	This work
pCHX205	$P_{hEF1\alpha}$-driven 3xUbVR-tTA-Dendra2 expression vector ($P_{hEF1\alpha}$-3xUbVR-tTA-Dendra2-pA). 3xUbVR-tTA-Dendra2 was restricted from pCHX50 using *Spe*I/*Bam*HI, and cloned into the corresponding sites (*Nhe*I/*Bam*HI) of pVH21.	This work
pCHX206	P_{SV40}-driven 3xUbVR-tTA-Dendra2 expression vector (P_{SV40}-3xUbVR-tTA-Dendra2-pA). 3xUbVR-tTA-Dendra2 was restricted from pCHX50 using *Eco*RI/*Xba*I and cloned into the corresponding sites (*Eco*RI/*Xba*I) of pSEAP2-Control.	This work
pCHX209	P_{hCMV}-driven UbD-rtTA-Dendra2 expression vector (P_{hCMV}-UbD-rtTA-Dendra2-pA). rtTA-Dendra2 was restricted from pCHX61 using *Spe*I/*Bam*HI, and cloned into the corresponding sites (*Nhe*I/*Bam*HI) of pCHX168.	This work
pCHX204	P_{hCMV}-driven UbM-rtTA-Dendra2 expression vector (P_{hCMV}-UbM-rtTA-Dendra2-pA). tTA-Dendra2 was restricted from pCHX61 using *Spe*I/*Bam*HI, and cloned into the corresponding sites (*Nhe*I/*Bam*HI) of pCHX171.	This work
pCHX213	P_{hCMV}-driven UbK-rtTA-Dendra2 expression vector (P_{hCMV}-UbK-rtTA-Dendra2-pA). tTA-Dendra2 was restricted from pCHX61 using *Spe*I/*Bam*HI, and cloned into the corresponding sites (*Nhe*I/*Bam*HI) of pCHX175.	This work

pCHX221	P_{SV40}-driven UbD-tTA-Dendra2 expression vector (P_{SV40}-UbD-tTA-Dendra2-pA). UbD-tTA-Dendra2 was restricted from pCHX178 using *Eco*RI/*Xba*I and cloned into the corresponding sites (*Eco*RI/*Xba*I) of pSEAP2-Control.	This work
pCHX222	P_{SV40}-driven UbM-tTA-Dendra2 expression vector (P_{SV40}-UbM-tTA-Dendra2-pA). UbM-tTA-Dendra2 was restricted from pCHX181 using *Eco*RI/*Xba*I and cloned into the corresponding sites (*Eco*RI/*Xba*I) of pSEAP2-Control.	This work
pCHX223	$P_{hEF1\alpha}$-driven UbD-tTA-Dendra2 expression vector ($P_{hEF1\alpha}$-UbD-tTA-Dendra2-pA). UbD-tTA-Dendra2 was restricted from pCHX178 using *Spe*I/*Bam*HI, and cloned into the corresponding sites (*Nhe*I/*Bam*HI) of pVH21.	This work
pCHX224	$P_{hEF1\alpha}$-driven UbM-tTA-Dendra2 expression vector ($P_{hEF1\alpha}$-UbM-tTA-Dendra2-pA). UbM-tTA-Dendra2 was restricted from pCHX181 using *Spe*I/*Bam*HI, and cloned into the corresponding sites (*Nhe*I/*Bam*HI) of pVH21.	This work
pCHX225	P_{PGK}-driven UbD-tTA-Dendra2 expression vector (P_{PGK}-UbD-tTA-Dendra2-pA). UbD-tTA-Dendra2 was restricted from pCHX178 using *Spe*I/*Bam*HI, and cloned into the corresponding sites (*Nhe*I/*Bam*HI) of pMM325.	This work
pCHX226	P_{PGK}-driven UbM-tTA-Dendra2 expression vector (P_{PGK}-UbM-tTA-Dendra2-pA). UbM-tTA-Dendra2 was restricted from pCHX181 using *Spe*I/*Bam*HI, and cloned into the corresponding sites (*Nhe*I/*Bam*HI) of pMM325.	This work
pCHX227	P_{TRE}-driven UbD-tTA-Dendra2 expression vector (P_{TRE}-UbD-tTA-Dendra2-pA). UbD-tTA-Dendra2 was restricted from pCHX178 with *Eco*RI/*Xba*I and cloned into the corresponding sites (*Eco*RI/*Xba*I) of pMM130.	This work
pCHX228	P_{TRE}-driven UbM-tTA-Dendra2 expression vector (P_{TRE}-UbM-tTA-Dendra2-pA). UbM-tTA-Dendra2 was restricted from pCHX181 with *Eco*RI/*Xba*I and cloned into the corresponding sites (*Eco*RI/*Xba*I) of pMM130.	This work
pCHX229	P_{PGK}-driven UbK-tTA-Dendra2 expression vector (P_{PGK}-UbK-tTA-Dendra2-pA). UbK-tTA-Dendra2 was restricted from pCHX185 using *Spe*I/*Bam*HI, and cloned into the corresponding sites (*Nhe*I/*Bam*HI) of pMM325.	This work
pCHX230	$P_{hEF1\alpha}$-driven UbK-tTA-Dendra2 expression vector ($P_{hEF1\alpha}$-UbK-tTA-Dendra2-pA). UbK-tTA-Dendra2 was restricted from pCHX185 using *Spe*I/*Bam*HI, and cloned into the corresponding sites (*Nhe*I/*Bam*HI) of pVH21.	This work
pCHX231	P_{SV40}-driven UbK-tTA-Dendra2 expression vector (P_{SV40}-UbK-tTA-Dendra2-pA). UbK-tTA-Dendra2 was restricted from pCHX185 using *Eco*RI/*Xba*I and cloned into the corresponding sites (*Eco*RI/*Xba*I) of pSEAP2-Control.	This work
pCHX235	P_{PGK}-driven 3xUbVR-tTA-Dendra2 expression vector (P_{PGK}-3xUbVR-tTA-Dendra2-pA). 3xUbVR-tTA-Dendra2 was restricted from pCHX50 using *Spe*I/*Bam*HI, and cloned into the corresponding sites (*Nhe*I/*Bam*HI) of pMM325.	This work

pCHX246	P_{hCMV}-driven 3xUbVR-tTA-Dendra2 and mCherry expression vector (P_{hCMV}-3xUbVR-tTA-Dendra2-P2A-mCherry-pA). 3xUbVR-tTA-Dendra2 was PCR-amplified from pCHX50 using oligonucleotides OMM67 (5’-cggaattcaccatgactagtCAGATTTTCGTGAAGACCCTG-3’, *Eco*RI underlined), and OCH157 (5’-CTCCAGCCTGCTTCAGCAGGCTGAAGTTAGTAGCT CCGCTTCCGTACACGCCGCTGTCGCCGGA-3’), mCherry was PCR-amplified from pFS29 using oligonucleotides OCH158 (5’-CCTGCTGAAGCAGGCTGGAGACGTGGAGGAGAACCCTG GACCTATGGTGAGCAAGGGCGAGGAG-3’) and OCH159 (5’-gctctagacggatccgctagc TCATCCAGACTTGTACAGCTCGTC-3’, *Xba*I underlined). Both fragments were joined by overlapping PCR using oligonucleotides OMM67 and OCH159, restricted with *Eco*RI/*Xba*I and cloned into the corresponding sites (*Eco*RI/*Xba*I) of pMM1.	This work
pCHX247	P_{hCMV}-driven UbR-tTA-Dendra2 and mCherry expression vector (P_{hCMV}-UbR-tTA-Dendra2-P2A-mCherry-pA). tTA-Dendra2-P2A-mCherry was restricted from pCHX252 using *Spe*I/*Xba*I and cloned into the corresponding sites (*Nhe*I/*Xba*I) of pCHX75.	This work
pCHX248	P_{hCMV}-driven UbS-tTA-Dendra2 and mCherry expression vector (P_{hCMV}-UbS-tTA-Dendra2-P2A-mCherry-pA). tTA-Dendra2-P2A-mCherry was restricted from pCHX252 using *Spe*I/*Xba*I and cloned into the corresponding sites (*Nhe*I/*Xba*I) of pCHX113.	This work
pCHX249	P_{hCMV}-driven UbK-tTA-Dendra2 and mCherry expression vector (P_{hCMV}-UbK-tTA-Dendra2-P2A-mCherry-pA). tTA-Dendra2-P2A-mCherry was restricted from pCHX252 using *Spe*I/*Xba*I and cloned into the corresponding sites (*Nhe*I/*Xba*I) of pCHX175.	This work
pCHX250	P_{hCMV}-driven UbD-tTA-Dendra2 and mCherry expression vector (P_{hCMV}-UbD-tTA-Dendra2-P2A-mCherry-pA). tTA-Dendra2-P2A-mCherry was restricted from pCHX252 using *Spe*I/*Xba*I and cloned into the corresponding sites (*Nhe*I/*Xba*I) of pCHX168.	This work
pCHX251	P_{hCMV}-driven UbM-tTA-Dendra2 and mCherry expression vector (P_{hCMV}-UbM-tTA-Dendra2-P2A-mCherry-pA). tTA-Dendra2-P2A-mCherry was restricted from pCHX252 using *Spe*I/*Xba*I and cloned into the corresponding sites (*Nhe*I/*Xba*I) of pCHX171.	This work
pCHX252	P_{hCMV}-driven tTA-Dendra2 and mCherry expression vector (P_{hCMV}-tTA-Dendra2-P2A-mCherry-pA). tTA-Dendra2 was PCR-amplified from pCHX17 using oligonucleotides OTS507 (5’-ctgaactagtggtggttctGGTTCCAGATTAGATAAAAGTAAAGTGATTAACAGCGCATTAGAGC-3’, *Spe*I underlined), and OCH157 (5’-CTCCAGCCTGCTTCAGCAGGCTGAAGTTAGTAGCT CCGCTTCCGTACACGCCGCTGTCGCCGGA-3’), mCherry was PCR-amplified from pFS29 using oligonucleotides OCH158 (5’-CCTGCTGAAGCAGGCTGGAGACGTGGAGGAGAACCCTG GACCTATGGTGAGCAAGGGCGAGGAG-3’) and OCH159 (5’-gctctagacggatccgctagc TCATCCAGACTTGTACAGCTCGTC-3’, *Xba*I underlined). Both fragments were joined by overlapping PCR using oligonucleotides OTS507 and OCH159, restricted with *Spe*I/*Xba*I and cloned into the corresponding sites (*Spe*I/*Xba*I) of pMM1.	This work

pCHX253	P_{TRE}-driven 3xUbVR-tTA-Dendra2 expression vector (P_{TRE}-3xUbVR-tTA-Dendra2-pA). 3xUbVR-tTA-Dendra2 was restricted from pCHX50 with *Eco*RI/*Xba*I and cloned into the corresponding sites (*Eco*RI/*Xba*I) of pMM130.	This work
pCHX254	P_{TRE}-driven UbK-tTA-Dendra2 expression vector (P_{TRE}-UbK-tTA-Dendra2-pA). UbK-tTA-Dendra2 was restricted from pCHX185 with *Eco*RI/*Xba*I and cloned into the corresponding sites (*Eco*RI/*Xba*I) of pMM130.	This work
pCHX255	P_{hCMV}-driven 3xUbVR-L7Ae expression vector (P_{hCMV}-3xUbVR-L7Ae-pA). L7Ae was restricted from pMM546 with *Spe*I/*Bam*HI, and cloned into the corresponding sites (*Nhe*I/*Bam*HI) of pCHX35.	This work
pCHX256	P_{hCMV}-driven UbR-L7Ae expression vector (P_{hCMV}-UbR-L7Ae-pA). L7Ae was restricted from pMM546 with *Spe*I/*Bam*HI, and cloned into the corresponding sites (*Nhe*I/*Bam*HI) of pCHX75.	This work
pCHX257	P_{hCMV}-driven UbS-L7Ae expression vector (P_{hCMV}-UbS-L7Ae-pA). L7Ae was restricted from pMM546 with *Spe*I/*Bam*HI, and cloned into the corresponding sites (*Nhe*I/*Bam*HI) of pCHX113.	This work
pCHX258	P_{hCMV}-driven UbK-L7Ae expression vector (P_{hCMV}-UbK-L7Ae-pA). L7Ae was restricted from pMM546 with *Spe*I/*Bam*HI, and cloned into the corresponding sites (*Nhe*I/*Bam*HI) of pCHX175.	This work
pCHX259	P_{hCMV}-driven UbD-L7Ae expression vector (P_{hCMV}-UbD-L7Ae-pA). L7Ae was restricted from pMM546 with *Spe*I/*Bam*HI, and cloned into the corresponding sites (*Nhe*I/*Bam*HI) of pCHX168.	This work
pCHX260	P_{hCMV}-driven UbM-L7Ae expression vector (P_{hCMV}-UbM-L7Ae-pA). L7Ae was restricted from pMM546 with *Spe*I/*Bam*HI, and cloned into the corresponding sites (*Nhe*I/*Bam*HI) of pCHX171.	This work
pCHX261	P_{hCMV}-driven 3xUbVR-Fast-FT expression vector (P_{hCMV}-3xUbVR-Fast-FT-pA). Fast-FT was restricted from pMM531 with *Spe*I/*Bam*HI, and cloned into the corresponding sites (*Nhe*I/*Bam*HI) of pCHX35.	This work
pCHX266	P_{hCMV}-driven UbM-Fast-FT expression vector (P_{hCMV}-UbM-Fast-FT-pA). Fast-FT was restricted from pMM531 with *Spe*I/*Bam*HI, and cloned into the corresponding sites (*Nhe*I/*Bam*HI) of pCHX171.	This work
pCHX273	P_{TRE}-C/D_{box}-driven 3xUbVR-Fast-FT expression vector (P_{TRE}-C/D_{box}-3xUbVR-Fast-FT-pA). 3xUbVR-Fast-FT was restricted from pCHX261 with *Eco*RI/*Xba*I, and cloned into the corresponding sites (*Eco*RI/*Xba*I) of pMM345.	This work
pCHX278	P_{TRE}-C/D_{box}-driven UbM-Fast-FT expression vector (P_{TRE}-C/D_{box}-UbM-Fast-FT-pA). UbM-Fast-FT was restricted from pCHX266 with *Eco*RI/*Xba*I, and cloned into the corresponding sites (*Eco*RI/*Xba*I) of pMM345.	This work
pCHX287	P_{TRE}-driven 3xUbVR-L7Ae expression vector (P_{TRE}-3xUbVR-L7Ae-pA). 3xUbVR-L7Ae was excised from pCHX255 with *Eco*RI/*Xba*I and cloned into the corresponding sites (*Eco*RI/*Xba*I) of pMM130.	This work
pCHX288	P_{TRE}-driven UbR-L7Ae expression vector (P_{TRE}-UbR-L7Ae-pA). UbR-L7Ae was excised from pCHX256 with *Eco*RI/*Xba*I and cloned into the corresponding sites (*Eco*RI/*Xba*I) of pMM130.	This work
pCHX289	P_{TRE}-driven UbS-L7Ae expression vector (P_{TRE}-UbS-L7Ae-pA). UbS-L7Ae was excised from pCHX257 with *Eco*RI/*Xba*I and cloned into the corresponding sites (*Eco*RI/*Xba*I) of pMM130.	This work
pCHX290	P_{TRE}-driven UbK-L7Ae expression vector (P_{TRE}-UbK-L7Ae-pA). UbK-L7Ae was excised from pCHX258 with *Eco*RI/*Xba*I and cloned into the corresponding sites (*Eco*RI/*Xba*I) of pMM130.	This work

pCHX291	P_{TRE}-driven UbD-L7Ae expression vector (P_{TRE}-UbD-L7Ae-pA). UbD-L7Ae was excised from pCHX259 with *Eco*RI/*Xba*I and cloned into the corresponding sites (*Eco*RI/*Xba*I) of pMM130.	This work
pCHX292	P_{TRE}-driven UbM-L7Ae expression vector (P_{TRE}-UbM-L7Ae-pA). UbM-L7Ae was excised from pCHX260 with *Eco*RI/*Xba*I and cloned into the corresponding sites (*Eco*RI/*Xba*I) of pMM130.	This work
pCHX293	P_{TtgR1}-driven 3xUbVR-L7Ae expression vector (P_{TtgR1}-3xUbVR-L7Ae-pA). 3xUbVR-L7Ae was restricted from pCHX255 with *Eco*RI/*Hin*dIII and cloned into the corresponding sites (*Eco*RI/ *Hin*dIII) of pMG10.	This work
pCHX294	P_{TtgR1}-driven UbM-L7Ae expression vector (P_{TtgR1}-UbM-L7Ae-pA). L7Ae was restricted from pCH260 with *Eco*RI/*Hin*dIII and cloned into the corresponding sites (*Eco*RI/*Hin*dIII) of pMG10.	This work
pCHX299	P_{TtgR1}-driven 3xUbVR-L7Ae-P2A-Citrine expression vector (P_{TtgR1}-3xUbVR-L7Ae-P2A-Citrine-pA). 3xUbVR-L7Ae was PCR-amplified from pCHX293 using oligonucleotides OCH162 (5'-cgcgacgcgtCAGTATTTACAAACAACCATG-3') and OCH160 (5'-CTCCAGCCTGCTTCAGCAGGCTGAAGTTAGTAGCTCCGCTTCCGACACCGGTGGATCCGCTAGC-3'), Citrine was PCR-amplified from pMM545 using oligonucleotides OCH161 (5'-CCTGCTGAAGCAGGCTGGAGACGTGGAGGAGAACCCTGGACCTATGACTAGTGGTGGTTCTGGT-3') and OMM140 (5'-aagcttttctagacaccggtggatccgctagcAGAACCCTTGTACAGCTCGTCCATGCC-3', *Hin*dIII underlined). Both fragments were joined by overlapping PCR using oligonucleotides OCH162 and OMM140, restricted with *Eco*RI/*Hin*dIII and cloned into the corresponding sites (*Eco*RI/*Hin*dIII) of pMG10.	This work
pCHX300	P_{TtgR1}-driven 3xUbVR-L7Ae-P2A-Citrine expression vector and P_{TRE}-C/D_{box}-driven 3xUbVR-Fast-FT expression vector (P_{TtgR1}-3xUbVR-L7Ae-P2A-Citrine-pA:P_{TRE}-C/D_{box}-3xUbVR-Fast-FT-pA). P_{TtgR1}-3xUbVR-L7Ae-P2A-Citrine-pA was PCR-amplified from pCHX299 using oligonucleotides OCH162 (5'-cgcgacgcgtCAGTATTTACAAACAACCATG-3', *Mlu*I underlined) and OCH163 (5'-cgcgctcgagAAAAAACCTCCCACACCTCCC-3', *Xho*I underlined), restricted with *Mlu*I/*Xho*I and cloned into the corresponding sites (*Mlu*I/*Xho*I) of pCHX273.	This work
pCHX301	P_{TRE}-driven TtgR-VP16 expression vector and P_{hCMV}-driven rtTA expression vector (P_{TRE}-TtgR-VP16-pA:P_{hCMV}-rtTA-pA). P_{TRE}-TtgR-VP16-pA was PCR-amplified from pMM334 using oligonucleotides OCH164 (5'-cgcgacgcgtTCGAGCTCGGTACCCGGGTCG-3', *Mlu*I underlined) and OCH163 (5'-cgcgctcgagAAAAAACCTCCCACACCTCCC-3', *Xho*I underlined), restricted with *Mlu*I/*Xho*I and cloned into the corresponding sites (*Mlu*I/*Xho*I) of pMM591.	This work
pCHX303	P_{TtgR1}-driven UbM-L7Ae-P2A-Citrine expression vector (P_{TtgR1}-UbM-L7Ae-P2A-Citrine-pA). UbM-L7Ae was PCR-amplified from pCHX294 using oligonucleotides OCH162 (5'-cgcgacgcgtCAGTATTTACAAACAACCATG-3') and OCH160 (5'-CTCCAGCCTGCTTCAGCAGGCTGAAGTTAGTAGCTCCGCTTCCGACACCGGTGGATCCGCTAGC-3'), Citrine was PCR-amplified from pMM545 using oligonucleotides OCH161 (5'-CCTGCTGAAGCAGGCTGGAGACGTGGAGGAGAACCCTGGACCTATGACTAGTGGTGGTTCTGGT-3') and OMM140 (5'-aagcttttctagacaccggtggatccgctagcAGAACCCTTGTACAGCTCGTCCATGCC-3', *Hin*dIII underlined). Both fragments were joined by overlapping PCR using oligonucleotides OCH162	This work

	and OMM140, restricted with *Eco*RI/*Hin*dIII and cloned into the corresponding sites (*Eco*RI/*Hin*dIII) of pMG10.	
pCHX304	P_{TtgR1}-driven UbS-L7Ae-P2A-Citrine expression vector (P_{TtgR1}-UbS-L7Ae-P2A-Citrine-pA). UbS-L7Ae was PCR-amplified from pCHX257 using oligonucleotides OMM67 (5'-cggaattcaccatgactagtCAGATTTTCGTGAAGACCCTG-3', *Eco*RI underlined) and OCH160 (5'-CTCCAGCCTGCTTCAGCAGGCTGAAGTTAGTAGCTCCGCTTCCGACACCGGTGGATCCGCTAGC-3'), Citrine was PCR-amplified from pMM545 using oligonucleotides OCH161 (5'-CCTGCTGAAGCAGGCTGGAGACGTGGAGGAGAACCCTGGACCTATGACTAGTGGTGGTTCTGGT-3') and OMM140 (5'-aagcttttctagacaccggtggatccgctagcAGAACCCTTGTACAGCTCGTCCATGCC-3', *Hin*dIII underlined). Both fragments were joined by overlapping PCR using oligonucleotides OMM67 and OMM140, restricted with *Eco*RI/*Hin*dIII and cloned into the corresponding sites (*Eco*RI/*Hin*dIII) of pMG10.	This work
pCHX305	P_{TtgR1}-driven UbK-L7Ae-P2A-Citrine expression vector (P_{TtgR1}-UbK-L7Ae-P2A-Citrine-pA). UbK-L7Ae was PCR-amplified from pCHX258 using oligonucleotides OMM67 (5'-cggaattcaccatgactagtCAGATTTTCGTGAAGACCCTG-3', *Eco*RI underlined) and OCH160 (5'-CTCCAGCCTGCTTCAGCAGGCTGAAGTTAGTAGCTCCGCTTCCGACACCGGTGGATCCGCTAGC-3'), Citrine was PCR-amplified from pMM545 using oligonucleotides OCH161 (5'-CCTGCTGAAGCAGGCTGGAGACGTGGAGGAGAACCCTGGACCTATGACTAGTGGTGGTTCTGGT-3') and OMM140 (5'-aagcttttctagacaccggtggatccgctagcAGAACCCTTGTACAGCTCGTCCATGCC-3', *Hin*dIII underlined). Both fragments were joined by overlapping PCR using oligonucleotides OMM67 and OMM140, restricted with *Eco*RI/*Hin*dIII and cloned into the corresponding sites (*Eco*RI/*Hin*dIII) of pMG10.	This work
pCHX306	P_{TtgR1}-driven UbR-L7Ae-P2A-Citrine expression vector (P_{TtgR1}-UbR-L7Ae-P2A-Citrine-pA). UbR-L7Ae was PCR-amplified from pCHX256 using oligonucleotides OMM67 (5'-cggaattcaccatgactagtCAGATTTTCGTGAAGACCCTG-3', *Eco*RI underlined) and OCH160 (5'-CTCCAGCCTGCTTCAGCAGGCTGAAGTTAGTAGCTCCGCTTCCGACACCGGTGGATCCGCTAGC-3'), Citrine was PCR-amplified from pMM545 using oligonucleotides OCH161 (5'-CCTGCTGAAGCAGGCTGGAGACGTGGAGGAGAACCCTGGACCTATGACTAGTGGTGGTTCTGGT-3') and OMM140 (5'-aagcttttctagacaccggtggatccgctagcAGAACCCTTGTACAGCTCGTCCATGCC-3', *Hin*dIII underlined). Both fragments were joined by overlapping PCR using oligonucleotides OMM67 and OMM140, restricted with *Eco*RI/*Hin*dIII and cloned into the corresponding sites (*Eco*RI/*Hin*dIII) of pMG10.	This work
pCHX307	P_{TtgR1}-driven UbD-L7Ae-P2A-Citrine expression vector (P_{TtgR1}-UbD-L7Ae-P2A-Citrine-pA). UbD-L7Ae was PCR-amplified from pCHX259 using oligonucleotides OMM67 (5'-cggaattcaccatgactagtCAGATTTTCGTGAAGACCCTG-3', *Eco*RI underlined) and OCH160 (5'-CTCCAGCCTGCTTCAGCAGGCTGAAGTTAGTAGCTCCGCTTCCGACACCGGTGGATCCGCTAGC-3'), Citrine was PCR-amplified from pMM545 using oligonucleotides OCH161 (5'-	This work

	CCTGCTGAAGCAGGCTGGAGACGTGGAGGAGAACCCTGGACCTATGACTAGTGGTGGTTCTGGT-3’) and OMM140 (5’-aagctttctagacaccggtggatccgctagcAGAACCCTTGTACAGCTCGTCCATGCC-3’, *Hin*dIII underlined). Both fragments were joined by overlapping PCR using oligonucleotides OMM67 and OMM140, restricted with *Eco*RI/*Hin*dIII and cloned into the corresponding sites (*Eco*RI/*Hin*dIII) of pMG10.	
pCHX308	P_{TtgR1}-driven UbM-L7Ae-P2A-Citrine expression vector (P_{TtgR1}-UbM-L7Ae-P2A-Citrine-pA) and P_{TRE}-C/D_{box}-driven 3xUbVR-Fast-FT expression vector (P_{TtgR1}-UbM-L7Ae-P2A-Citrine-pA:P_{TRE}-C/D_{box}-3xUbVR-Fast-FT-pA). P_{TtgR1}-UbM-L7Ae-P2A-Citrine-pA was PCR-amplified from pCHX303 using oligonucleotides OCH162 (5’-cgcgacgcgtCAGTATTTACAAACAACCATG-3’, *Mlu*I underlined) and OCH163 (5’-cgcgctcgagAAAAAACCTCCCACACCTCCC-3’, *Xho*I underlined), restricted with *Mlu*I/*Xho*I and cloned into the corresponding sites (*Mlu*I/*Xho*I) of pCHX273.	This work
pCHX309	P_{TtgR1}-driven UbS-L7Ae-P2A-Citrine expression vector (P_{TtgR1}-UbS-L7Ae-P2A-Citrine-pA) and P_{TRE}-C/D_{box}-driven 3xUbVR-Fast-FT expression vector (P_{TtgR1}-UbS-L7Ae-P2A-Citrine-pA:P_{TRE}-C/D_{box}-3xUbVR-Fast-FT-pA). P_{TtgR1}-UbS-L7Ae-P2A-Citrine-pA was PCR-amplified from pCHX304 using oligonucleotides OCH162 (5’-cgcgacgcgtCAGTATTTACAAACAACCATG-3’, *Mlu*I underlined) and OCH163 (5’-cgcgctcgagAAAAAACCTCCCACACCTCCC-3’, *Xho*I underlined), restricted with *Mlu*I/*Xho*I and cloned into the corresponding sites (*Mlu*I/*Xho*I) of pCHX273.	This work
pCHX310	P_{TtgR1}-driven UbK-L7Ae-P2A-Citrine expression vector (P_{TtgR1}-UbK-L7Ae-P2A-Citrine-pA) and P_{TRE}-C/D_{box}-driven 3xUbVR-Fast-FT expression vector (P_{TtgR1}-UbK-L7Ae-P2A-Citrine-pA:P_{TRE}-C/D_{box}-3xUbVR-Fast-FT-pA). P_{TtgR1}-UbK-L7Ae-P2A-Citrine-pA was PCR-amplified from pCHX305 using oligonucleotides OCH162 (5’-cgcgacgcgtCAGTATTTACAAACAACCATG-3’, *Mlu*I underlined) and OCH163 (5’-cgcgctcgagAAAAAACCTCCCACACCTCCC-3’, *Xho*I underlined), restricted with *Mlu*I/*Xho*I and cloned into the corresponding sites (*Mlu*I/*Xho*I) of pCHX273.	This work
pCHX311	P_{TtgR1}-driven UbR-L7Ae-P2A-Citrine expression vector (P_{TtgR1}-UbR-L7Ae-P2A-Citrine-pA) and P_{TRE}-C/D_{box}-driven 3xUbVR-Fast-FT expression vector (P_{TtgR1}-UbR-L7Ae-P2A-Citrine-pA:P_{TRE}-C/D_{box}-3xUbVR-Fast-FT-pA). P_{TtgR1}-UbR-L7Ae-P2A-Citrine-pA was PCR-amplified from pCHX305 using oligonucleotides OCH162 (5’-cgcgacgcgtCAGTATTTACAAACAACCATG-3’, *Mlu*I underlined) and OCH163 (5’-cgcgctcgagAAAAAACCTCCCACACCTCCC-3’, *Xho*I underlined), restricted with *Mlu*I/*Xho*I and cloned into the corresponding sites (*Mlu*I/*Xho*I) of pCHX273.	This work
pCHX312	P_{TtgR1}-driven UbD-L7Ae-P2A-Citrine expression vector (P_{TtgR1}-UbD-L7Ae-P2A-Citrine-pA) and P_{TRE}-C/D_{box}-driven 3xUbVR-Fast-FT expression vector (P_{TtgR1}-UbD-L7Ae-P2A-Citrine-pA:P_{TRE}-C/D_{box}-3xUbVR-Fast-FT-pA). P_{TtgR1}-UbD-L7Ae-P2A-Citrine-pA was PCR-amplified from pCHX303 using oligonucleotides OCH162 (5’-cgcgacgcgtCAGTATTTACAAACAACCATG-3’, *Mlu*I underlined) and OCH163 (5’-cgcgctcgagAAAAAACCTCCCACACCTCCC-3’, *Xho*I underlined), restricted with *Mlu*I/*Xho*I and cloned into the corresponding sites (*Mlu*I/*Xho*I) of pCHX273.	This work
pLeox2	P_{hCMV}-driven dCas9-Dendra2 expression vector (P_{hCMV}-dCas9-Dendra2-pA). Dendra2 was restricted from pCHX150 with *Spe*I/*Hind*III and cloned into the corresponding sites (*Nhe*I/*Hind*III) of pVH333.	This work

pLeox3	P_{hCMV}-driven MCP-VPR-Dendra2 expression vector (P_{hCMV}-MCP-VPR-Dendra2-pA). Dendra2 was restricted from pCHX150 with *Spe*I/*Hind*III and cloned into the corresponding sites (*Nhe*I/*Hind*III) of pVH323.	This work
pLeox5	P_{hCMV}-driven 3xUbVR-dCas9-Dendra2 expression vector (P_{hCMV}-3xUbVR-dCas9-Dendra2-pA). 3xUbVR was restricted from pCHX35 with *Eco*RI/*Nhe*I and cloned into the corresponding sites (*Eco*RI/*Spe*I) of pLeox2.	This work
pLeox6	P_{hCMV}-driven 3xUbVR-MCP-VPR-Dendra2 expression vector (P_{hCMV}-3xUbVR-MCP-VPR-Dendra2-pA). 3xUbVR was restricted from pCHX35 with *Eco*RI/*Nhe*I and cloned into the corresponding sites (*Eco*RI/*Spe*I) of pLeox3.	This work
pLeox7	P_{hCMV}-driven UbR-dCas9-Dendra2 expression vector (P_{hCMV}-UbR-dCas9-Dendra2-pA). UbR was restricted from pCHX75 with *Eco*RI/*Nhe*I and cloned into the corresponding sites (*Eco*RI/*Spe*I) of pLeox2.	This work
pLeox9	P_{hCMV}-driven UbR-MCP-VPR-Dendra2 expression vector (P_{hCMV}-UbR-MCP-VPR-Dendra2-pA). UbR was restricted from pCHX75 with *Eco*RI/*Nhe*I and cloned into the corresponding sites (*Eco*RI/*Spe*I) of pLeox3.	This work
pLeox11	P_{hCMV}-driven UbD-dCas9-Dendra2 expression vector (P_{hCMV}-UbD-dCas9-Dendra2-pA). UbD was restricted from pCHX168 with *Eco*RI/*Nhe*I and cloned into the corresponding sites (*Eco*RI/*Spe*I) of pLeox2.	This work
pLeox12	P_{hCMV}-driven UbD-MCP-VPR-Dendra2 expression vector (P_{hCMV}-UbD-MCP-VPR-Dendra2-pA). UbD was restricted from pCHX168 with *Eco*RI/*Nhe*I and cloned into the corresponding sites (*Eco*RI/*Spe*I) of pLeox3.	This work
pLeox14	P_{hCMV}-driven UbM-dCas9-Dendra2 expression vector (P_{hCMV}-UbM-dCas9-Dendra2-pA). UbM was restricted from pCHX171 with *Eco*RI/*Nhe*I and cloned into the corresponding sites (*Eco*RI/*Spe*I) of pLeox2.	This work
pLeox15	P_{hCMV}-driven UbM-MCP-VPR-Dendra2 expression vector (P_{hCMV}-UbM-MCP-VPR-Dendra2-pA). UbM was restricted from pCHX171 with *Eco*RI/*Nhe*I and cloned into the corresponding sites (*Eco*RI/*Spe*I) of pLeox3.	This work
pLeox17	P_{hCMV}-driven UbK-dCas9-Dendra2 expression vector (P_{hCMV}-UbK-dCas9-Dendra2-pA). UbK was restricted from pCHX175 with *Eco*RI/*Nhe*I and cloned into the corresponding sites (*Eco*RI/*Spe*I) of pLeox2.	This work
pLeox18	P_{hCMV}-driven UbK-MCP-VPR-Dendra2 expression vector (P_{hCMV}-UbK-MCP-VPR-Dendra2-pA). UbK was restricted from pCHX175 with *Eco*RI/*Nhe*I and cloned into the corresponding sites (*Eco*RI/*Spe*I) of pLeox3.	This work
pLeox20	P_{hCMV}-driven UbS-dCas9-Dendra2 expression vector (P_{hCMV}-UbS-dCas9-Dendra2-pA). UbS was restricted from pCHX113 with *Eco*RI/*Nhe*I and cloned into the corresponding sites (*Eco*RI/*Spe*I) of pLeox2.	This work
pLeox21	P_{hCMV}-driven UbS-MCP-VPR-Dendra2 expression vector (P_{hCMV}-UbS-MCP-VPR-Dendra2-pA). UbS was restricted from pCHX113 with *Eco*RI/*Nhe*I and cloned into the corresponding sites (*Eco*RI/*Spe*I) of pLeox3.	This work

Abbreviations: **B3**, *Zygosaccharomyces bisporus* recombinase; **C/D_{box}**, RNA motif specifically binding to the L7Ae protein; **dCas9,** *Streptococcus pyogenes* dead CRISPR associated protein 9; **Citrine**, improved version of EYFP; **CRISPR**, clustered regularly interspaced short palindromic repeats; **EYFP**, enhanced yellow fluorescent protein; **FT**, fluorescent timer; **GCaMP6s**, ultrasensitive protein calcium sensor; **GFP,** green fluorescent protein; **H2B**, histone cluster 2; **lacS**,

lac repressor protein spacer; **L7Ae**, archaeal ribosomal protein L7Ae; **mCherry**, *Discosoma*-derived red fluorescent protein; **MCS**, multiple cloning site; miR124**,** microRNA precursor 124; **MCP,** RNA-binding coat protein from the bacteriophage MS2; **P2A**, porcine teschovirus-1 2A self-cleaving peptide; **p65,** transactivating subunit of NF-kappa B; **pA**, polyadenylation signal; **PEST**, mouse ornithine decarboxylase-derived peptide sequence rich in proline (P), glutamic acid (E), serine (S), and threonine (T) acting as a protein degradation signal; **PESTmod**, modified PEST; **PCR**, polymerase chain reaction; **P_{CAG}**, chicken β-actin promoter; **$P_{hEF1\alpha}$**, human elongation factor-1 alpha promoter; **P_{ETR2},** macrolide-responsive promoter; **P_{H1},** human histone 1 promoter; **P_{hCMV}**, human cytomegalovirus immediate early promoter; **$P_{hCMVmin}$**, minimal version of P_{hCMV}; **$P_{hCMV*-1}$**, tTA-specific tetracycline-responsive promoter; **P_{hINS},** human insulin promoter (-881 to +54); **P_{hU6},** human U6 promoter; **P_{PGK}**, murine phosphoglycerate kinase 1 promoter; **P_{TRE}**, tTA-specific tetracycline-responsive promoter; **P_{TRT}**, L-tryptophan-responsive promoter; **P_{TtgR1}**, phloretin-responsive promoter; **rtTA**, reverse tetracycline-dependent transactivator; **P_{SPA}**, SCB1-responsive promoter (O_{papRI}-$P_{hCMVmin}$); **P_{SV40}**, simian virus 40 promoter; **Rta,** Epstein-Barr virus R transactivator; **SEAP**, human placental secreted alkaline phosphatase; **sgRNA,** single guide RNA; **shRNA**, short hairpin RNA; **tetR**, *Escherichia coli* Tn10-derived tetracycline repressor; **tTA**, tetracycline-dependent transactivator (tetR-VP16); **TtgR**, repressor of the *Pseudomonas putida* DOT-T1E ABC multidrug efflux pump; **Ub**, ubiquitin degradation signal; **UbAR**, ubiquitin fusion construct in which alanine is introduced at the G76 residue of the C-terminal isopeptidase site and fused to the transcription factor such that Ub is partly cleaved from the fusion partner after translation to reveal arginine at the N terminus of the transcription factor; **UbAV**, ubiquitin fusion construct in which alanine is introduced at the G76 residue of the C-terminal isopeptidase site and fused to the transcription factor such that Ub is partly cleaved from the fusion partner after translation to reveal valine at the N terminus of the trancription factor; **UbVR**, ubiquitin fusion construct in which valine is introduced at the G76 residue of the C-terminal isopeptidase site and fused to the transcription factor such that Ub is partly cleaved from the fusion partner after translation to reveal arginine at the N terminus of the trancription factor; **UbVV**, ubiquitin fusion construct in which valine is introduced at the G76 residue of the C-terminal isopeptidase site and fused to the transcription factor such that Ub is partly cleaved from the fusion partner after translation to reveal valine at the N terminus of the trancription factor; **UbX**, ubiquitin fusion construct with its C-terminal isopeptidase site intact fused to the transcription factor such that ubiquitin is cleaved from the fusion partner after translation to reveal an amino acid (X) at the N terminus of the trancription factor (X: R, P, W, H, I, K, Q, V, L, D, N, G, Y, T, S, F, A, C, E, M); **VP16**, *Herpes simplex*-derived transactivation domain; **VP64,** *Herpes simplex*-derived tetrameric VP16 transactivation domain; **VPR,** hybrid VP64-p65-Rta tripartite activator.
Oligonucleotides: Restriction endonuclease-specific sites are shown in lower case underlined, annealing base pairs are indicated in capital letters.

References

1. Mohr, *et al.* Labeling cellular structures in vivo using confined primed conversion of photoconvertible fluorescent proteins. *Nature Protocols **11**, 2419–2431* (2016).
2. Wertz, *et al.* Single-cell–initiated monosynaptic tracing reveals layer-specific cortical network modules. *Science **349**, 70-74* (2015).
3. Auslaender, *et al.* A general design strategy for protein-responsive riboswitches in mammalian cells. *Nature Methods 11, 1154–1160* (2014).
4. Gitzinger, *et al.* Controlling transgene expression in subcutaneous implants using a skin lotion containing the apple metabolite phloretin. *PNAS **106**, 10638–10643* (2009).
5. Müller, *et al*. Designed cell consortia as fragrance-programmable analog-to-digital converters. *Nature Chemical Biolog **13**, 309–316* (2017).
6. Tigges, *et al.* A tunable synthetic mammalian oscillator. *Nature **457**, 309–312* (2009).
7. Ausländer, *et al.* Programmable single-cell mammalian biocomputers. *Nature **487**, 123–127* (2012).
8. Pasque, et al. Histone variant macroH2A confers resistance to nuclear reprogramming. *The EMBO Journal **30**, 2373-2387* (2011).
9. Konermann, *et al.* Genome-scale transcriptional activation by an engineered CRISPR-Cas9 complex. *Nature **517**, 583–588* (2015).
10. Subach, *et al*. Monomeric fluorescent timers that change color from blue to red report on cellular trafficking. *Nature Chemical Biology **5**, 118–126* (2009).
11. Bacchus, *et al.* Synthetic two-way communication between mammalian cells. *Nature Biotechnology **30**, 991–996* (2012).
